# The 14‐3‐3 Protein SlTFT1 Accelerates Tomato Fruit Ripening by Binding and Stabilising YFT1 in the Ethylene Signalling Pathway

**DOI:** 10.1111/pbi.70274

**Published:** 2025-07-22

**Authors:** Tengjian Wen, Lichun Cao, Lida Zhang, Lingxia Zhao

**Affiliations:** ^1^ Department of Plant Science, School of Agriculture and Biology Shanghai Jiao Tong University Shanghai China; ^2^ School of Agriculture and Biology, Joint Tomato Research Institute Shanghai Jiao Tong University Shanghai China; ^3^ Joint International Research Laboratory of Metabolic & Developmental Sciences, State Key Laboratory of Hybrid Rice, School of Life Sciences and Biotechnology Shanghai Jiao Tong University Shanghai China

**Keywords:** carotenoid accumulation, ethylene signal transduction, fruit ripening, SlETP2‐like3, SlTFT1, tomato, YFT1

## Abstract

Ethylene plays a pivotal role during the fruit ripening process in tomato ( *Solanum lycopersicum* ). Previously, we have reported that the tomato EIN2‐like protein YELLOW‐FRUITED TOMATO1 (YFT1), a core component in the ethylene signal transduction pathway, exerts a critical regulatory function in the tomato fruit ripening process. However, the molecular mechanism of YFT1‐mediated ethylene signalling during tomato ripening still remains largely unknown. In this study, we performed yeast two‐hybrid screens of a cDNA expression library from tomato fruits and obtained a new YFT1‐interacting protein, SlTFT1 (TOMATO FOURTEEN‐THREE PROTEIN 1), and confirmed their interaction in vitro and in vivo. Further bimolecular fluorescence complementation (BiFC) assays indicated that SlTFT1 can specifically bind the canonical and non‐canonical on the carboxyl terminus of YFT1 (YFT1‐C). Triple response assays and ethylene responsive gene expression analysis demonstrated that SlTFT1 positively regulates the ethylene signalling pathway in a YFT1‐dependent manner. Phenotypical and biochemical analysis of SlTFT1 overexpression lines and loss‐of‐function mutants showed that *SlTFT1* significantly accelerated ethylene emission, chromoplast development, lycopene accumulation and fruit ripening rates; however, the deletion of *YFT1* and/or *SlTFT1* lesion generated the opposite results, suggesting that *SlTFT1* positively regulates tomato fruit ripening also in a YFT1‐dependent manner. Co‐expression and western blotting assays showed that SlTFT1 can efficiently prevent YFT1 from protein degradation mediated by an F‐box protein SlETP2‐like3, suggesting that SlTFT1 can stabilise YFT1 through their physical interaction. Collectively, our results reveal a genetic and molecular framework of the SlTFT1‐YFT1 complex, which modulates tomato fruit ripening by regulating ethylene signalling.

## 1 Introduction

Ethylene, a small molecule gaseous phytohormone, plays fundamental roles in various plant biological processes such as seed germination, plant growth, flower/fruit development (Corbineau et al. 2014; Dubois et al. 2018; Etchells et al. 2012; Feng et al. 2020; Wuriyanghan et al. 2009) and responses to biotic and abiotic stresses (Clarke et al. 2009; Du et al. 2014; Guan et al. 2015; Perata 2020; Tao et al. 2015; Thomashow 2010). Ethylene signal transduction is initiated from the perception of the ethylene molecule by ethylene receptors (ETRs) (Clark et al. 1998; Hua et al. 1995; Tieman et al. 2000). The perception and signal transduction of ethylene have been well characterised in *Arabidopsis thaliana* by using various ethylene mutants (Binder 2020).

In the absence of ethylene, the ethylene receptors can interact with and activate a Raf‐like serine/threonine kinase of the CONSTITUTIVE TRIPLE RESPONSE 1 (CTR1) anchored at the endoplasmic reticulum (ER) (Gao et al. 2003; Kieber et al. 1993). Subsequently, the ETHYLENE‐INSENSITIVE2 (EIN2), a centrally positive regulator in ethylene signal transduction (Alonso et al. 1999), could be phosphorylated by the active CTR1 and turned into an inactive conformation (Ju et al. 2012; Qiao et al. 2012), and then was degraded by the ubiquitin/26S proteasome system (UPS) through mediating by two F‐box proteins of EIN2‐TARGETING PROTEIN1/2 (ETP1/2) (Qiao et al. 2009). In the presence of ethylene, the small molecule gaseous phytohormone is perceived and captured by ethylene receptors, and the ETRs lose the ability to activate CTR1 due to carrying ethylene molecules. As a result, the inactive CTR1 cannot phosphorylate the EIN2 protein, which would be cleaved at the unphosphorylated Ser^645^ by an unknown enzyme, thus releasing the free C‐terminus (EIN2‐C) from the anchoring ER into the cytoplasm (Huang et al. 2022; Ju et al. 2012; Qiao et al. 2012; Wen et al. 2012). The EIN2‐C fragments are translocated into the nucleus and the processing body (p‐body). In the nucleus, EIN2‐C forms a complex with transcription factors such as ETHYLENE‐INSENSITIVE3 (EIN 3) and ETHYLENE‐INSENSITIVE3‐Like (EILs), and the complex binds to ETHYLENE RESPONSIVE ELEMENTS (EREs) residing at the regulatory regions of target genes to regulate their expression, and the plants exhibit ethylene‐responsive phenotypes (Ju et al. 2012; Qiao et al. 2012; Wen et al. 2012). The p‐body complex containing EIN2‐C, EIN5 and poly A binding proteins (PABs) binds to the 3′ untranslated regions (3′‐UTRs) of *EIN3‐BINDING F‐BOX PROTEIN1/2* (*EBF1/2*) mRNAs to impede their protein synthesis (Li et al. 2015; Merchante et al. 2015). The F‐box proteins, EBF1 and EBF2, mediate the EIN3/EIL1 degradation through the UPS pathway (Guo and Ecker 2003; Potuschak et al. 2003).

EIN2 is a core component in the ethylene signalling pathway. Its encoding gene *EIN2* was first isolated from *Arabidopsis* ethylene‐insensitive *ein2*‐null mutants (Alonso et al. 1999; Guzmán and Ecker 1990). EIN2‐C, cooperating with EIN3, exhibits its function to regulate plant epigenetics by elevating histone acetylation levels (Shao et al. 2024; Wang and Qiao 2019; Wang et al. 2021; Zhang et al. 2017). Distinct phosphorylation forms of EIN2 can precisely switch between glucose‐TOR (target of rapamycin) energy and ethylene signalling (Fu et al. 2021). So far, it has been revealed that the EIN2‐C polypeptide delivers the ethylene signal to the nucleus through two ways, including “cleave/shuttle” and translational suppression of EBF1/2 proteins (Ji and Guo 2013; Ju et al. 2012; Qiao et al. 2012; Wen et al. 2012; Zhao et al. 2023).

Tomato ( *Solanum lycopersicum* ) is a leading commercial crop in the whole world and is directly related to vegetable supply and human diet. Moreover, tomato is also used as an ideal model plant in basic scientific research, such as genetics (Li et al. 2023; Tanksley 2004), omics (Alba et al. 2005; Kaur et al. 2023; Lin et al. 2014; Shinozaki et al. 2018), evolutionary biology (Sun et al. 2023; Moyle 2008), reproductive biology (Bedinger et al. 2011; Wu et al. 2024), fruit growth and development (Osorio et al. 2011) and fruit ripening (Chen et al. 2020; Ito et al. 2017).

As an essential regulator, ethylene triggers the onset of tomato fruit ripening, which is controlled by two consecutive biological processes, including ethylene synthesis and signal transduction (Barry et al. 2005; Giovannoni 2007; Li et al. 2025; Ma et al. 2012; Osorio et al. 2011; Vrebalov et al. 2002). In our previous reports, we isolated the tomato *YELLOW‐FRUITED TOMATO 1* (*YFT1*) gene, which encodes an *Arabidopsis* EIN2 homologous protein, from a *yellow‐fruited tomato 1* (*yft1*, original *n3122*) mutant by a map‐based cloning approach (Gao et al. 2016). We found that the *YFT1* allele was severely down‐regulated in *yft1* tomato due to an insertion of a 573‐bp fragment at the 5′‐UTR, which leads to the suppression of ethylene synthesis and inhibition of ethylene signal transduction, and the mutant tomato exhibited ethylene‐insensitive phenotypes including delayed fruit ripening and reduced carotenoid accumulation (Zhao et al. 2020). However, the functions of *YFT1* in ethylene signalling are still elusive. To this end, we screened a cDNA expression library of tomato fruit using the C‐terminal portion of YFT1 (YFT1‐C) as bait by the yeast two‐hybrid approach and isolated a 14‐3‐3 protein SlTFT1, which could interact with YFT1‐C. We found that *SlTFT1* was specifically expressed during fruit ripening in tomato, implying that SlTFT1 is perhaps involved in the tomato fruit ripening process.

The 14‐3‐3 proteins are a class of highly conserved, acidic, soluble proteins and are widely distributed in eukaryotic organisms. They generally interact with and bind the client proteins that in most but not all cases need to be phosphorylated to exert their regulatory functions (Ma et al. 2023; Ormancey et al. 2017; Zhao, Li, and Li 2021). As regulatory proteins, the 14‐3‐3s regulate almost all aspects of plant life activities. 14‐3‐3s modulate nitrogen metabolic pathways by interacting with alanine‐glyoxylate aminotransferase (AGT) in *Arabidopsis* (Shin et al. 2011) and also regulate photomorphogenesis or phototropic growth through binding to PIF3 and NPH3, respectively (Song et al. 2023; Sullivan et al. 2021). OsGF14f positively modulates osmotic stress tolerance and abscisic acid (ABA) responses by interacting with OsbZIP23 in rice (Ma et al. 2023). In the BR signalling pathway, 14‐4‐3s can activate BR receptor kinase (BRI1) (Lee et al. 2020) and bind to the phosphorylated BZR1 to retain BZR1 in the cytoplasm, which can inhibit BR signal transduction in plants (Bai et al. 2007). 14‐3‐3s can activate MAP kinase cascades in *Arabidopsis* and can enhance plant disease resistance (Dong et al. 2023). As a key member, 14‐3‐3 forms an ternary ‘florigen activation complex’ (FAC) with FLOWERING LOCUS T (FT) and bZIP transcription factor (FD), and the FAC induces transcription of the *OsMADS15* gene to cause early flowering in rice (Taoka et al. 2011). Nevertheless, we have limited knowledge concerning how the 14‐3‐3 proteins modulate ethylene signalling transduction and affect fruit ripening in tomato.

In this study, SlTFT1, which is a 14‐3‐3 protein in tomato, was identified through yeast two‐hybrid screening using YFT1‐C as a bait. The interaction between SlTFT1 and YFT1‐C was further validated by bimolecular fluorescence complementation (BiFC), firefly luciferase complementation imaging (LCI) and co‐immunoprecipitation (co‐IP) assays. Null mutation and overexpression of the *SlTFT1* gene could efficiently alter ethylene signal transduction and ethylene synthesis, eventually affecting the tomato fruit ripening rate. Our biochemical assays showed that SlTFT1 could significantly stabilise the YFT1 protein, probably through preventing an F‐box protein SlETP2‐3‐like‐mediated degradation.

This work sheds new light on the molecular mechanisms of an ethylene‐independent 14‐3‐3 protein, SlTFT1, which participates in the regulation of ethylene signal transduction and the fruit ripening process in a YFT1‐dependent manner in tomato.

## 2 Results

### 2.1 SlTFT1 Interacts With YFT1

**FIGURE 1:**
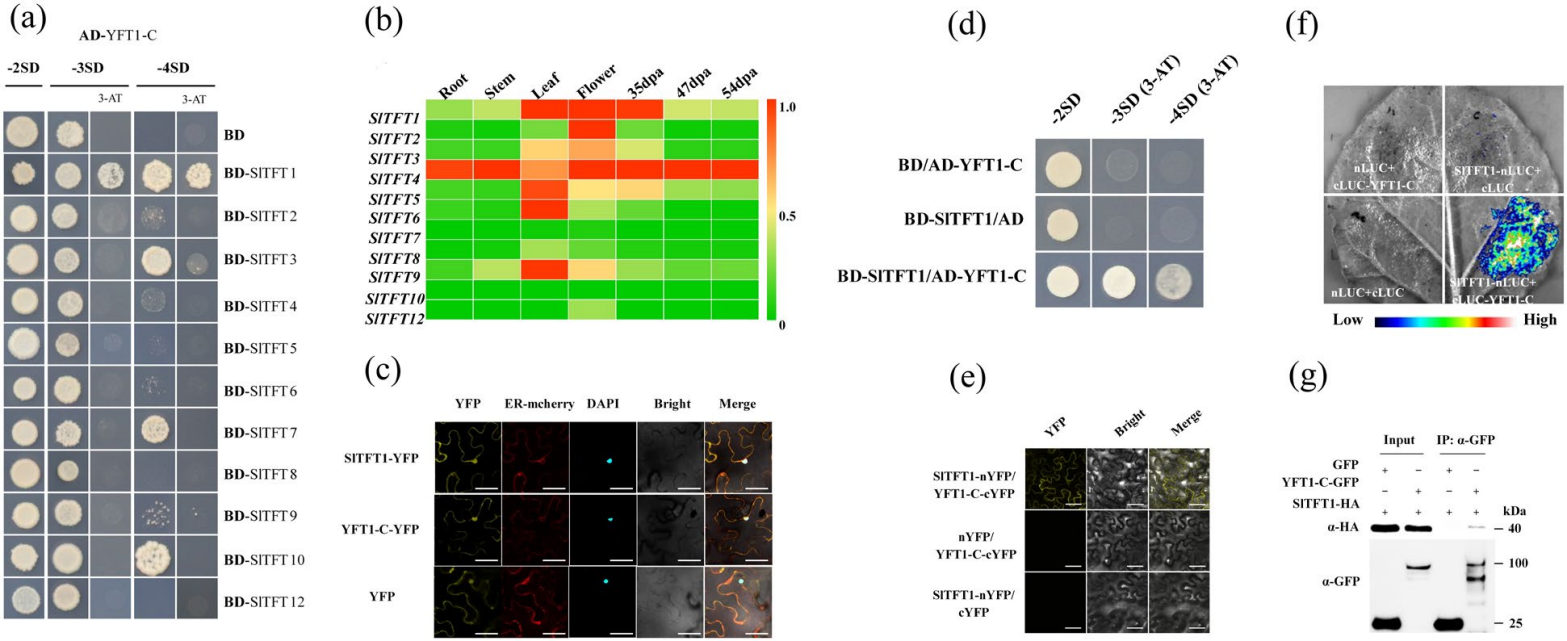
Physical interaction between SlTFT1 and YFT1‐C. (a) Analysis of interactions of SlTFTs and YFT1‐C by yeast two‐hybrid. Each CDS sequence of *SlTFT* genes was respectively fused to the GAL4 DNA binding domain (BD) and co‐transformed with AD‐YFT1‐C in yeast strain AH109. −2SD, SD‐Trp‐Leu; −3SD, SD‐Trp‐Leu‐His; −4SD, SD‐Trp‐Leu‐His‐Ade; 3‐AT, 3 amino‐1,2,4‐triazole. The positive yeast clones on −2SD, −3SD/−3SD + 5 mM 3‐AT and −4SD/−4SD + 5 mM 3‐AT were picked and sequenced. BD, the empty pGBKT7 plasmid. (b) The expression profiles of *SlTFTs* in tomato fruits. Total RNA was extracted from each organ, and the relative expression was determined by RT‐qPCR analysis. dpa, days post‐anthesis. The *SlTFT* with different numbers indicates the family members encoding putative14‐3‐3 proteins in tomato. (c) Subcellular localisation of SlTFT1 and YFT1‐C proteins. DAPI, 4′,6‐diamidino‐2‐phenylindole; ER‐mCherry, the ER marker ER‐rk CD3‐959 fused with mCherry; SlTFT1‐YFP, SlTFT1 protein fused at the amino terminal of YFP; YFP, yellow fluorescent protein; YFT1‐C‐YFP, YFT1‐C protein fused at the amino terminal of YFP. Scale bars, 100 μm. (d) Interaction of SlTFT1 with YFT1‐C determined by yeast two‐hybrid assay. The transformed yeast cells were grown on solid media of −2SD, −3SD with 5 mM 3‐AT and −4SD with 5 mM 3‐AT. AD and BD indicate the empty plasmids of pGADT7 carrying transcription activation domain (AD) and pGBKT7 containing DNA binding domain (BD). (e) BiFC assay in transiently transformed *N. benthamiana* leaf epidermal cells. cYFP, the C‐terminal half of YFP; nYFP, the N‐terminal half of YFP; SlTFT1‐nYFP, the CDS sequence encoding the nYFP polypeptide was fused upstream of the *SlTFT1* CDS sequence; YFT1‐C‐cYFP, the CDS sequence encoding the cYFP polypeptide was fused downstream of the *YFT1‐C* CDS sequence. Scale bars, 100 μm. (f) Split luciferase complementary assays in tobacco leaf. cLuc, C‐terminal luciferase; cLUC‐YFT1‐C, the CDS sequence encoding the C‐terminal half of LUC polypeptide was fused upstream of the YFT1‐C CDS; LUC, luciferase; nLuc, N‐terminal luciferase; SlTFT1‐nLUC, the CDS sequence encoding the N‐terminal half of LUC polypeptide was fused downstream to *SlTFT1* CDS. (g) Co‐immunoprecipitation (Co‐IP) assay. The constructs carrying *SlTFT1‐HA* and *YFT1‐C‐GFP* were transiently co‐transformed into *N. benthamiana* leaf epidermal cells. The total soluble proteins extracted from the infected tobacco leaves were used to immune‐precipitate with GFP affinity agarose beads, and the target proteins were detected using rabbit anti‐GFP and rabbit anti‐HA. “+” and “−” indicate the presence and absence of the corresponding proteins.

Since YFT1 functions as a core component in ethylene signalling transduction (Zhao et al. 2023), we used its C‐terminal portion (YFT1‐C) as bait to screen a cDNA library of tomato (cv. M82) fruits at 47 dpa (days post‐anthesis). We obtained five positive clones, *Solyc11g010470*, and the other four, and further confirmed the interactions with YFT1‐C using yeast two‐hybrid assays (Figure S1a). The expression profile analyses indicated that *Solyc11g010470* has a specific higher expression in tomato fruit compared with the other four genes. The *Solyc11g010470* gene encodes a tomato 14‐3‐3 protein (Fourteen‐Three‐Three isoform 1, SlTFT1) (Figure S1b,c). There are 11 *TFT* genes (*SlTFTs*) in the tomato genome. We tested the physical interactions of YFT1‐C with all SlTFTs and found that SlTFT1 strongly interacted with YFT1‐C (Figure 1a). RT‐qPCR analyses indicated that *SlTFT1* was highly expressed in tomato fruit, flower and leaf (Figure 1b), and there was no expression difference of *SlTFT1* in M82 and ethylene‐insensitive *yft1* mutant during the tomato ripening process (Figure S3). Those results implied that *SlTFT1* is ethylene‐independent at the transcriptional level and perhaps involved in the tomato fruit ripening process.

The analyses of subcellular localisation in tobacco leaf cells showed that both SlTFT1 and YFT1‐C proteins were located at both the endoplasmic reticulum and the nucleus (Figure 1c). Furthermore, the *in silico* mining of the Tomato Expression Atlas pipeline (SGN‐TEA, http://tea.solgenomics.net/) indicated that both *SlTFT1* and *YFT1* were simultaneously expressed during tomato fruit maturation (Figure S1b,c). Moreover, the physical interactions between SlTFT1 and YFT1‐C were validated by in *vivo* and in *vitro* assays, including yeast two‐hybrid (Y2H), BiFC, LCI and Co‐IP (Figure 1d–g). These results showed that SlTFT1 can interact with YFT1‐C protein, and they may function together in ethylene‐regulated fruit ripening.

### 2.2 SlTFT1 Interacts With YFT1‐C via Both Canonical and Non‐Canonical Motifs

**FIGURE 2:**
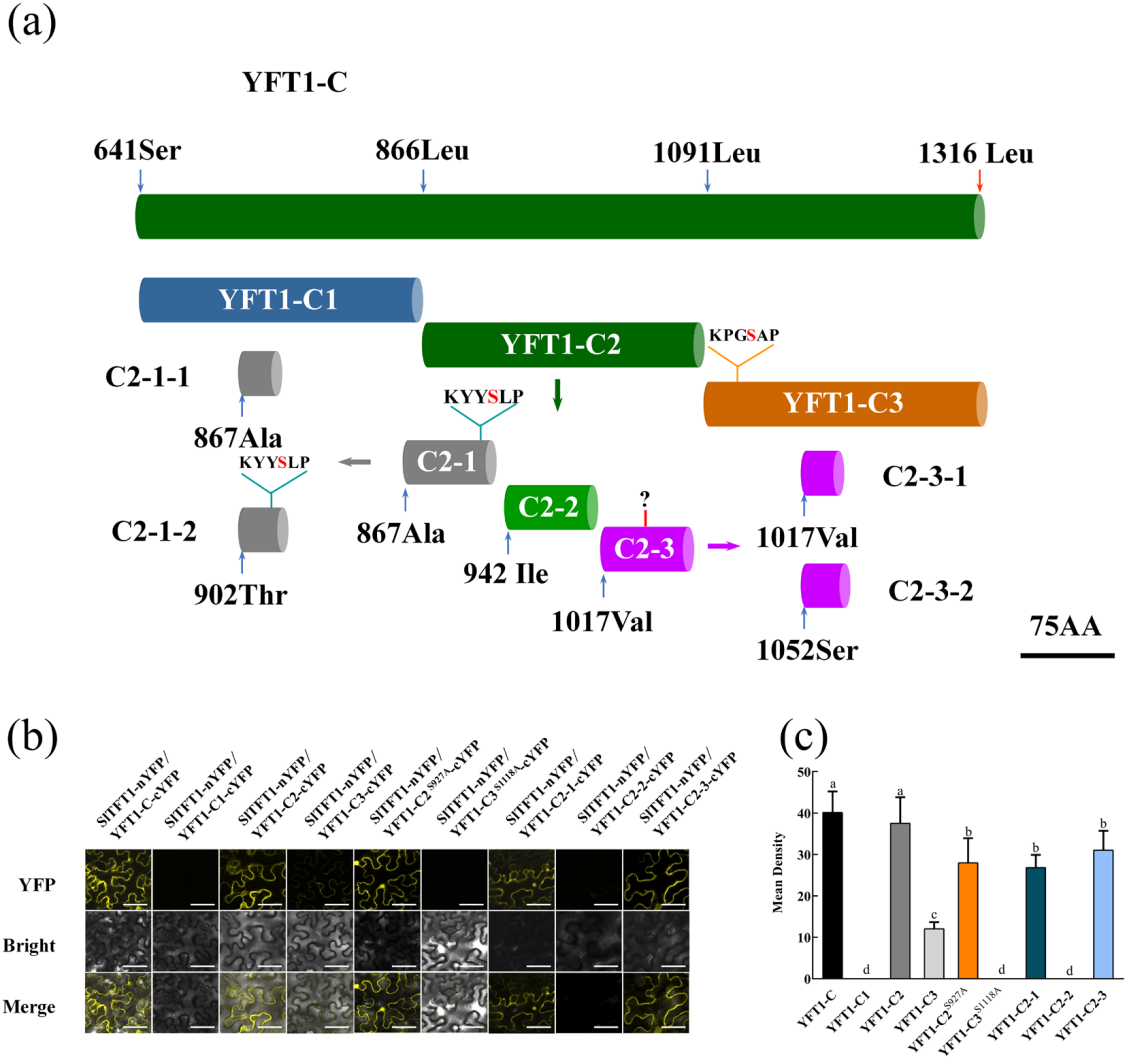
Identification of SlTFT1 protein binding regions at YFT1‐C. (a) Derivatives of the truncated YFT1‐C. A YFT1‐C protein containing 676 AA was cut at 866 Leu and 1091 Ala sites of YFT1‐C to be divided into three equal length segments of YFT1‐C1 (226AA), YFT1‐C2 (225AA) and YFT1‐C3 (225AA). The YFT1‐C2 was cut at 942 Ile and 1017 Val sites in YFT1‐C2 protein segment to be further divided into YFT1‐C2‐1/2/3 (all with 75AA). The polypeptides of YFT1‐C2‐1/3 were further divided into YFT1‐C2‐1‐1/2 (35AA/40AA) or YFT1‐C2‐3‐1/2 (35AA/40AA) at 902 Thr or 1052 Ser sites, respectively. The string letters of ‘KYYSLP’ and ‘KPGSAP’ indicate the canonical binding motifs of 14–3‐3 protein at the client proteins, they are located at the YFT1‐C2‐1 and YFT1‐C3, respectively. ? indicates the unidentified canonical or non‐canonical binding motifs of 14–3‐3 protein. Scale bar, 75 AA. (b) BiFC assay. In total, nine derivatives of *YFT1‐C* CDS sequences were separately fused with the encoding sequence of cYFP to create plasmids of the *YFT1‐C‐cYFP*, *YFT1‐C1‐cYFP*, *YFT1‐C2‐cYFP*, *YFT1‐C3‐cYFP*, *YFT1‐C2* ^*927A*^ *‐cYFP*, *YFT1‐C3* ^*1118A*^ *‐cYFP*, *YFT1‐C2‐1‐cYFP*, *YFT1‐C2‐2‐cYFP* and *YFT1‐C2‐3‐cYFP*, which were co‐transformed into tobacco with nYFP‐SlTFT1, respectively. In here, YFT1‐C2^S927A^ and YFT1‐C3^S1118A^ indicate the site mutations of phosphorylation residues. YFP fluorescence signals were observed using a confocal laser scanning microscope. Scale bars, 100 μm. (c) Fluorescence density of interaction of SlTFT1 with YFT1‐C derivatives. The YFP signals derived from all nine combinations of the SlTFT1‐nYFP with YFT1‐C/YFT1‐C1/YFT1‐C2/YFT1‐C3/YFT1‐C2^S927A^/YFT1‐^S1118A^C3/YFT1‐C2‐1/YFT1‐C2‐2/YFT1‐C2‐3‐cYFP were calculated by Image J software. Error bars indicate standard deviation (SD). Lowercase letters indicate statistical significance at the *p* < 0.05 level determined by the Duncan test (*n* = 10 fields derived 5 biological replicates).

To determine the accurate SlTFT1 binding regions or motifs at YFT1‐C protein, we equally divided the YFT1‐C protein into three segments (YFT1‐C1, YFT1‐C2 and YFT1‐C3) (Figure 2a). The encoding DNA sequences were inserted into the upstream of cYFP in pXY104‐cYFP plasmid, respectively. The resulted constructs were transiently co‐transformed with pXY106‐nYFP‐SlTFT1 into tobacco leaves mediated via *Agrobacteria*. The results showed that the YFP signals could be detected in both YFT1‐C2‐cYFP/nYFP‐SlTFT1 and YFT1‐C3‐cYFP/nYFP‐SlTFT1 but not in YFT1‐C1‐cYFP/nYFP‐SlTFT1. Moreover, the YFP fluorescence intensity analysis showed that the YFT1‐C2‐cYFP/nYFP‐SlTFT1 interaction was significantly stronger than YFT1‐C3‐cYFP/nYFP‐SlTFT1 (Figure 2b,c).

The 14‐3‐3 proteins are well known to bind phosphopeptides. There are three types of specific 14‐3‐3 canonical binding motifs, including R/KXXpS/pTX (mode I), R/KXXXpS/pTXP (mode II) and motif p (S/T) X1‐2‐COOH (mode III) (Huang et al. 2018; Ma et al. 2023; Ormancey et al. 2017). To further analyse the binding motif of SlTFT1, we searched for the above three canonical motifs in the predictive YFT1‐C amino acid sequence. Two canonical 14‐3‐3 binding motifs (KYY**S**LP and KPG**S**AP) were detected at YFT1‐C2‐1 (923–929 aa) and YFT1‐C3 (1114–1120 aa), and they are the mode II category (Figure 2a).

To examine whether the phosphorylation statuses of the canonical motifs affect the interaction of SlTFT1 and YFT1‐C, we created YFT1‐C2^S927A^ (KYY(**S/A**)LP) and YFT1‐C3^S1118A^ (KPG(**S/A**)AP) mutants. The interactions of YFT1‐C2^S927A^/SlTFT1 and YFT1‐C3^S1118A^/SlTFT1 were detected using the BiFC assay. The results showed that the YFT1‐C2^S927A^‐cYFP/nYFP‐SlTFT1 could generate a YFP fluorescence signal, although its YFP fluorescence strength was weaker than YFT1‐C2‐cYFP/nYFP‐SlTFT1. On the other hand, the YFP fluorescence signal was not detected in YFT1‐C3^S1118A^/nYFP‐SlTFT1 (Figure 2b,c). These results suggest that phosphorylation on S1118 on the KPG**S**AP motif at YFT1‐C3 is required for the binding of SlTFT1, while phosphorylation on S927 of the KYY**S**LP motif is not indispensable for the interaction of SlTFT1 and YFT1. Given that the phosphorylation of serine/threonine is necessary for the canonical motif function, we hypothesised that there are some non‐canonical motifs mediating the YFT1‐C2/SlTFT1 interaction. To confirm this, we further divided YFT1‐C2 into three small segments with 75 amino acids: YFT1‐C2‐1/2/3. The BiFC results showed that YFT1‐C2‐1/3 but not YFT1‐C2‐2 can interact with SlTFT1 (Figure 2b,c), suggesting that at least one non‐canonical motif at YFT1‐C2‐3 may also be required for the interaction of SlTFT1 and YFT1. Further substitution and truncation of the YFT1‐C were conducted to detect interaction with SlTFT1; the BiFC results showed that YFT1‐C^S927A/S1118A^ seriously repressed (66.97% of the YFT1‐C) but YFT1‐C^S927D/S1118D^ enhanced (113.92% of the YFT1‐C) interactions of SlTFT1 and YFT1‐C (Figure S2a,b). The YFT1‐C2‐1 and YFT1‐C2‐3 were further divided into YFT1‐C2‐1‐1/2 and YFT1‐C2‐3‐1/2, and we found that all four polypeptides can interact with SlTFT1, although only YFT1‐C2‐1‐2 harbours a canonical binding motif of KYYSLP (923–929 aa) (Figure 2a and Figure S2d). These results further imply that SlTFT1 can interact with its client protein YFT1‐C via both the canonical and non‐canonical motifs. However, we could not detect the direct transcriptional regulation/interaction between SlTFT1 and YFT1 using DLR and EMSA approaches (Figure S6).

### 2.3 SlTFT1 Stabilises YFT1‐C Protein

**FIGURE 3:**
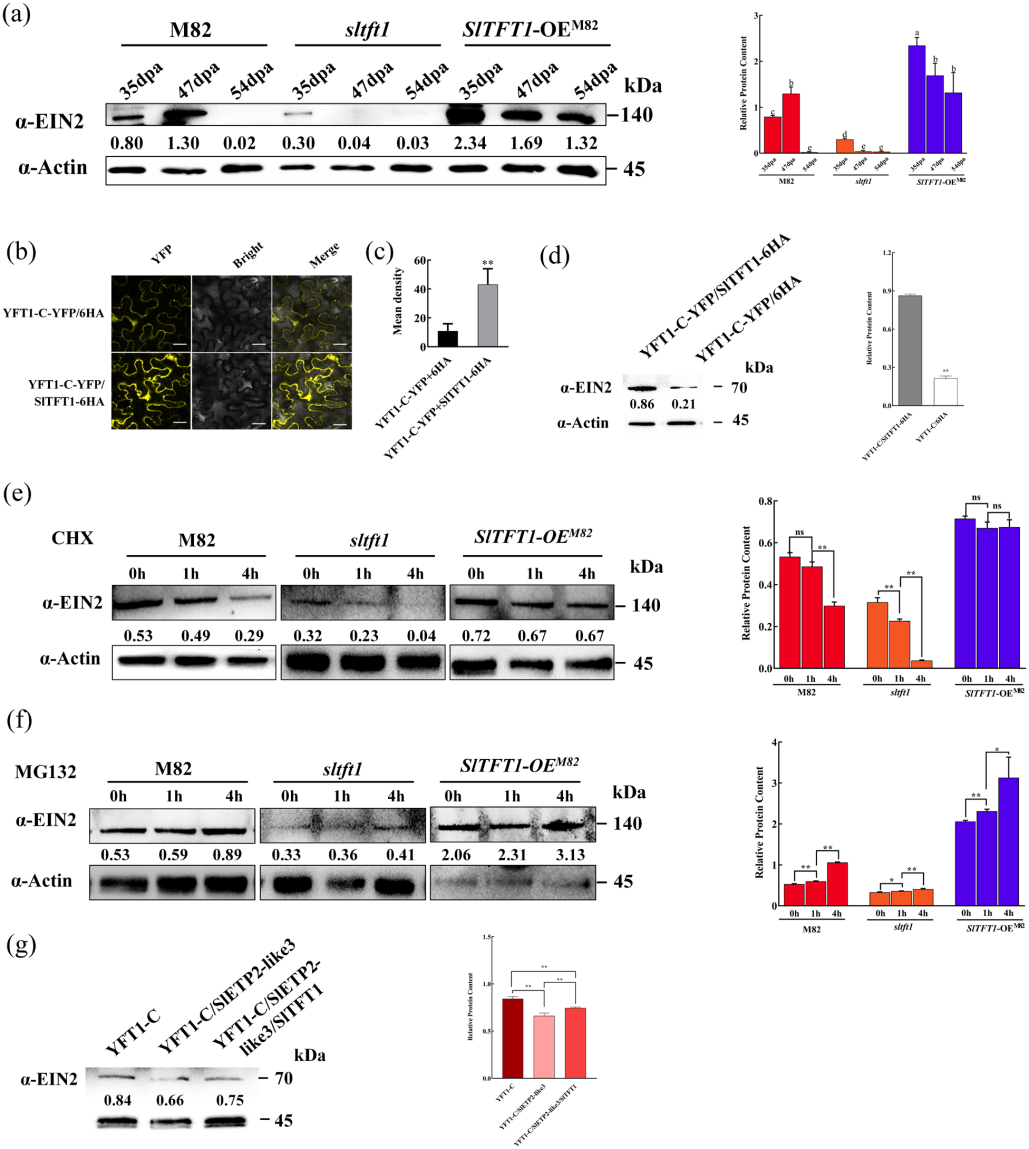
SlTFT1 regulates the YFT1 protein stability. (a) Dynamics changes of the YFT1 protein accumulation in pericarps of different genotype tomatoes. M82, a used as wild‐type tomato; *sltft1*, knockout of *SlTFT1* gene in the M82 background; *SlTFT1‐*OE^M82^, an overexpressed *SlTFT1* tomato line in the M82 background. 35 dpa, 47 dpa and 54 dpa correspond to MG, BR and RR stages at M82. The membranes were probed with anti‐EIN2 (α‐YFT1) and anti‐Actin (α‐Actin) antibodies. The relative signal intensity of each protein normalised to loading control (Actin) is shown below. α‐Actin, a mouse anti‐β‐actin (plant) monoclonal antibody; α‐EIN2, a rabbit anti‐EIN2/YFT1 polyclonal antibody. (b) Effect of SlTFT1 on YFT1‐C stability in tobacco leaves. YFT1‐C‐YFP/SlTFT1‐6HA, two constructs of *YFT1‐C‐YFP* and *SlTFT1‐6HA* were transiently co‐transformed in 6‐week‐old tobacco to determine YFT1‐C abundance. YFT1‐C‐YFP/6HA was used as a control in this experiment. The YFP fluorescence signals were detected with a confocal laser scanning microscope after infection for 36 h. Scale bars, 100 μm. (c) Statistical analyses of fluorescence density of YFT1‐C‐YFP. The fluorescence signal density from each experiment based on panel b was quantified using Image J. ** indicates a significant difference at *p* < 0.01 level determined by Student's *t* test. (d) Quantification of YFT1‐C‐YFP protein by WB. The total proteins were extracted from transformed tobacco leaves and were applied for WB analyses using anti‐EIN2 (α‐YFT1) and anti‐Actin (α‐Actin) antibodies. The relative signal intensity of each protein normalised to loading control (Actin) is shown below. The quantified values were statistically analysed. ** indicates significant difference at *p* < 0.01 level determined by Student's *t* test. (e) Dynamic changes of the YFT1 accumulation in different genotype tomato seedlings treated by CHX. CHX, cycloheximide, a de novo ribosome inhibitor which inhibits protein production in plants. The abundances of the YFT1 protein derived from M82, *sltft1* and *SlTFT1‐*OE^M82^ seedlings were detected using immunoblotting at 0 h, 1 h and 4 h after being treated with 200 μM CHX. The membranes were probed with anti‐EIN2 (α‐YFT1) and anti‐Actin (α‐Actin) antibodies. The relative intensity of each protein was normalised by the loading control (Actin). ** indicates significant difference at *p* < 0.01 level determined by Student's *t* test. (f) Dynamic changes of the YFT1 accumulation in different genotype tomato seedlings treated by MG132. MG132, a 26S proteasome inhibitor which can prevent protein degradation from proteases. The abundances of the YFT1 protein derived from M82, *sltft1* and *SlTFT1‐*OE^M82^ seedlings were detected using immunoblotting at 0 h, 1 h and 4 h after being treated with 50 mM MG132. The membranes were probed with anti‐EIN2 (α‐YFT1) and anti‐Actin (α‐Actin) antibodies. The relative intensity of each protein was normalised by the loading control (Actin). * and ** indicates significant difference at *p* < 0.05 and *p* < 0.01 level determined by Student's *t* test. (g) Regulation YFT1‐C stability by SlTFT1 and SlETP2‐like3. Three plasmids of the *2 × 35S::6HA‐6HA‐YFT1‐C*, *2 × 35S::SlETP2‐like3‐6HA‐YFT1‐C* and *2 × 35S::SlETP2‐like3‐SlTFT1‐YFT1‐C* were respectively transformed into tobacco leaves. After 48 h, the total proteins were extracted to measure YFT1‐C protein abundances by using anti‐EIN2 (α‐YFT1) and anti‐Actin (α‐Actin) antibodies. The relative intensity of each protein was normalised by the loading control (Actin). ** indicates significant difference at *p* < 0.01 level determined by Student's *t* test.

Given that EIN2 is a short‐lived protein with a half‐life of 30 min or less in *Arabidopsis*, and 14‐3‐3 proteins have been reported to increase the protein stability of its interacting protein (Qiao et al. 2009; Yoon and Kieber 2013). We speculated that SlTFT1 could stabilise YFT1 through protein interaction. To test this possibility, we obtained the *SlTFT1* null mutants (*sltft1*) and overexpression lines (*SlTFT1‐OE* ^*M82*^). Further RT‐qPCR results confirmed that the expression of *SlTFT1* is almost undetectable in the *sltft1* fruits but significantly increased in the *SlTFT1‐OE* ^*M28*^ fruits (Figure S3). Western blotting results showed that the YFT1 protein levels in different tomato lines were gradually reduced with fruit ripening. In the wild‐type M82, the peak of endogenous YFT1 protein content occurred at 47 dpa but became almost undetectable at 54 dpa, while overexpression of *SlTFT1* in M82 (*SlTFT1*‐OE^M82^) could significantly increase the total YFT1 level, and the YFT1 protein exhibited a slower degradation rate with fruit ripening (Figure 3a). However, the YFT1 protein became remarkably destabilised and was undetectable after 35 dpa in the *sltft1* line (Figure 3a). At the transcriptional level, *YFT1* is significantly increased in *sltft1* but not significantly changed in *SlTFT1*‐*OE* ^*M82*^ compared to M82 (Figure S3). These results indicated that the decrease or increase of YFT1 protein is not caused by transcriptional expression change but at the post‐transcriptional level. Overall, SlTFT1 could positively regulate the YFT1 expression at the post‐transcriptional level.

To confirm the effect of the SlTFT1 protein on the YFT1‐C stability, we co‐transformed *35S::YFT1‐C‐YFP* and *35S::SlTFT1‐6HA* or *35S::6HA* into tobacco leaves. The intensity of the YFT1‐C‐YFP signal was nearly fourfold in the presence of SlTFT1 compared to the absence of SlTFT1 (Figure 3b,c), indicating that SlTFT1 can stabilise the YFT1‐C protein in plant cells. We also confirmed this result by Western blotting using an anti‐EIN2 antibody (Figure 3d). Taken together, those results indicated that *SlTFT1* could positively regulate the accumulation of the YFT1 protein *in planta*, which may be attributed to the protection of YFT1 from degradation.

To further verify this phenomenon, the 10‐day‐old tomato seedlings with different genotypes were respectively treated with a ribosome inhibitor, cycloheximide (CHX). The total proteins from all treated tomato lines were extracted, and the expression of YFT1 protein was determined by immunoblotting using an anti‐EIN2 antibody. The results showed that the accumulation of YFT1 protein was decreased in all tomato lines with different extents after CHX treatment. After 4‐h CHX treatment, the YFT1 protein contents were reduced to approximately half in M82 and 1/8 *sltft1* lines, whereas there was no significant change in the intensity of YFT1 protein in *SlTFT1*‐OE^M82^ (Figure 3e). However, there was no similar change pattern of YFT1 protein in mock treatment (treating with ddH_2_O), although YFT1 protein content was significantly higher or lower in SlTFT1‐OE^*M82*^ or *sltft1*, than that in M82 (Figure 3e and Figure S4a). These results indicated that YFT1 is also a short‐life protein. To further analyse whether YFT1 protein degradation is mediated by 26S proteasome, we conducted MG132 treatment. YFT1 protein levels became evidently increased in all M82, *sltft1* and *SlTFT1*‐OE^M82^ lines after MG132 treatment compared to DMSO mock treatment (Figure 3f and Figure S4b). We also observed that the YFT1 protein content was significantly higher in *SlTFT1*‐OE^M82^ but significantly lower in *sltft1* than in M82 under both MG132 and DMSO mock treatments (Figure 3f and Figure S4b). These results suggest that the YFT1 protein may be degraded through the 26S proteasome pathway.

It has been reported that the F‐box proteins EIN2 TARGETING PROTEIN 1/2 (ETP1/ETP2) interact with EIN2 and thus lead to degradation of the latter in *Arabidopsis* (Qiao et al. 2009). Three genes (*Solyc02g089310*, *Solyco05g015520* and *Solyc09g091690*) derived from tomato homologous to *AtETP1/2* were highly expressed in tomato fruit based on the websites of a tomato genomic database (https://solgenomics.net/tools/blast/) and Tomato Expression Atlas (http://tea.sgn.cornell.edu) (Figures 1b and 5b). To determine whether the tomato ETP1/ETP2 homologous proteins are involved in YFT1 degradation, we tested their interaction using Y2H assays. Only *Solyc02g089310*, which encodes an AtETP2 homologous protein, interacts with YFT1 (Figure S5a,c). Thereby, we termed it as SlEIN2/YFT1 TARGETING PROTEIN 2 like 3 in tomato (SlETP2‐like3). We further co‐transformed *YFT1* and empty vector plasmid, *YFT1* and *SlETP2‐like3*, and *YFT1* and *SlETP2‐like3*/SlTFT1 into tobacco leaves. The WB data indicated that SlETP2‐like3 could mediate YFT1 degradation and co‐expression of SlTFT1 could partially recover YFT1 from degradation (Figure 3g). These results suggest that SlTFT1 can bind and stabilise YFT1 from degradation mediated by SlETP2‐like3 and thus can facilitate YFT1 accumulation in tomato.

### 2.4 SlTFT1 Promotes Ethylene Emission and Ethylene Signalling Transduction

**FIGURE 4:**
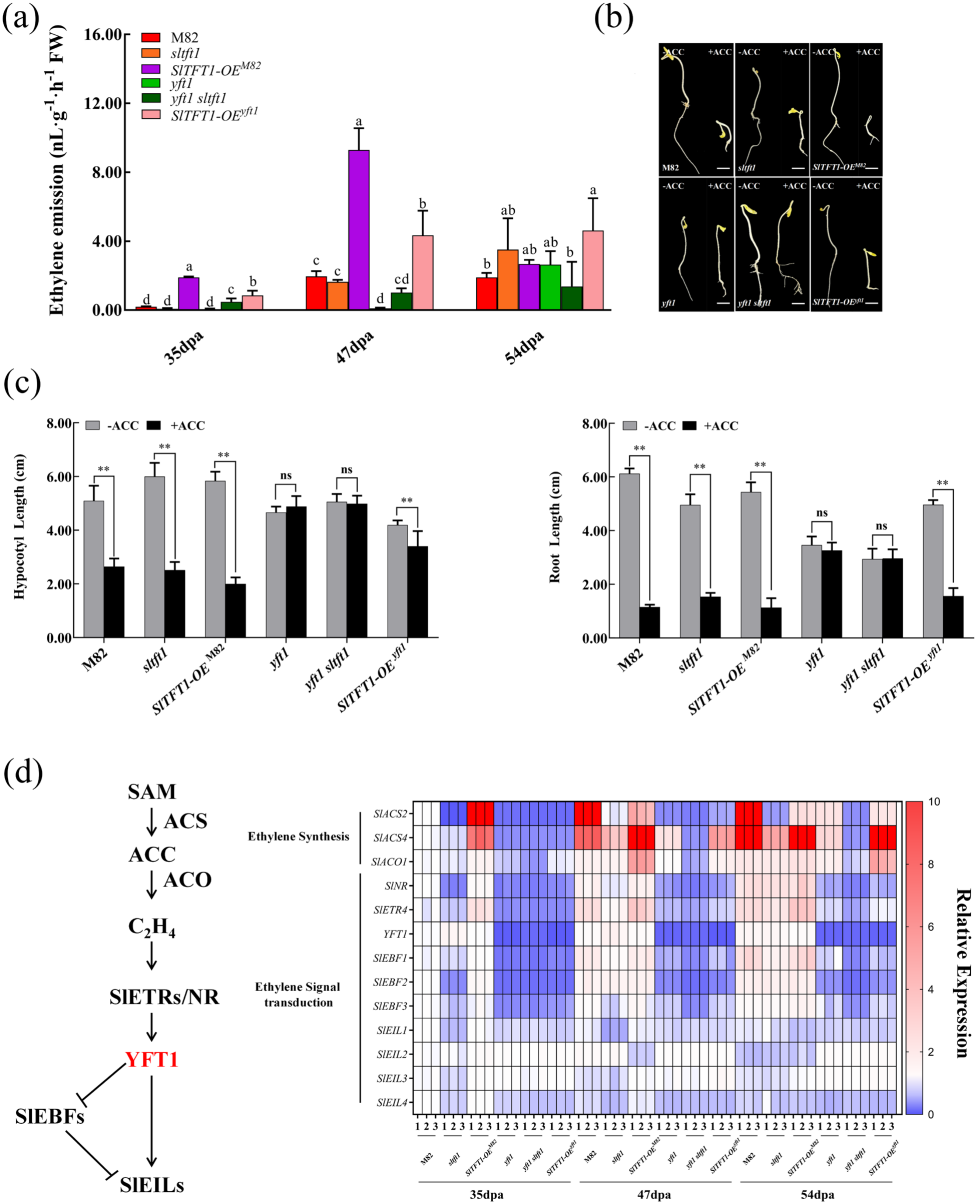
*SlTFT1* promotes ethylene synthesis and signal transduction in tomatoes. (a) Ethylene emission of the different genetic type tomatoes. dpa, days post‐anthesis; M82, a wild‐type tomato; *sltft1* and *yft1 sltft1*, *SlTFT1* knockout tomato lines derived from M82 and *yft1* lines created from backgrounds by CRISPR‐cas9 approach; *SlTFT1*‐OE^M82^ and *SlTFT1*‐OE^*yft1*^, overexpression of *SlTFT1* in M82 and *yft1* lines; *yft1*, a yellow‐fruited tomato 1 mutant derived from M82 by fast‐neutron irradiation (accession *n3122*). 35 dpa, 47 dpa and 54 dpa represent mature green (MG), breaker (BR) and red ripe (RR) stages in M82. FW, fresh weight. The lowercase letters indicate statistical significance at the *p* < 0.05 level determined by Duncan's test (*n* = 3). (b) Triple response assay. ACC, 1‐aminocyclopropane carboxylic acid. The tomato seedlings of different genotypes were grown on 1/2 MS solid media supplied with 10‐mM ACC (+ACC) or 0‐mM (−ACC) in the dark condition at 25°C for 5 days were photographed by Canon Eos 800D. The growth and development of tomato seedlings were observed and recorded. Scale bars, 1 cm. (c) Effect of ACC on hypocotyl and root growth of different genotype tomato seedlings. The lengths of hypocotyls and roots of tomato seedlings treated with 10 mM and 0 mM ACC were measured and statistically analysed. ** indicates significant difference at *p* < 0.01 level determined by Student's *t* test; ns, Non‐significant. (d) Schematic and expression heat map of genes that are involved in ethylene synthesis and signalling transduction in different genotype tomatoes. *SlACO1*, *ACC OXIDASE 1*; *SlETR4*, *ETHYLENE RECEPTOR 4*; *SlNR*, *NEVER RIPE*; *SlEIN2*, *ETHYLENE‐INSENSITIVE 2* (*YFT1*); *SlEBF1/2/3*, *EIN3‐BINDING F‐BOX PROTEIN 1/2/3*; *SlEIL1/2/3/4*, *ETHYLENE‐INSENSITIVE3‐Like 1/2/3/4 in Solanum lycopersicum*; *SlACS2/4*, *1‐AMINOCYCLOPROPANE‐1‐CARBOXYLATE SYNTHASE 2/4*.

To reveal the physiological functions of SlTFT1, we measured the ethylene emissions of these tomato lines at different fruit stages. The results showed that ethylene production was generally increased with fruit mature development in all tomato lines (Figure 4a). Ethylene production peaks at 47 dpa in *SlTFT1‐OE* ^*M82*^ and wild‐type, but peaks at 54 dpa in *sltft1*, *yft1*, *ytf1sltft1* and *SlTFT1‐OE* ^*yft1*^. Although the ethylene production peaks at 47 dpa in both *SlTFT1‐OE* ^*M82*^ and wild‐type, the ethylene production in *SlTFT1‐OE* ^*M82*^ at 35 dpa is almost the same as wild‐type at 47 dpa (Figure 4a). These results suggest that the system‐2 ethylene biosynthesis has been delayed in *yft1* and *sltft1* mutants but has been advanced in *SlTFT1‐OE* ^*M82*^. The ethylene production is significantly higher in *SlTFT1‐OE* ^*M82*^ compared to M82, and overexpression of *SlTFT1* in *yft1* mutant (*SlTFT1‐OE* ^*yft1*^) can partially recover the ethylene deficiency phenotype (Figure 4a). The *yft1* is a *YFT1* lesion mutant (Gao et al. 2016). Overexpression of *SlTFT1* into *yft1* mutant may protect YFT1 from degradation, which caused the increase of ethylene production. We also found that the ethylene production of *SlTFT1‐OE* ^*yft1*^ is significantly lower than *SlTFT1‐OE* ^*M82*^ at 35 and 47 dpa, suggesting that the function of SlTFT1 relies on the existence of YFT1 (Figure 4a). These results demonstrated that SlTFT1 can positively regulate the ethylene emission and has an additive effect with the YFT1 protein.

YFT1 is a core component in the ethylene signalling pathway, and SlTFT1 could protect it from degradation. To investigate whether SlTFT1 is also involved in ethylene signalling, we conducted the triple response assay. Tomato seedlings with different genetic backgrounds were treated with 10 μM 1‐aminocyclopropane carboxylic acid (ACC). Unlike the *yft1* seedling, the *sltft1* and *SlTFT1‐OE* ^*M82*^ seedlings were sensitive to ethylene's inhibition of hypocotyl elongation and root growth (Figure 4b,c). The sensitivity of ethylene in *sltft1* was abolished when the mutation of *yft1* was introduced, suggesting that YFT1 functions downstream of SlTFT1 (Figure 4b,c). Moreover, overexpression of *SlTFT1* in the *yft1* mutant could partially recover its ethylene‐insensitive phenotype, which may be caused by stabilisation of YFT1, suggesting that SlTFT1 is a positive regulator in the ethylene signalling pathway (Figure 4b,c). These results indicated that SlTFT1 positively regulates the ethylene signal pathway and may work through stabilising YFT1 protein.

To reveal the molecular mechanisms of how SlTFT1 regulates ethylene synthesis and signal transduction, we detected the expression of key ethylene synthesis enzyme genes and ethylene signal and delivery genes in different genotypes during tomato fruit ripening.

In the ethylene synthesis pathway, the expression profiling of *1‐AMINOCYCLOPROPANE‐1‐CARBOXYLATE SYNTHASE 2/4* (*SlACS2/4*) and*1‐AMINOCYCLOPROPANE‐1‐CARBOXYLATE OXIDASE 1* (*SlACO1*) were increased in all tomato lines along with fruit mature development (Figure 4d). The *SlACS2* transcript peaks at 35 dpa in *SlTFT1‐OE* ^M82^ but peaks at 47 dpa in M82 and at 54 dpa in *sltft1*, *yft1* and *SlTFT1‐OE* ^*yft1*^. The transcripts of *SlACS2* and *SlACO1* peak at 47 dpa in *SlTFT1‐OE* ^M82^ but peak at 54 dpa in M82, *sltft1*, *yft1* and *SlTFT1‐OE* ^*yft1*^. However, the expressions of these three genes were always at a low level or almost undetectable in the *yft1 sltft1* line during the whole fruit maturation period (Figure 4d). We also found that the expression levels of *SlACS2/2* and *SlACO1* were significantly higher in *SlTFT1* overexpression tomato lines (*SlTFT1‐OE* ^M82/*yft1*^) than those in their background tomatoes (M82/*yft1*), but were significantly lower in *SlTFT1* null mutants (sltft1 and *sltft1 yft1*) (Figure 4d). These results suggest that *SlTFT1* can positively regulate the *SlACS2/2* and *SlACO1* expressions and accelerate the occurrence of the respiratory climacteric timepoint in a YFT1‐dependent manner. The changing trends of *SlACS2/4* and *SlACO1* expressions are consistent with ethylene emission (Figure 4a,d). These results provide molecular evidence that *SlTFT1* can promote ethylene emission during fruit ripening.

In the ethylene signalling pathway, expression levels of ethylene receptor‐associated genes such as *ETHYLENE RECEPTOR 4* (*SlETR4*), *NEVER RIPE* (*SlNR*) and *EIN3‐BINDING F‐BOX PROTEIN1/2/3* (*SlEBF1/2/3*) were significantly suppressed in different tomato lines with *yft1* background compared to the lines with M82 background (Figure 4d). Moreover, *SlETR4*, *SlNR* and *SlEBF1/2/3* were further suppressed in *sltft1* or *yft1 sltft1* and were significantly induced in *SlTFT1*‐OE^M82^ and *SlTFT1*‐OE^*yft1*^ lines compared with M82 or *yft1* lines except for *SlEBF1/2/3* in both *SlTFT1*‐OE^M82^ and *SlTFT1*‐OE^*yft1*^ lines (Figure 4d). These results suggest that the expressions of *SlETR4*, *SlNR* and *SlEBF1/2/3* were induced by ethylene and were positively regulated by *SlTFT1*.

The ETHYLENE‐INSENSITIVE3‐Likes (SlEILs), a class of transcription factors, are essential components of the ethylene signalling pathway in the nucleus. The expression levels of *SlEIL1/2/3/4* were slightly decreased with fruit ripening in both M82 and *SlTFT1*‐OE^M82^; however, the expression levels of *SlEIL1/3/4* were severely suppressed in *sltft1* compared to M82 and *SlTFT1*‐OE^M82^ at 35 dpa and 47 dpa, but not at 54 dpa. However, *SlEIL* expressions kept relatively steady levels in all *yft1* background tomatoes throughout the fruit ripening period, except that *SlEIL3* in the *SlTFT1*‐OE^*yft1*^ line was significantly up‐regulated compared with that in the *yft1* line at 47 dpa. However, the expressions of *SlEIL1/2/3/4* showed no difference among *yft1 sltft1*, *yft1* and *SlTFT1*‐OE^*yft1*^. Nevertheless, the expression levels of *SlEIL1/4* were higher in M82 background tomatoes compared with those in *yft1* background; however, *SlEIL2/3* presented an opposite expression pattern in these lines (Figure 4d). Those results implied that *SlTFT1* positively regulates ethylene signal transduction through regulating the relevant genes and has an additive effect with *YFT1*.

**FIGURE 5:**
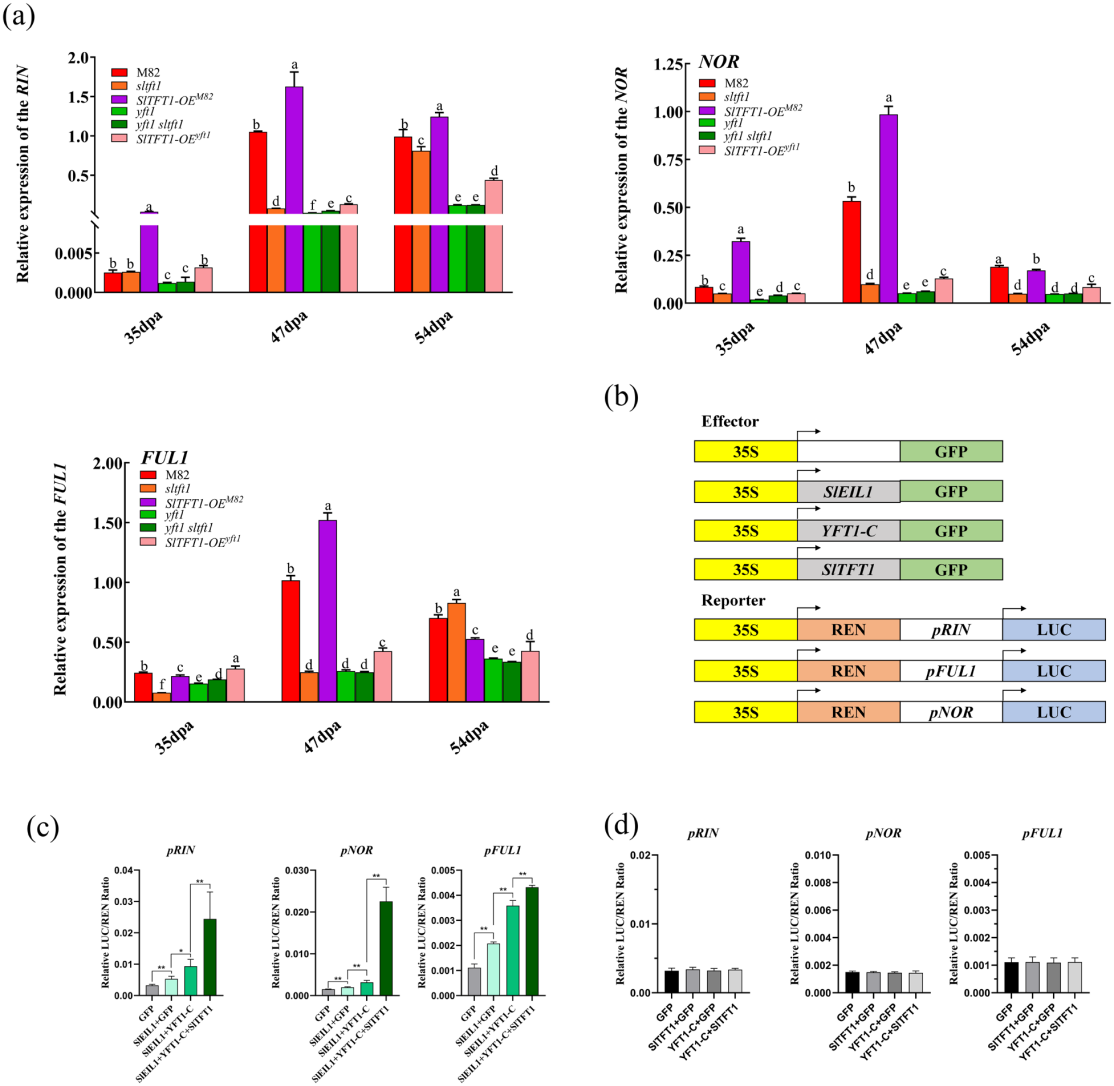
*Effect of SlTFT1 on t*ranscription of *RIN*, *NOR* and *FUL1* in different tomato lines. (a) Expression of *RIN*, *NOR* and *FUL1* in different tomato lines. *FUL1*, *FRUITFULL 1* in *Solanum lycopersicum* ; *NOR*, *NON‐RIPENING*; *RIN*, *RIPENING‐INHIBITOR*. Error bars indicate standard deviation (SD, *n* = 3). Lowercase letters indicate statistical significance at the *p* < 0.05 level by the Duncan test. (b) Diagrams of effector and reporter constructs for dual‐LUC assays. The *SlEIL1*, *SlTFT1* and *YFT1‐C* were constructed in the downstream of the 35S promoter and were used as effectors. An empty 35S effector construct was used as a negative control. The *LUC* gene was, respectively, inserted in the downstream of the promoters of the *RIN*, *NOR* and *FUL1* genes (*pRIN*, *pNOR* and *pFUL1*). LUC, firefly luciferase; REN, renilla luciferase. (c) Effect of *SlTFT1* on luciferase activities controlled by *pRIN*, *pNOR* and *pFUL1*. The luciferase activities from each combination were quantitatively measured and were statistically analysed. Error bars indicate SD (*n* = 3). * or ** indicates statistical significance at *p* < 0.05 or *p* < 0.01 levels by Student's test. (d) Effects of YFT1‐C and SlTFT1 on luciferase activities controlled by *pRIN*, *pNOR* and *pFUL1* in a SlEIL1‐dependent pattern. The luciferase activities from each combination were quantitatively measured and were statistically analysed. Error bars indicate SD (*n* = 3). * or ** indicates statistical significance at *p* < 0.05 or *p* < 0.01 levels by Student's *t* test.

We also validated that SlTFT1 could promote ethylene signalling transduction through the protection of YFT1 protein from degradation during tomato fruit ripening; however, how SlTFT1 affects the expression of the ethylene downstream genes in this process still remains unknown. It is reported that ethylene induces tomato fruit ripening through regulating the expression of the downstream transcription factor genes like *RIN*, *NOR* and *FUL1* by SlEIL proteins (Huang et al. 2022). Our results showed that the expression levels of *RIN*, *NOR* and *FUL1* were significantly suppressed in *sltft1* during fruit ripening compared with M82 but were induced in *SlTFT1*‐OE^M82^. The expression levels of *RIN*, *NOR* and *FUL1* were evidently elevated in *SlTFT1*‐OE^*yft1*^ compared with *yft1; however*, their expressions did not show a difference amongn *yft1*, *sltft1* and *yft1* tomato lines (Figure 5a).

To further confirm how SlTFT1 affects the expression of *RIN*, *NOR* and *FUL1* genes, we performed dual‐luciferase reporter (DLR) assays in tobacco leaves. The results indicated that the expression levels of luciferase driven by the *RIN*, *NOR* and *FUL1* promoters (*pRIN*, *pNOR* and *pFUL1*) were significantly increased in the presence of SlEIL1. Moreover, the transcriptions of *pRIN‐LUC*, *pNOR‐LUC* and *pFUL1‐LUC* driven by SlEIL1 were significantly enhanced by co‐expression of *YFT1‐C* or *YFT1‐C/SlTFT1* (Figure 5b,c). However, SlTFT1, YFT1‐C or TFT1/YFT1‐C without SlEIL1 could not activate expression of *pRIN‐LUC*, *pNOR‐LUC* and *pFUL1‐LUC* (Figure 5d). These results implied that the SlTFT1‐YFT1‐C complex could activate expressions of the downstream genes in a SlEILs‐dependent manner.

### 2.5 SlTFT1 Modulates Fruit Ripening, Chromoplast Development and Carotenoid Accumulation in Tomato Fruits

**FIGURE 6:**
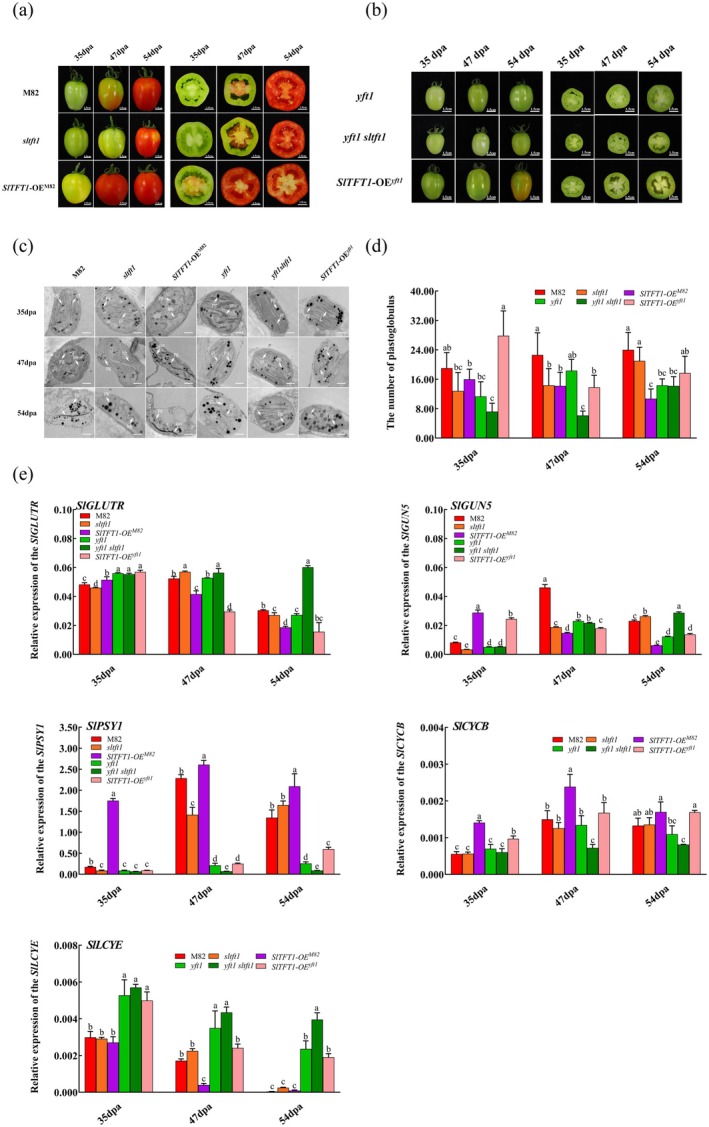
Effect of *SlTFT1* on fruit ripening, chromoplast development and carotenoid accumulation in tomato fruits. (a) Fruit colouring of different genotype tomatoes in M82 backgrounds at different developmental stages. The representative whole fruits and the fruit cross‐sections were photographed at 35 dpa, 47 dpa and 54 dpa, which correspond to MG, BR and RR stages. M82 is a wild‐type tomato line. Scale bars = 1.5 cm. (b) Fruit colouring of different genotype tomatoes in *yft1* background at different developmental stages. The representative whole fruits and the fruit cross‐sections were photographed at 35 dpa, 47 dpa and 54 dpa, which correspond to MG, BR and RR stages. *yft1* is a *YFT1* mutant tomato line. Scale bars = 1.5 cm. (c) Effect of SlTFT1 on the development of chromoplasts within pericarp cells in different genotype tomato lines. The tomato fruit pericarp cells from different genotypes were observed under a transmission electron microscope (TEM), and the representative photos were demonstrated. ccr, Carotenoid crystalloids; gr, grana; lth, long linear thylakoid membrane structure; pg, plastoglobules. Scale bars = 0.5 μm. (d) Dynamic changes of plastoglobuli numbers in different genotype tomato fruits. Data obtained from six TEM fields (*n* = 6). Small letters indicate the statistical significance determined by a Duncan's test at *p* < 0.05 level. (e) Expression of genes which are involved in the chromoplast development and carotenoid synthesis in different tomato lines. *SlCYCB*, *CHROMOPLAST SPECIFIC LYCOPENE β‐CYCLASE*; *SlGLUTR*, *GLUTAMYL‐tRNA REDUCTASE*; *SlGUN5*, *GENOMES UNCOUPLED'5*; *SlLCYE*, *LYCOPENE ε‐CYCLASE* in *Solanum lycopersicum* ; *SlPSY1*, *PHYTOENE SYNTHASE 1*. Error bars indicate SD (*n* = 3). The lowercase letters indicate statistical significance at *p* < 0.05 level by Duncan's test.

Under normal conditions, the mature green (MG), breaker (BR) and red ripe (RR) stages of the wild‐type M82 fruits are respectively occurring at 35 dpa, 47 dpa and 54 dpa. In our previous report, we found that *yft1* mutant fruits have not reached the BR stage until 54 dpa due to a 573‐bp insertion at the regulatory region of the *YFT1* promoter (Zhao et al. 2020). Here, we found that *SlTFT1* governs fruit coloration during tomato fruit ripening (Figure 6a). Fruits of the *SlTFT1‐OE* ^*M82*^ tomato line reached the BR and RR stages at 35 and 47 dpa, which is earlier than the wild‐type (Figure 6a), but those in the *sltft1* tomato line began to ripen at 47 dpa, which is slightly later than M82 (Figure 6a). Although overexpression of *SlTFT1* (*SlTFT1*‐OE^*yft1*^) can accelerate fruit ripening in the *yft1* background, its MG and BR stages came at 47 dpa and 54 dpa. The MG stages of *yft1* and *yft1 sltft1* tomatoes were delayed to 54 dpa (Figure 6b). The fruit ripening process of the *SlTFT1*‐OE^*yft1*^ is earlier than that of *yft1* but later than that of *SlTFT1*‐OE^*M82*^. These results demonstrate that *SlTFT1* positively modulates tomato fruit ripening and depends on *YFT1*.

Along with fruit maturing, the membrane structures of chloroplasts in tomato fruit pericarp cells are gradually disintegrated and are then converted into chromoplasts where carotenoids are synthesised to form plastoglobuli or crystalloids (Gao et al. 2016; Zhao et al. 2023). Here, our electron microscopy data indicated that the membrane envelope, thylakoid lamellae and grana verges in chloroplasts were visible in fruit pericarp cells of both M82 and *sltft1* tomatoes at 35 dpa. However, the thylakoid lamellae and grana verges began to be indistinct, the envelopes were well developed and the sizes of the formed plastoglobuli increased to some extent in *SlTFT1*‐OE^M82^ line at the same time point (Figure 6c). In both M82 and *sltft1* tomato pericarp cells, the membrane envelopes were still intact and were clearly visible, and the thylakoid lamellae and grana structure were also distinguishable and began to convert into a long linear thylakoid (*lth*) structure at 47 dpa, whereas in the chromoplasts of *SlTFT1*‐OE^M82^ fruit pericarp cells, all the membrane systems such as membrane envelope, thylakoid and grana began to collapse and were replaced by carotenoid crystalloids (ccr)‐deposited generous carotenoids at 47 dpa (Figure 6c). The membrane structures were gradually converted to lth structures in M82 and *sltft1* at 54 dpa, and the *ccr* structures changed into black stripe shape at this stage. However, in the *SlTFT1*‐OE^M82^ line, the membrane structures were completely disappeared and the plastoglobuli number decreased, those turned into *ccr* structure with thick branch shape (Figure 6c,d). The membrane envelope and thylakoid membrane were clearly visible within pericarp cells in both *yft1* and *yft1 sltft1* tomato lines from 35 dpa to 54 dpa, whereas the thylakoid membranes became vague in *SlTFT1*‐OE^*yft1*^ at the same period. However, the ccr structures were only observed in chromoplasts in M82 but not in *yft1* background tomato fruits, and the *SlTFT1*‐OE^*yft1*^ fruits had significantly more plastoglobuli than the *yft1* and *yft1 sltft1* fruits. Moreover, the *yft1* had more plastoglobuli than *yft1 sltft1* (Figure 6c,d). These results suggest that the *SlTFT1* promotes the chromoplast development process in tomato fruits in a YFT1‐dependent manner.

With fruit ripening and chromoplast formation, the tomato fruit colour begins to turn from green to red with the synthesis of lycopene. At 35 dpa, lycopene was not measurable in all tomato lines. At 47 dpa and 54 dpa, the lycopene content in *SlTFT1*‐OE^M82^ fruits was significantly higher than those in M82 and *sltft1* mutant. However, in the *yft1* background tomato lines, the lycopene accumulation was seriously suppressed and even could not be detectable until 54 dpa (Table 1). At that time, the β‐carotene also accumulates with the fruit mature development. In M82 background tomatoes, the β‐carotene contents were higher in *SlTFT1*‐OE^M82^ than in M82 and *sltft1* lines at 35 dpa and 47 dpa. However, the β‐carotene contents decreased with fruit ripening in tomato lines of the *yft1* background lines, except for *SlTFT1*‐OE^*yft1*^. The β‐carotene content in *yft1 sltft1* was significantly higher than that in *yft1* and *SlTFT1*‐OE^*yft1*^ during early fruit ripening (35 dpa and 47 dpa). The β‐carotene content in the *SlTFT1*‐OE^*yft1*^ line was significantly higher than that in *yft1* and *yft1 sltft1* tomato lines in late fruit ripening (54 dpa) (Table 1). The β‐carotene accumulations in *yft1* background tomatoes were less than those in M82 background tomatoes at 47 dpa and 54 dpa, but the significant difference was not detected between M82 and *yft1* at 35 dpa (Table 1). The lutein content was lower in M82 background tomato lines than that in *yft1* background tomatoes. Lutein accumulation decreased in *SlTFT1*‐OE^M82^ with fruit ripening, but the obvious difference was not detected between M82 and *sltft1* lines throughout the fruit maturing period. However, the lutein content decreased in *yft1* and *yft1 sltft1* lines with fruit ripening; however, it slightly increased in the *SlTFT1*‐OE^*yft1*^ line. The lutein content in *SlTFT1*‐OE^*yft1*^ was significantly higher than that in *yft1* at 54 dpa (Table 1). The α‐carotene content was too low to be detected in both M82 and *yft1* lines (Table 1).

**TABLE 1:** The carotenoid contents of tomato fruits in different genetic backgrounds (μg·g^−1^ FW).

	Lycopene	α‐carotene	β‐carotene	Lutein	Total carotenoids	Lycopene/β‐carotene
35 dpa						
M82	—	—	2.61 ± 0.28^E^	5.00 ± 0.13^G^	7.61 ± 0.41	—
*sltft1*	—	—	1.21 ± 0.27^F^	3.31 ± 0.16^I^	4.52 ± 0.43	—
*SlTFT1*‐OE^M82^	—	—	8.34 ± 2.41^B^	12.19 ± 1.76^A^	20.53 ± 4.17	—
*yft1*	—	—	4.44 ± 0.45^D^	8.29 ± 0.13^C^	12.73 ± 0.58	—
*yft1 sltft1*	—	0.70 ± 0.52^B^	6.37 ± 0.11^C^	10.37 ± 0.04^B^	17.44 ± 0.67	—
*SlTFT1*‐OE^*yft1*^	—	—	2.55 ± 0.22^E^	5.88 ± 0.12^EF^	8.43 ± 0.34	—
47 dpa						
M82	16.68 ± 0.05^C^	—	4.77 ± 0.43^D^	5.97 ± 0.04^EF^	27.42 ± 0.52	3.49
*sltft1*	9.90 ± 1.30^C^	—	4.50 ± 0.76^D^	3.44 ± 0.05^I^	17.84 ± 2.11	2.2
*SlTFT1*‐OE^M82^	115.80 ± 5.61^B^	1.83 ± 1.22^B^	9.97 ± 0.33^A^	6.47 ± 0.52^E^	134.07 ± 7.68	11.61
*yft1*	—	—	2.93 ± 0.07^E^	5.52 ± 0.20^FG^	8.45 ± 0.27	—
*yft1sltft1*	—	0.23 ± 0.14^B^	4.44 ± 0.16^D^	7.27 ± 0.16^D^	11.94 ± 0.46	—
*SlTFT1*‐OE^*yft1*^	—	—	3.15 ± 0.17^DE^	5.91 ± 0.05^EF^	9.06 ± 0.22	—
54 dpa						
M82	114.74 ± 13.20^B^	0.90 ± 0.30^B^	8.80 ± 0.11^B^	4.66 ± 0.17^GH^	129.1 ± 13.78	13.04
*sltft1*	110.93 ± 2.48^B^	—	7.62 ± 0.15^B^	6.04 ± 0.09^EF^	124.59 ± 2.72	14.56
*SlTFT1*‐OE^M82^	162.64 ± 19.26^A^	4.13 ± 1.54^A^	8.74 ± 0.40^B^	3.97 ± 0.34^HI^	179.48 ± 21.54	18.61
*yft1*	—	—	2.64 ± 0.10^E^	5.38 ± 0.22^FG^	8.02 ± 0.32	—
*yft1 sltft1*	—	0.27 ± 0.19^B^	4.20 ± 0.04^DE^	7.94 ± 0.11^CD^	12.41 ± 0.34	—
*SlTFT1*‐OE^*yft1*^	—	—	6.19 ± 0.13^C^	7.46 ± 0.03^CD^	13.65 ± 0.16	—

*Note:* *sltft1* and *yft1 sltft1* indicate that *SlTFT1* knock‐out tomato lines were, respectively, created from M82 and *yft1* backgrounds using CRISPR‐cas9; *SlTFT1*‐OE^M82^ and *SlTFT1*‐OE^*yft1*^ indicate the overexpressed *SlTFT1* in M82 and *yft1* backgrounds through *A.tumefaciens*‐mediated transgene using plasmid 2 × 35S::*SlTFT1‐CDS*. The values in the table are mean ± SD (*n* = 3). The capital letters indicate statistical significance at *p* < 0.01 as determined by Duncan's test.

Abbreviations: FW, fresh weight; M82, a cultivated tomato variety, which was used as wild‐type in the present study; *yft1*, a yellow‐fruited tomato mutant was created from M82 by fast‐neutron irradiation M82 (original accession *n3122*).

In M82 background tomato lines, the total carotenoid contents were increased along with fruit ripening, and it ranked from the highest to the lowest in *SlTFT1*‐OE^M82^, M82 and *sltft1* tomato lines (Table 1). M82 background tomatoes had significantly higher total carotenoid contents than *yft1* genotype tomatoes at 47 dpa and 54 dpa, but not at 35 dpa. In *yft1* background tomatoes, *yft1* and *yft1 sltft1* tomatoes had slightly decreased total carotenoids; however, *SlTFT1*‐OE^*yft1*^ had increased total carotenoids with fruit development (Table 1).

The ratio of lycopene to β‐carotene determines fruit coloration in tomato. This ratio was significantly higher in *SlTFT1*‐OE^M82^ than that in M82 and *sltft1* tomatoes, and the ratios ranged from 18.61 (*SlTFT1*‐OE^M82^) to 13.04 (M82) (Table 1). These results indicate that *SlTFT1* positively regulates the chromoplast development process, carotenoid accumulation and fruit coloration.

To reveal the molecular mechanism of chromoplast development and carotenoids synthesis regulated by *SlTFT1*, herein we dissected transcriptional expressions of some key genes which are involved in chloroplast development and chromoplast formation/carotenoids synthesis. *SlGLUTR* (***GL**UTAMYL‐**T**RNA **R**EDUCTASE*) and *SlGUN5* (***G**ENOMES **UN**COUPLED* ***5***) encode two key enzymes in chlorophyll biosynthesis (Sinha et al. 2022; Xu et al. 2025; Zhang et al. 2020). The RT‐qPCR results showed that the expression levels of both *SlGLUTR* and *SlGUN5* were increased in *SlTFT1* knockout lines (*sltft1* and *yft1 sltft1*) with fruit ripening but decreased in the overexpressed *SlTFT1* lines (*SlTFT1*‐OE^M82^ and *SlTFT1*‐OE^*yft1*^) (Figure 6e).

As the onset of tomato fruit ripens, an important sign is the chromoplast formation and carotenoids deposition. It has been reported that *SlLCYE* (*LYCOPENE ε‐CYCLASE*) in the chloroplast and *SlPSY1* (*PHYTOENE SYNTHASE*) and *SlCYCB* (*LYCOPENE β‐CYCLASE*) in the chromoplast were specifically expressed (Li et al. 2024; Zhao et al. 2023).

The expression levels of both *SlPSY1* and *SlCYCB* were increased with fruit ripening. In the overexpressed *SlTFT1* lines (*SlTFT1*‐OE^M82^ or *SlTFT1*‐OE^*yft1*^), we found that the expressions of both *SlPSY1* and *SlCYCB* were significantly elevated and advanced compared to those in M82 or *yft1* lines. On the contrary, in the *SlTFT1* knockout lines (*sltft1* or *yft1 sltft1*), the expressions of *SlPSY1* and *SlCYCB* were severely suppressed compared to M82 and *yft1*, and their expression peaks were delayed (Figure 6e). On the other hand, the expression of the *SlLCYE* gene was decreased with fruit ripening, and its expression level in M82 background lines was significantly lower than that in *yft1* background tomatoes. The *SlLCYE* expression was significantly down‐regulated in the *SlTFT1*‐OE^M82^ and *SlTFT1*‐OE^*yft1*^ lines compared to M82 and *yft1* lines at 47 dpa but was markedly up‐regulated in *sltft1* and *yft1 sltft1* lines compared to M82 and *yft1* lines at 47 dpa and 54 dpa (Figure 6e).

Taken together, these results from analyses of chromoplast ultrastructure, carotenoid accumulations and expressions of specific genes implied that *SlTFT1* and *YFT1* synergistically regulate chromoplast development and accumulation of the carotenoids during tomato fruit ripening.

## 3 Discussion

The fruit ripening of tomato was triggered and regulated by the gaseous plant hormone ethylene. EIN2 acts as a key positive regulator in this process; however, the underlying mechanisms, especially the delicate regulation of EIN2 protein content and haemostasis, remain largely unclear. In this study, we found a new SlEIN2/YFT1‐interacting tomato 14‐3‐3 protein, SlTFT1, which positively regulates ethylene signalling output and fruit ripening of tomato through interacting with and stabilising YFT1 protein.

The gaseous plant hormone, ethylene, plays pleiotropic roles in plant growth, development and stress responses (Shao et al. 2024; Wang et al. 2021). Especially in the typical climacteric tomato fruits, the fruit ripening process and quality formation are tightly associated with ethylene through signalling delivery (Huang et al. 2022; Zhao et al. 2020), in which EIN2 is a central and unique signal molecule residing at a critical hub‐node in the ethylene signalling pathway and directly interacts with the ethylene receptors (ETRs) at the ER membrane (Bisson et al. 2009). We also demonstrated that *SlEIN2*/*YFT1* and *SlTFT1* can positively regulate the transcriptional expression of the genes associated with ethylene synthesis (*SlACS2/4* and *SlACO1*), signalling (*SlNR*, *SlETR4*, *SlCTR1*, *SlEILs*, *SlERF6, SlEBFs, RIN, NOR and FUL1*) and carotenoid synthesis (*SlPSY1*, *SlLCYE* and *SlCYCB*) (Zhao, Li, Fan, et al. 2021; Zhao et al. 2023; Figures 4d, 5a and 6e); these results implied that *SlTFT1* participates in the processes of ethylene signal transduction and fruit ripening through regulating those genes in a YFT1‐dependent manner.

In fact, EIN2 delivers ethylene signalling through translocating to the P‐body and nucleus (Ju et al. 2012; Li et al. 2015; Merchante et al. 2015; Qiao et al. 2012) and chromatin remodelling by elevating histone H3K14 and H3K23 acetylation (Shao et al. 2024; Zhang et al. 2017) to activate the expression of the downstream genes to respond to ethylene. However, as a short‐lived protein, how EIN2 could continuously deliver the ethylene signal still remains enigmatic (Qiao et al. 2009). Ethylene does not induce *EIN2* expression at the transcriptional level; however, the regulation of EIN2 protein content is an important and interesting question (Alonso et al. 1999). Our previously reported that two ethylene‐independent transcription factors WRKY32 and EMB1444‐like can positively regulate *YFT1* expression at the transcriptional level (Zhao, Li, Fan, et al. 2021; Zhao et al. 2023). However, how YFT1 protein is regulated during tomato fruit ripening remains largely unknown. SPINDLY (SlSPY) protein interacts with and stabilises SlEIN2 probably through O‐glycans modification (Xu et al. 2023). Here, biochemical and molecular evidence in this study also uncovered that SlTFT1 interacts with and stabilises YFT1 protein through inhibiting the degradation role of SlETP2‐like3 in tomato (Figure 3 and Figure S5c). In *Arabidopsis*, the highly conserved two F‐box proteins, ETP1/2, mediate the degradation of the interacting protein EIN2 through the ubiquitin/26S proteasome system (Qiao et al. 2009). This mode of action for SlETP2‐like3 is similar to its homologous proteins in *Arabidopsis*. Although SlETP1‐like, SlETP2‐like2 and SlETP2‐like3, which encode homologous proteins to the *Arabidopsis* ETP1/2 proteins (Figure S5), are highly expressed in tomato fruit ripening compared with the other six SlETP2‐like proteins (Figure S5b), whereas only SlETP2‐like3 could interact with SlEIN2/YFT1 (Figure S5c). These results suggest that SlETP2‐like3 probably participates in the regulatory process of the YFT1 protein homeostasis during tomato fruit ripening.

The 14‐3‐3 protein was originally found in bovine brain homogenate (Aitken 2006; Ichimura et al. 1988; Moore and Perez 1967) and is widely distributed in eukaryotic organisms (Ma et al. 2023; Ormancey et al. 2017; Zhao, Li, and Li 2021). In addition to almost all aspects of the plant life cycle, 14‐3‐3 proteins also conspicuously participate in the processes of phytohormone signalling, biosynthesis and transport (Camoni et al. 2018). For example, 14‐3‐3 proteins function in Brassinosteroid (BR) signalling via interacting with the BR receptors such as brassinosteroid‐insensitive1 (BRI1) and interact with ABA (abscisic acid)‐responsive element binding factor 3 (ABF3) to regulate ABA signal output in *Arabidopsis* as well (Lee et al. 2020; Sirichandra et al. 2010). 14‐3‐3 𝜀 can regulate polar transport of auxin in *Arabidopsis* seedlings (Keicher et al. 2017). Only two literature studies reported that 14‐3‐3 proteins can regulate ethylene biosynthesis through stabilising 1‐aminocyclopropane‐1‐carboxylate synthase (ACS) (Yoon and Kieber 2013; Catalá et al. 2014), but the roles of 14‐3‐3 proteins in ethylene signalling and fruit ripening in tomato remain still unknown. Our results demonstrated that *SlTFT1* can enhance the YFT1 protein accumulation through preventing YFT1 from degradation mediated by SlETP2‐like3 (Figure 3a,g), and thus, SlTFT1 can promote ethylene signal transduction in tomato (Figure 4b,d) and finally accelerates tomato fruit ripening (Figure 6a,b).

However, the phenotypes of 14‐3‐3 protein SlTFT1 in the ethylene signalling pathway and fruit ripening in tomato clearly differ from those of 14‐3‐3 mutants reported in tomato and other species (Figures 4a,b and 6a,b). For example, 14–3‐3 proteins GRF6 and GRF8 positively regulate plant immunity, and 14‐3‐3 λ/14–3‐3 κ promote shade‐induced stem elongation in *Arabidopsis* (Dong et al. 2023; Huang et al. 2018). Multiple tomato 14‐3‐3 proteins have been reported to regulate resistance to *Xanthomonas* bacteria (Giska et al. 2013). Here, we report that the tomato 14‐3‐3 protein SlTFT1 plays a critical role in regulating the ethylene signalling pathway and fruit ripening in a YFT1‐dependent manner. Based on our study results and these results from other plant species, we propose that the conserved 14‐3‐3 family proteins play critical roles in distinct biological processes in a wide range of plant species.

In this study, we functionally characterised an SlTFT1‐YFT1 module in regulating tomato fruit ripening. The 14‐3‐3 protein SlTFT1 can bind YFT1 through multiple binding motifs and prevent YFT1 protein degradation mediated by SlETP2‐like3. We conclude that the SlTFT1‐YFT1 module can positively regulate ethylene signal transduction and facilitate tomato response to ethylene stimuli such as fruit ripening, chromoplast development and carotenoid accumulation as well. This study provides a clue for us to understand the molecular mechanism of tomato fruit ripening regulated by an SlTFT1‐YFT1 module.

## 4 Materials and Methods

### 4.1 Plant Materials and Growth Conditions

The tomato ( *S. lycopersicum* ) seeds of M82, *yellow‐fruited tomato 1* (*yft1*, originally named *n3122*), were kindly provided by Professor Dani Zamir (The Hebrew University of Jerusalem, Israel). The *yft1* mutant, which has an insertion of a 573 bp fragment at the regulatory region in the *YFT1* allele gene promoter, was identified using a mutant population created from crossing *yft1* with LA1585 ( *S. pimpinellifolium* ) (Gao et al. 2016; Zhao et al. 2020). The *SlTFT1*‐knockout (KO) and overexpression (OE) tomato lines were respectively generated from M82 and *yft1* background tomatoes by *Agrobacterium tumefaciens* ‐mediated transformation, and the obtained materials were given names as *sltft1*, *SlTFT1*‐OE^M82^, *yft1 sltft1* and *SlTFT1*‐OE^*yft1*^. All the tomato lines were grown in a standard greenhouse at the School of Agriculture and Biology, Shanghai Jiao Tong University, Shanghai, China (31°03′32″ N and 121°44′51″ E). According to Gao et al. (2016), the 35 days post‐anthesis (dpa), 47 dpa and 54 dpa were respectively referred to as the MG stage (full‐size green fruit at 35 dpa), the Br stage (the fruit colour from green to tannish‐yellow on < 10% surface, 47 dpa) and the RR stage (fully red ripe, 54 dpa).

Tobacco (*Nicotiana benthamiana*) seedlings were sown and grown in a growth chamber (Prandt, Ningbo, China) with 20 000 Lux under a 16 h light/8 h dark cycle at 23°C. The abaxial leaves of 6‐week‐old tobacco were injected using *A. tumefaciens* (GV3101) harbouring the appropriate constructs by a syringe without a needle. The transformed leaf tissues were used to determine protein subcellular localisation, BiFC assay and fluorescence signal detection.

### 4.2 Constructs and Transformation

To knock out (KO) *SlTFT1* gene from M82 and *yft1* genomes, we employed the CRISPR/Cas9 technique as described in Deng et al. (2018). Two 20‐bp segments of the small guide RNA (sgRNAs) corresponding to the first exon of *SlTFT1* were designed using the CRISPR‐P v2.0 (http://cbi.hzau.edu.cn/CRISPR2/), and the sgRNAs were then fused with the NGG proto‐spacer adjacent motif (PAM) to create gRNAs. A couple of forward and reverse primers (Table S1) with the gRNAs were designed and synthesised according to the pTX043 plasmid. A DNA segment with *Bsa* I sites at both ends was amplified from pTX043 plasmid and ligated into the same sites in pTX041 using the Golden‐gate assembly method. The CDS sequence of the *SlTFT1* gene was amplified from a cDNA library derived from the fruit pericarp of M82 at 47 dpa using a couple of gene‐specific primers (Table S1). The CDS segment was ligated into *Bam*H I and *Xba* I sites downstream of the 2 × CaMV 35S promoter of the pHB plasmid (Mao et al. 2005). The obtained constructs (*SlTFT1*‐KO and *SlTFT1*‐OE) were confirmed by DNA sequencing and then introduced into *A. tumefaciens* EHA105 using a freeze–thaw method (Weigel and Glazebrook 2006). These constructs were used stably to transform M82 and/or *yft1* tomato lines to create *sltft1, SlTFT1*‐OE^M82^, *yft1 sltft1* and *SlTFT1*‐OE^*yft1*^ tomato lines.

The seeds of M82 and *yft1* were sterilised in 75% ethanol (v:v) for 1 min and immersed in 30% sodium hypochlorite (v:v) for 5 min, and were then rinsed using sterile water at least five times (each for 5 min). The seeds were sown on solid MS_0_ medium [4.41 g L^−1^ Murashige and Skoog (MS) powder (Sigma, CA, USA), 30 g L^−1^ sucrose, 2.61 g L^−1^ phytagel (Sigma, CA, USA), pH = 5.8] to germinate under 16 h light/8 h dark at 25°C. The cotyledons/hypocotyls of tomato seedlings were cut into ~0.5/0.2 cm pieces using the sterilised sharp scissors when two cotyledons were fully expanded and pre‐cultured on solid MS_1_ medium [MS_0_ with 0.1 g L^−1^ indole‐3‐acetic acid (IAA; Sangon Biotech Co. Ltd., Shanghai, China) and 1.5 g L^−1^ 6‐benzylaminopurine (6‐BA; Sigma, CA, USA)] and then kept under dark conditions at 25°C for 24 h. The agrobacterial cells (EHA105) harbouring recombinant plasmids of *SlTFT1*‐KO or *SlTFT1*‐OE were proliferated in Luria‐Bertani (LB) liquid medium [5 g of yeast extract, 10 g of NaCl, and 10 g of Tryptone (Sangon Biotech Co. Ltd., Shanghai, China) in one litre, pH 7.0] with 50 mg L^−1^ rifampicin, 50 mg L^−1^ streptomycin and 100 mg L^−1^ kanamycin in a shaker (Peiying, THZ‐C, Suzhou, China) at 28°C for 220 rpm till the OD_600_ value reached 0.6. The agrobacterial cells were centrifuged at 4000 *g* for 15 min at room temperature, re‐suspended in an equal volume of 1/2 MS_0_ liquid medium (2.21 g L^−1^ MS powder, 30 g L^−1^ sucrose in one litre, pH 5.8) with 50 mg L^−1^ acetosyringone (Sangon Biotech Co. Ltd., Shanghai, China) and then incubated at 28°C with shaking at 180 rpm for 45 min.

The tomato explants were immersed in the transformed agrobacterial cells in 1/2 MS_0_ liquid medium and were gently shaken for 6 min. The transfected explants were transferred onto MS_1_ solid medium for co‐culturing at 25°C for 36 h in the dark and then transferred onto the MS_2_ solid medium (MS_1_ with 300 mg L^−1^ carbenicillin and 50 mg L^−1^ kanamycin for *SlTFT1*‐KO or 5 mg L^−1^ hygromycin for *SlTFT1*‐OE) for positive selection. The explants were re‐transferred onto fresh MS_2_ medium every 2 weeks until the green shoots reached 3–5 cm. The shoots were excised with a sterile sharp scalpel and were induced to root on MS_3_ medium (MS_0_ with 300 mg L^−1^ carbenicillin and 30 mg L^−1^ kanamycin for *SlTFT1*‐KO or 3 mg L^−1^ hygromycin for *SlTFT1*‐OE). The regenerated seedlings were transferred into pots with moist perlite to domesticate for 1 week and then were transplanted into big pots with moist soil in a greenhouse with a photoperiod of 16 h light/8 h dark at 25°C for 2 weeks. The positive tomato seedlings were confirmed by PCR using specific primers (Table S1).

### 4.3 Real‐Time Quantitative PCR Analysis

Total RNA samples were extracted from the equatorial pericarp of tomatoes with different genotypes (M82, *sltft1*, *SlTFT1*‐OE^M82^, *yft1*, *yft1 sltft1* and *SlTFT1*‐OE^*yft1*^) using the RNAprep pure Plant Kit (Tiangen, Beijing, China) at 35 dpa, 47 dpa and 54 dpa. Total RNA of 0.5 μg was used as the template to synthesise first‐strand cDNA according to the instructions of the PrimeScriptTM RT Master Mix kit (TAKARA, Dalian, China). The constructed cDNA libraries were diluted 4‐fold with RNA‐free water and were used as the template for real‐time quantitative PCR (RT‐qPCR) analyses. RT‐qPCR was performed on a CFX96 (BioRad, Hercules, CA, USA) instrument using SYBR Green Master mix (TAKARA, Dalian, China). An aliquot of 20 μL reaction volume containing 2 μL of the diluted cDNA, 10 μL of Maxima SYBR Green (Takara, Dalian, China), 0.8 μL of each forward and reverse primer (10 μM, Table S1) and 6.4 μL of RNase‐free water was prepared. RT‐qPCR was conducted at 94°C (initial denaturation) for 3 min and then for 40 cycles of 94°C for 20 s, 55°C for 20 s and 72°C for 20 s. The expression of the target genes was calculated using the 2^−ΔCT^ equation described by Li et al. (2024), and the *SlACTIN* gene (*Solyc03g078400*) was used as an internal reference gene for normalisation.

### 4.4 Yeast Two‐Hybrid (Y2H) Screens and Assay

A cDNA library for yeast two‐hybrid was constructed using mRNAs extracted from the pericarp of M82 tomato (47 dpa). The cDNA library was cloned into the prey vector pGADT7. The CDS sequence of YFT1‐C, according to Qiao et al. (2012), was amplified from the tomato cDNA library using specific primers (Table S1) and was ligated into the bait vector of pGBKT7. YFT1‐C was used as a bait to screen the tomato cDNA library using the yeast two‐hybrid (Y2H) technique according to the instructions of the Matchmaker Library Construction and Screening Kits (Cat. PT3955‐1, USA). The Y2H assays were performed according to the manual of Matchmaker Gold Yeast Two‐Hybrid System Kits (Cat. 630489, USA). Briefly, the transformed yeast cells harbouring both the bait plasmid and the cDNA (prey plasmid) were cultured on solid Minimal Synthetic Dropout media lacking tryptophan and leucine (SD‐TL) at 30°C for 3–4 days. The generated yeast clones were picked and incubated in liquid SD‐TL media at 30°C for 18 h, and the yeast cells were collected by centrifugation at 2500 *g* for 10 min at room temperature and then were re‐suspended with deionised water to OD_600_ = 1.5. The yeast cell suspension of 5 μL was dripped on the SD‐Trp‐Leu‐His (SD‐TLH) and SD‐Trp‐Leu‐His‐Ade (SD‐TLHA) for growth. The positive clones were picked and the prey plasmids were applied for DNA sequencing. The protein–protein interactions can be verified by yeast re‐transformation. The prey plasmids were co‐transformed into yeast cells with the bait (encoding sequence of the YFT1‐C) plasmid, and the obtained transformants were sprayed onto solid dropout media SD‐TLH and SD‐TLHA with 5‐mM 3 amino‐1,2,4‐triazole (3‐AT).

### 4.5 Construction of DNA Plasmids

The CDS sequences of *SlTFT1* and *YFT1‐C* were amplified from the cDNA library of pericarp of M82 tomato (47 dpa) using specific primers (Table S1) and then were separately ligated into the *Bam*H I site of pXY106‐nYFP and pXY104‐cYFP (Cao et al. 2021), into the *Bam*H I and *Sal* I sites of pCAMBIA1300‐nLUC and into the *Bam*H I and *Kpn* I sites of pCAMBIA1300‐cLUC, into the *Xho* I site of pGreen‐6HA and into the *Nco* I and *Spe* I sites of pHB‐GFP, to create three couples of plasmids of nYFP‐SlTFT1/YFT1‐C‐cYFP for BiFC, SlTFT1‐nLUC/cLUC‐YFT1‐C for LCI and SlTFT1‐6HA/YFT1‐C‐GFP for Co‐IP assays. Then, *SlTFT1* and *YFT1‐C* were separately ligated into the *Nco* I and *Spe* I sites of pCAMBIA1301‐YFP for protein subcellular localisation analysis. All the constructs were confirmed by DNA sequencing.

A series of *YFT1‐C* derivatives, including the mutated and the truncated versions, were created by an overlap PCR technique using the specific primers (Table S1). The mutated *YFT1‐Cs* include *YFT1‐C* ^*S927A*^, *YFT1‐C* ^*S1118A*^, *YFT1‐C* ^*S927A/S1118A*^, *YFT1‐C* ^*S927D*^, *YFT1‐C* ^*S1118D*^ and *YFT1‐C* ^*S927D/S1118D*^. Here, the superscript letters and numbers indicate the sites of amino acid residues in YFT1. The truncated *YFT1‐Cs* include *YFT1‐C1/2/3*, *YFT1‐C2* ^*S927A*^, *YFT1‐C3* ^*S1118A*^, *YFT1‐C2‐1/2/3*, *YFT1‐C2‐1‐1/2*, *YFT1‐C2‐3‐1/2*. These PCR products were cloned into the *Bam*H I site of pXY104‐cYFP using a ClonExpressII One Step Cloning Kit (Vazyme, Nangjing, China).

The sequences of *SlTFT1‐T2A*, *SlETP2‐like3‐T2A* and *YFT1‐C* were amplified from the cDNA library of pericarp of M82 tomato (47 dpa) using specific primers (Table S1), and a DNA segment of *SlETP2‐like3‐T2A‐SlTFT1‐T2A‐YFT1‐C* was created by overlap PCR using specific primers (Table S1). The PCR product was fused into the *Bam*H I site of pHB‐GFP to create *2 × 35S::SlETP2‐like3‐SlTFT1‐YFT1‐C*. In here, three individual segments of *SlETP2‐like3, SlTFT1* and *YFT1‐C* were constructed as a single open reading frame (ORF), and the two adjacent genes were separated by a sequence encoding the T2A peptide [(GSG)EGRGSLLTCGDVEENPGP]. The co‐expressed products of these three genes were cleaved into individual proteins (Gao et al. 2023). Meanwhile, the DNA segments of *SlETP2‐like3*‐*T2A‐6HA‐T2A‐YFT1‐C* and *6HA‐T2A‐6HA‐T2A*‐*YFT1‐C* were respectively cloned into the *Bam*H I site of pHB‐GFP to create *2 × 35S::SlETP2‐like3‐6HA‐YFT1‐C* and *2 × 35S::6HA‐6HA‐YFT1‐C* and used as controls.

The CDS sequences of *SlTFT1*, *SlEIL1* and *YFT1‐C* amplified from the tomato cDNA library were separately ligated into *Bam*H I and *Eco*R I sites of the pGreenII‐0000 to construct effector plasmids, *35S*::*SlTFT1*/*SlEIL1*/*YFT1‐C‐GFP*. The upstream 2‐kb DNA fragments of the start codon ATG of *RIN*/*NOR*/*FUL1/SlTFT1/YFT1* genes were amplified from genomic DNA of M82 tomato using specific primers (Table S1) and then were separately ligated into *Hin*d III site of pGreenII‐0800‐LUC to create reporter plasmids, *35S*::*REN*‐*pRIN*/*pNOR*/*pFUL1/pSlTFT1/pYFT1*::*LUC*.

### 4.6 Performance of BiFC, LCI, DLR and Co‐IP Assays

The above recombinant plasmids were used to introduce into *A. tumefaciens* GV3101 cells and grown in an LB broth containing 50 mg L^−1^ rifampicin, 50 mg L^−1^ streptomycin and 100 mg L^−1^ spectinomycin and shaken at 28°C, 250 rpm for 18 h till OD_600_ = 0.6–0.8. The *agrobacterial* cells harbouring plasmids were collected by centrifuging at 4000 *g* for 10 min at room temperature. The cell pellets were re‐suspended in MS liquid medium containing 10 mM 2‐morpholinoethanesulfonic acid (MES) and 200 μM acetosyringone (pH 5.8), and the cell suspensions were adjusted to OD_600_ = 0.58–0.61. The re‐suspended agrobacterial cells harbouring appropriate recombinant plasmids were stood at room temperature for 2–4 h before the cells of each combination were well mixed in a ratio of 1:1 for injecting the abaxial leaf of *N. benthamiana* using an injector without needle (KDL, Shanghai, China). The plants were kept at 22°C for 48 h under dark conditions.

The enhanced YFP (eYFP) signals were monitored using a Leica SP5 confocal microscope (Leica TCS SP5 X, excitation 514 nm; emission 522–555 nm). The ImageJ software (http://rsb.info.nih.gov/ij/) was used to quantify the intensities of fluorescence signals and convert into an 8‐bit format, and the fluorescence intensities were integrated from all pixels in the selected areas.

The LCI assay was performed according to Chen et al. (2008). Briefly, the infected tobacco leaves were sprayed with 1 mM luciferin (LUC), and the LUC signal was captured using a CCD camera (Berthold Technologies, Bad Wildbad, Germany) at −20°C with 4 min exposures.

The DLR assay as described in Shen et al. (2023). The constructs of effectors and reporters were separately transformed into *A. tumefaciens* GV3101 cells with plasmid pSoup‐P19. The agrobacterial cells harbouring effector and reporter plasmids were co‐injected into *N. benthamiana* leaves with the volume ratio of 4:1. The empty pGreenII‐0000 of *35S*::*GFP* was used as a negative control. LUC and REN activities were measured with a Dual‐Luciferase reporter kit (Promega, WI, USA) according to the manufacturer's instructions using a GloMax‐96 Microplate Luminometer (Promega, WI, USA). Three biological replicates were used for each effector and reporter combination.

The Co‐IP assay was performed according to Yoon and Kieber (2013) with minor modifications. The transformed tobacco leaves were sampled to extract the total soluble protein (TSP), and the TSPs were incubated with anti‐GFP magnetic beads (Sigma, CA, USA). Subsequently, beads were pelleted and washed using wash buffer (400 mM HEPEs, pH 7.4, 50 mM NaCl, 2 mM EDTA, 10 mM pyrophosphate, 10 mM glycerol phosphate, 0.3% CHAPs, 5 mM Na_3_VO_4_, 5 mM NaF, 1 mM DTT, 200 μM PMSF and 1× protease inhibitor cocktail, Roche, Mannheim, Germany) three times. Then the target proteins were eluted from beads with 2× SDS buffer (100 mM Tris–HCl, pH 6.8, 200 mM DTT, and 4% SDS, 20% glycerol, 0.1% bromophenol blue) in a boiling water bath for 5 min. The protein samples were fractionated and immunoblotted with an anti‐GFP antibody or an anti‐HA antibody (1:5000; Abcam, Cambridge, UK).

### 4.7 Electrophoretic Mobility Shift Assay (EMSA)

The complete *SlTFT1* and *YFT1‐C* coding sequence (CDS) was fused to 6 × His and cloned into the pGADT7 vector for in vitro protein synthesis using the TNT T7/SP6 Wheat Germ Protein Expression System (Promega, WI, USA). EMSA was performed according to Zhang, Liu, et al. 2022. Briefly, the recombinant SlTFT1/YFT1‐C proteins were respectively incubated with fluorescein amidite (FAM)‐labelled probe and competition probe in binding buffer for 30 min at 25°C. Samples were loaded onto a 6% native polyacrylamide gel at 4°C, and the FAM signals were detected by the Cy2 channel of a ChemiDoc MP imaging system (BioRad). All probe sequences are listed in Table S1.

### 4.8 Measurement of Carotenoid Contents

The pericarp of the fruit equatorial region was sampled from different tomato lines, including M82, *sltft1*, *SlTFT1*‐OE^M82^, *yft1*, *yft1 sltft1* and *SlTFT1*‐OE^*yft1*^ at 35 dap, 47 dpa and 54 dpa. The tomato pericarp tissues were ground into fine powder in liquid nitrogen. The carotenoid contents were determined as described in Zhao et al. (2023) with some modifications. The entire process of sample preparation was carried out under dark conditions. Briefly, 1.5 mL of KOH/methyl alcohol (w/v = 6%) solution was added into a 15‐mL conical centrifuge tube containing approximately 500 mg pericarp powder. The mixtures were well homogenised by a vortexer (QL‐901, QiLin, Jiangsu, China) and incubated at 60°C for 30 min with inversion every 10 min. 1.5 mL of Tris–HCl buffer (50 mM Tris–HCl, 1 M NaCl, pH 7.5) was then added when the homogenates turned to room temperature. The samples were well mixed with agitation in an ice bath for 10 min. The homogenates were added 4 mL of chloroform and well mixed in an ice bath for 10 min before the mixtures were centrifuged at 4000 *g* for 10 min at 4°C. The lower organic phase was transferred into a new tube and was further extracted using 4 mL of chloroform for another two times. All the organic phases were collected and combined to dry using an SPD 2010 vacuum concentrator (Thermo Fisher Scientific Inc., MA, USA). The dried samples were re‐dissolved in 50 μL of methyl tertiary butyl ether (MTBE; Thermo Fisher Scientific Inc. MA, USA). 1 μL of the solution was injected into a Waters Acquity Ultra‐Performance Convergence Chromatography (UPC2) system (Waters Corp., Milford, MA, USA) to determine the carotenoid content. Examination of carotenoid contents and the standard curves of lycopene, β‐carotene, α‐carotene and lutein were conducted as described in Zhao et al. (2023). The standards of lycopene, β‐carotene and α‐carotene were purchased from Sigma Chemical Co. (CA, USA), and lutein was obtained from Yuanye Biotechnology Co. Ltd. (Shanghai, China).

### 4.9 Visualisation of Chromoplast Ultrastructure

The pericarp of fruit equatorial region was sampled from M82, *sltft1*, *SlTFT1*‐OE^M82^, *yft1*, *yft1 sltft1* and *SlTFT1*‐OE^*yft1*^ tomatoes at 35 dpa, 47 dpa and 54 dpa, respectively. The samples were processed as described in Schweiggert et al. (2011) with modifications. The pericarp samples were excised into small pieces of ~1 mm^3^ and were immediately immersed in a 2.5% glutaraldehyde [25% glutaraldehyde: 0.1 M phosphate buffer (0.1 M sodium hydrogen phosphate, 0.1 M sodium dihydrogen phosphate): ddH_2_O = v:v:v = 1:5:4, pH 7.3]. The samples were then vacuumised in a vacuum drying cabinet (Yiheng, Shanghai, China) with a vacuum pump (Millipore, MA, USA) for 0.5 h and then were transferred to a refrigerator at 4°C for 24 h. The pericarp samples were rinsed using 0.1 M phosphate buffer (72 mL of 0.2 M sodium hydrogen phosphate and 28 mL of 0.2 M sodium dihydrogen phosphate into 200 mL, pH 7.2) three times (each time for 15 min). The samples were subsequently fixed in 2% osmium tetroxide (w/v in ddH_2_O) for 2 h, washed with 0.1 M phosphate buffer three times (15 min for each time) and then dehydrated in a series of ethanol solutions of 50%, 70%, 90% ethanol, 90% ethanol and 90% acetone (v:v = 1:1) for 15 min each step. Then the samples were dehydrated in pure acetone with shaking at 100 rpm for three times (20 min for each time).

The samples were immersed in a series of solutions of acetone and epoxy resin (Hede Biotechnology Co. Ltd., Beijing, China) in ratios of 1:1 (v:v) for 1 h, 1:2 (v:v) for 1 h and 1:3 (v:v) for overnight, respectively. The samples were then positioned on an embedding plate with 100% epoxy resin for 7 h and kept at 60°C for 48 h to form resin blocks. The sample blocks were cut into 70 nm thick sections using a Leica UC6‐FC6 microtome with a diamond knife (Leica Microsystems Inc., Wetzlar, Germany) and stained with 2% (w:v) lead citrate (Zhongjingkeyi Technology Co. Ltd., Beijing, China). The chromoplast ultra‐structures were observed in a 120 kV transmission electron microscope (FEI, OR, USA).

### 4.10 Measurement of Ethylene Emission

The whole tomato fruits were collected from tomatoes with different genotypes including M82, *sltft1*, *SlTFT1*‐OE^M82^, *yft1*, *yft1 sltft1* and *SlTFT1*‐OE^*yft1*^ at 35 dpa, 47 dpa and 54 dpa (three biological replicates, 3 fruits for each). Those fruits were placed at 25°C for 2 h to remove harvesting stresses, and each fruit was accurately weighed and recorded, and then sealed in a 500 mL airtight bottle (Jiafeng, Shanghai, China) at 25°C for 4 h. An aliquot of 1 mL of headspace gas was taken to inject into a GC‐2010 gas chromatograph (Shimadzu Co. Ltd., Kyoto, Japan). The temperatures of the injector and the flame ionisation detector were, respectively, set at 200°C and 250°C. The initial temperature was set to 40°C for 5 min and increased to 220°C at a rate of 30°C min^−1^. The ethylene content (nL g^−1^ h^−1^) was calculated using an ethylene dose‐dependent curve based on the peak area derived from a series of ethylene concentration gradients containing 0, 0.05, 0.1, 0.3, 0.5 and 0.6 mL of 10 μL L^−1^ ethylene in injection volumes of 1 mL (Weichuang standard gas analytical technology Co. Ltd., Shanghai, China).

### 4.11 Ethylene Triple Response

The tomato seeds of M82, *sltft1*, *SlTFT1*‐OE^M82^, *yft1*, *yft1 sltft1* and *SlTFT1*‐OE^*yft1*^ were germinated on solid medium of 1/2 MS (2.21 g MS powder, 30 g sugar, 2.61 g Phytagel in one litre, pH 5.8) with/without 10 μM ACC (Sigma, CA, USA). The square petri dishes with germinating seeds were placed in a tissue culture chamber at 25°C in the dark. After germination, the root and hypocotyl lengths were measured using a vernier calliper (Mitutoyo, Kawasaki, Japan) at 5 days post‐sowing. At least 15 seedlings were examined for each tomato line.

### 4.12 Protein Extraction and Immunoblotting Analysis

The pericarp was sampled from the tomato equatorial region of M82, *sltft1*, *SlTFT1*‐OE^M82^ tomato lines at 35 dpa, 47 dpa and 54 dpa. The tobacco leaf discs were sampled after transfection for 2 days. The 7‐day‐old tomato seedlings treated with 200 μM cycloheximide (CHX, Acmec Biochemical Co. Ltd., Shanghai, China) or 50 μM MG132 (Sangon Biotech Co. Ltd., Shanghai, China) for 0 h, 1 h and 4 h, and the corresponding tomato seedlings treated with deionised water and DMSO were used for protein extraction.

The tomato pericarps, seedlings and tobacco leaf discs were ground into fine powder in liquid nitrogen. Approximately 1.0 g of powder was mixed with 1.0 mL of protein extraction buffer (1 M Tris–HCl, pH 7.6, 1% Tween, 1 mM EDTA, 150 mM NaCl and 1× protease inhibitor cocktail) to extract the total soluble protein (TSP). The homogenates were placed in an ice bath for 30 min and centrifuged at 13 000 *g* for 10 min at 4°C. The supernatants were transferred to a new precooled Eppendorf tube and were kept in an ice bath for further assay (Zhang, Wu, et al. 2022). The TSP contents in supernatants were determined by the Bicinchoninic acid method (BCA) (Liu et al. 2021).

The immunoblotting analysis was carried out according to Fang et al. (2022)'s method with a minor modification. The TSP contents were adjusted to 1.25 μg/μL using protein extraction buffer. 80 μL of the adjusted TSP was well mixed with 20 μL of 5 × loading buffer (250 mM Tris–HCl, pH 6.8; 0.5 M DTT, 10% SDS, 0.02% bromophenol blue and 30% glycerol) and denatured in a water bath at 95°C for 10 min.

20 μL of supernatant from each sample was loaded into a 12% SDS‐polyacrylamide gel and then subjected to electrophoresis in 1× Tris‐glycine buffer (0.025 M Tris, 0.25 M glycine, 0.1% (w/v) SDS) at 80 V at 4°C for about 2 h. The proteins were transferred to PVDF (polyvinylidene fluoride) (Millipore, Billerica, USA) membranes using a transfer instrument (BioRad, California, USA) in 1× buffer (25 mM Tris‐base, 192 mM glycine, 10% methyl alcohol) at 350 mA for 70 min in an ice bath. The PVDF membranes were blocked using blocking buffer [5% (w:v) non‐fat powdered milk (Sangon Biotech Co. Ltd., Shanghai, China) dissolved in 1× TBST buffer (1 M Tris–HCl, 0.8% NaCl, 0.1% Tween, pH 7.5)] for 1 h at room temperature and were then incubated in 1× TBST buffer with rabbit monoclonal anti‐EIN2/YFT1 antibody (Yougui, Shanghai, China) (v:v = 1:1500) or mouse anti‐ACTIN antibody (Sangon Biotech Co. Ltd., Shanghai, China) (v:v = 1:20 000) for 2 h at room temperature, respectively. The PVDF membrane was washed using 1× TBST buffer three times (10 min for each time) and was then incubated in 1× TBST buffer containing secondary antibody of goat anti‐rabbit IgG (v:v = 1:3000) or rabbit anti‐mouse IgG (v:v = 1:10 000) for 1 h at room temperature. The washed PVDF membrane was sprayed with ECL (enhanced chemiluminescence) (ShareBio, Shanghai, China). The WB signals were detected in ChemiDoc Touch (BioRad, California, USA) and photographed. The signal intensities were calculated using the ImageJ analysis software (Taylor et al. 2013).

## Author Contributions

Lingxia Zhao and Lida Zhang conceived and designed the research and wrote the manuscript; Tengjian Wen performed the main experimental work and wrote the manuscript; Lichun Cao conducted confocal microscopy and EMSA.

## Conflicts of Interest

The authors declare no conflicts of interest.

## Supporting information


**Figure S1.** Interaction of YFT1‐C and five target proteins and their transcriptional expression profile in tomato fruits.
**Figure S2.** Interaction of SlTFT1 with YFT1‐C derivatives.
**Figure S3.** The transcriptional expressions of *SlTFT1* and *YFT1* in different genotype tomatoes.
**Figure S4.** Dynamic changes of the YFT1 protein accumulation in different genotype tomato seedlings treated by deionised water (a) and DMSO (b) as controls.
**Figure S5.** Phylogenetic analysis of SlETPs‐like proteins, and their transcriptional expression images and interaction of these proteins with YFT1‐C.
**Figure S6.** Detection of interaction between *SlTFT1* and *YFT1* by DLR and EMSA.

**Table S1.** Primers used in this study.

## Data Availability

The data that support the findings of this study are available on request from the corresponding author. The data are not publicly available due to privacy or ethical restrictions.
