# Supplementary material for: The 14‐3‐3 Protein SlTFT1 Accelerates Tomato Fruit Ripening by Binding and Stabilising YFT1 in the Ethylene Signalling Pathway
Source: Plant Biotechnol J. 2025 Jul 22;23(11):4872–92. doi: 10.1111/pbi.70274 (PMC12576456; doi:10.1111/pbi.70274)
Supplement: Supplementary file 1 — Figure S1. Interaction of YFT1‐C and five target proteins and their transcriptional expression profile in tomato fruits. Figure S2. Interaction of SlTFT1 with YFT1‐C derivatives. Figure S3. The transcriptional expressions of SlTFT1 and YFT1 in different genotype tomatoes. Figure S4. Dynamic changes of the YFT1 protein accumulation in different genotype tomato seedlings treated by deionised water (a) and DMSO (b) as controls. Figure S5. Phylogenetic analysis of SlETPs‐like proteins, and their transcriptional expression images and interaction of these proteins with YFT1‐C. Figure S6. Detection of interaction between SlTFT1 and YFT1 by DLR and EMSA. [file PBI-23-4872-s002.docx]

**a**


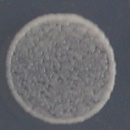

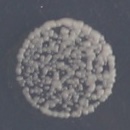


**BD-YFT1-C**


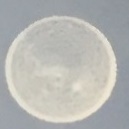

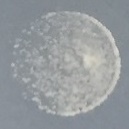

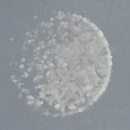

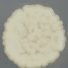

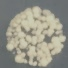

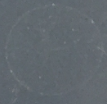

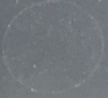

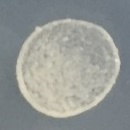

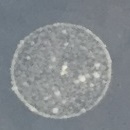

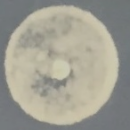

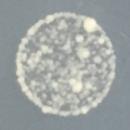


**AD**

**AD-DDTFR5**

**AD-SlMAPKKK67**

**AD-SlTFT1**

**AD-TOMAADEHYD**

**AD-DNA-BP**

**-4SD**


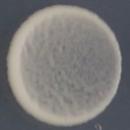

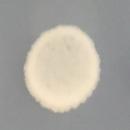

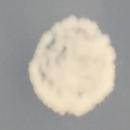

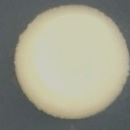

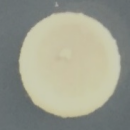


**-3SD**

**-2SD**

**5dpa**

**10dpa**

**20dpa**

**30dpa**

**MG**

**BR**

**PK**

**RR**

***SlTFT1***

**(*Solyc11g010470*)**

***YFT1***

**(*Solyc09g007870*)**

***SlMAPKKK67***

**(*Solyc08g082980*)**

***DDTFR5***

**(*Solyc07g006180*)**

***DNA-BP***

**(*Solyc02g093880*)**

***TOMAADEHYD***

**(*Solyc08g069030*)**

**b**

**RPM**


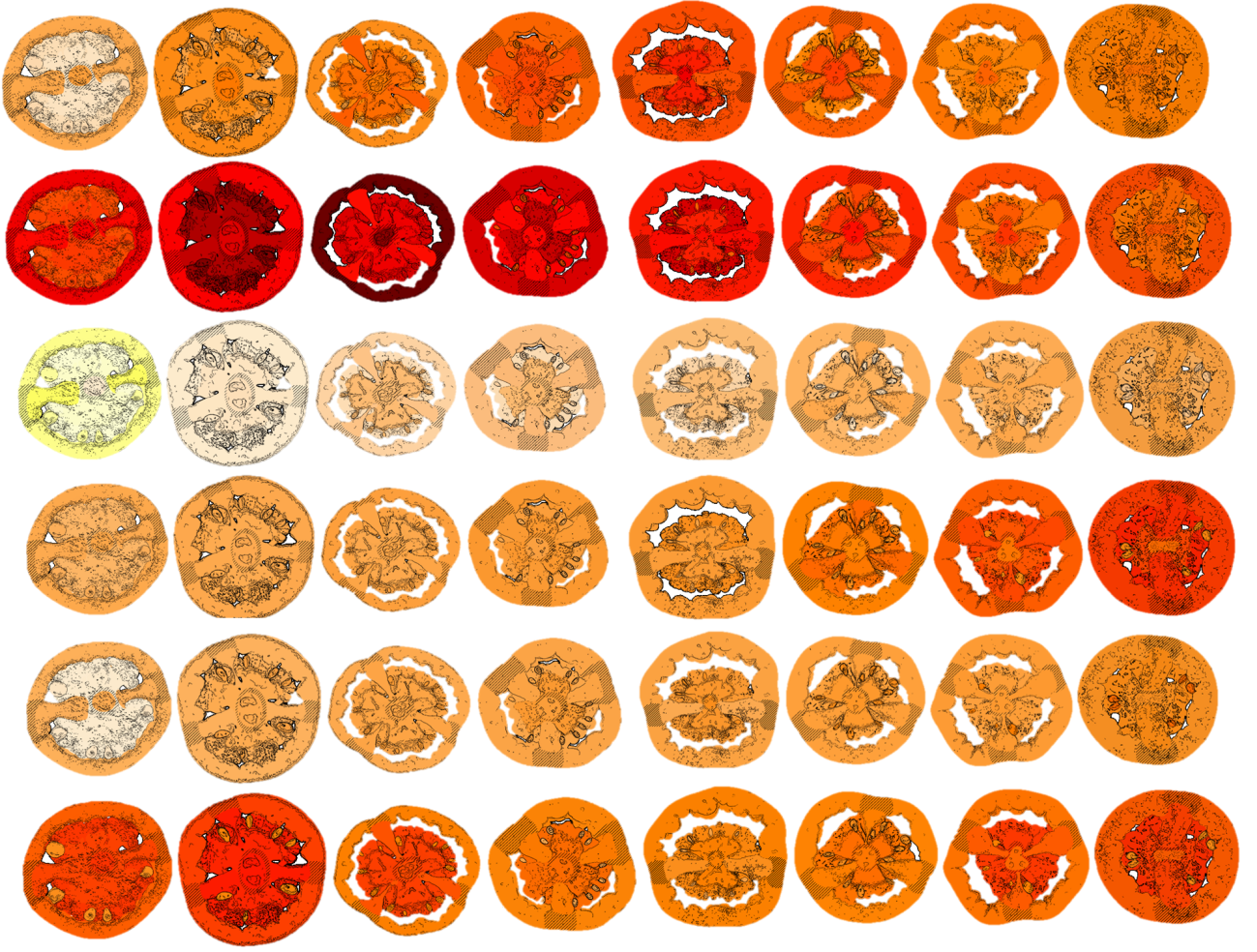

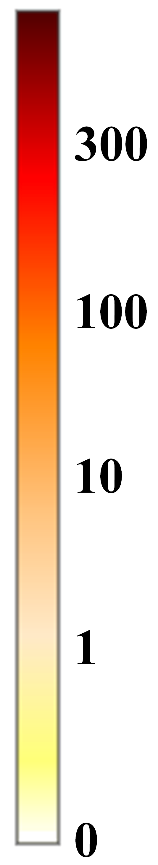


***SlTFT1***

***DDTFR5***

***SlMAPKKK67***

***DNA-BP***

**MG**

***TOMAADEHYD***

**10dpa**

**20dpa**

**30dpa**

**BR**

**RR**

**c**

**5dpa**


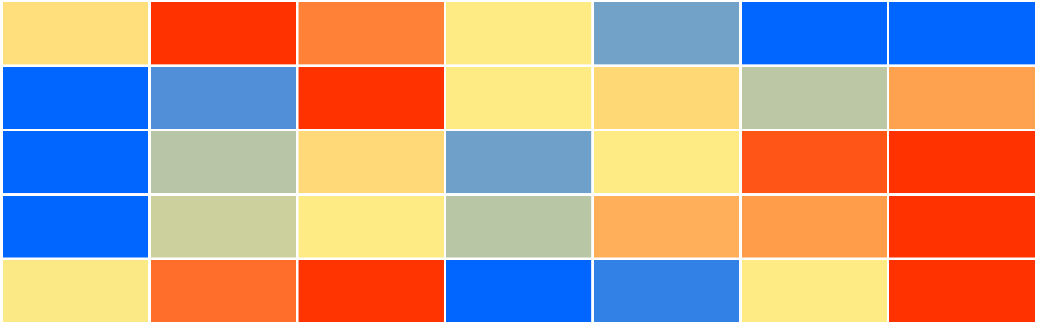

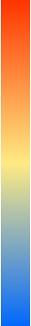


**0**

**440**

**220**

**Supplemental Figure S1** **Interaction of YFT1-C and five target proteins and their transcriptional expression profile in tomato fruits**

**(a)Screened five target proteins from a tomato yeast-expressed library using YFT1-C protein through Y2H assay.**

The plasmids of the pGADT7 harboring CDSs derived from tomato fruit (47 dpa) were extracted, and co-transformed into yeast strain AH109 with pGBKT7-YFT1-C, and obtained five candidate genes**.** *SlTFT1*(*Solyc11g010470*), *SlMAPKKK67* (*Solyc08g082980*), *DDTFR5* (*Solyc07g006180*), *DNA-BP*(*Solyc02g093880*)*,* and *TOMAADEHYD*(*Solyc08g069030*) encode 14-3-3 protein, serine/threonine protein kinase, ripening regulated protein, DNA-binding bromodomain-containing protein, and aminolevulinic acid dehydratase, respectively. To further verify the interaction of those proteins with YFT1-C, the *YFT1-C CDS* sequence was fused to the GAL4 DNA-binding domain (BD) and co-transformed with AD-*SlTFT1*/*SlMAPKKK67*/ *DDTFR5*/*DNA-BP*/*TOMAADEHYD* in yeast strain AH109, respectively. The positive clones were undergone screen on solid media of the -2SD(SD-Trp-Leu)/-3SD(SD-Trp-Leu-His)/-4SD (SD-Trp-Leu- His-Ade). AD, a pGADT7 was used as negative control.

**(b)Tissue-based expression images of *YFT1* and five candidate genes**

The IDs of the *YFT1*, *SlTFT1*, *SlMAPKKK67*, *DDTFR5*, *DNA-BP*, and *TOMAADEHYD* were submitted to the Tomato Expression Atlas platform (<https://tea.solgenomics.net/>) to obtain their expression data. RPM, reads per million mapped reads. Scale bars, 2mm or 5mm for 5 dpa or 10 dpa; 10mm for 20 dpa and 30 dpa; 20mm for mature green (MG), breaker (BR), pink (PK), and red ripe (RR). **dpa**, days post anthesis.

**(c) Expression pattern of five target genes identified through YFT1-C screen**. Data were extracted from the Tomato Expression Atlas platform (<https://tea.solgenomics.net/>), and heatmap was drawn based on the expression levels using conditional formatting function of the Excel.


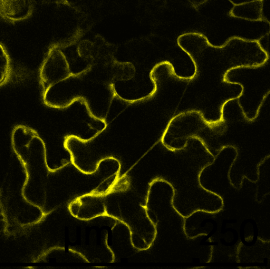

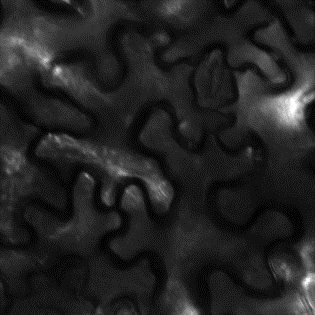

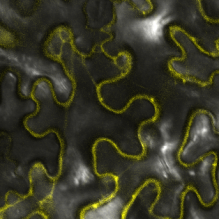

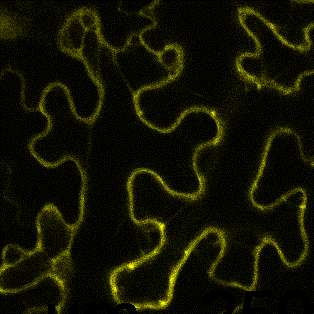

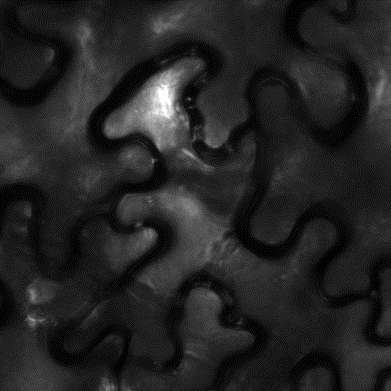

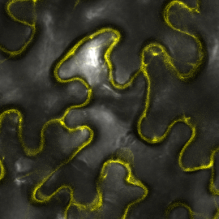

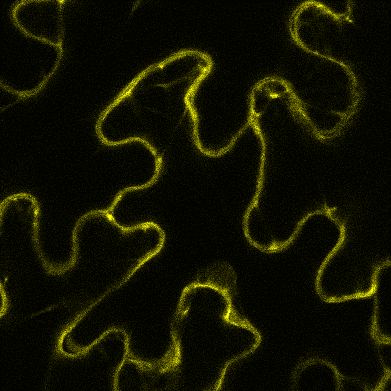

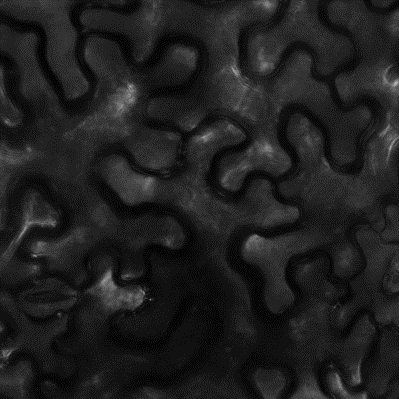

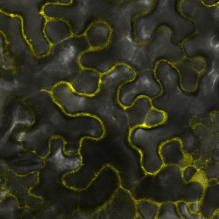

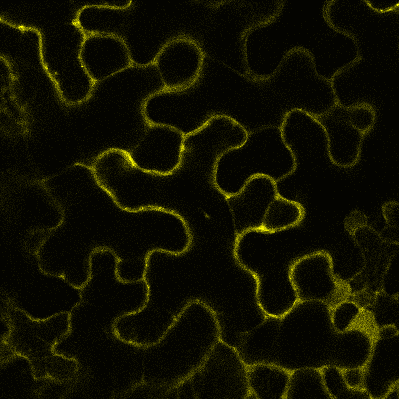

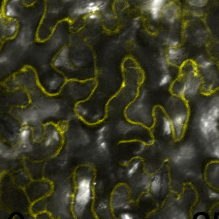

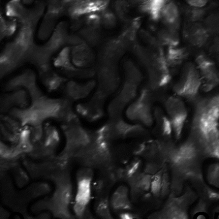

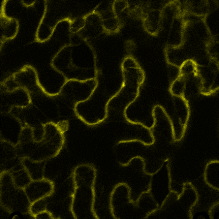

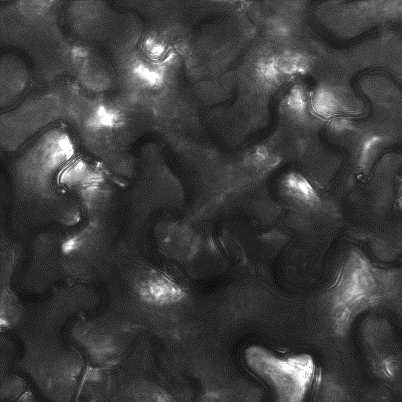

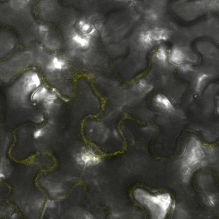

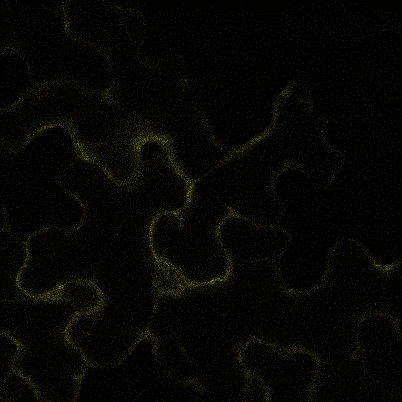

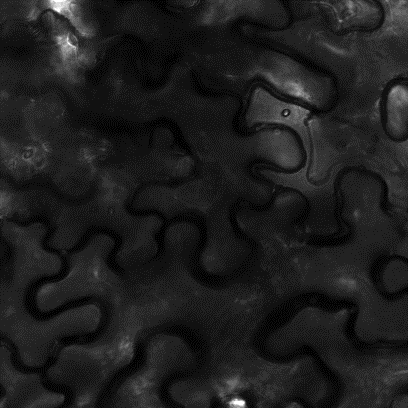

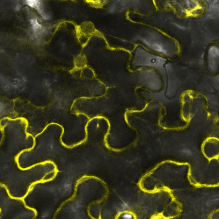

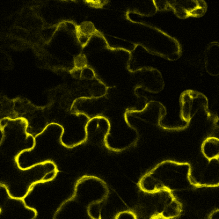


**YFP**

**Bright**

**Merge**

**nYFP-SlTFT1/**

**YFT1-C-cYFP**

**nYFP-SlTFT1/**

**YFT1-C^S927A^ -cYFP**

**nYFP-SlTFT1/**

**YFT1-C ^S1118A^-cYFP**

**nYFP-SlTFT1/**

**YFT1-C ^S927D^-cYFP**

**nYFP-SlTFT1/**

**YFT1-C ^S1118D^-cYFP**

**nYFP-SlTFT1/**

**YFT1-C ^S927A/S1118A^-cYFP**

**nYFP-SlTFT1/**

**YFT1-C ^S927D/S1118D^-cYFP**

**a**


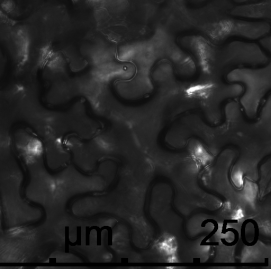

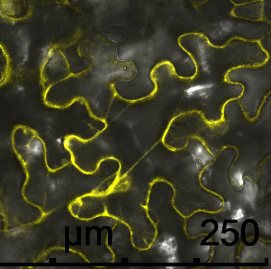


**b**


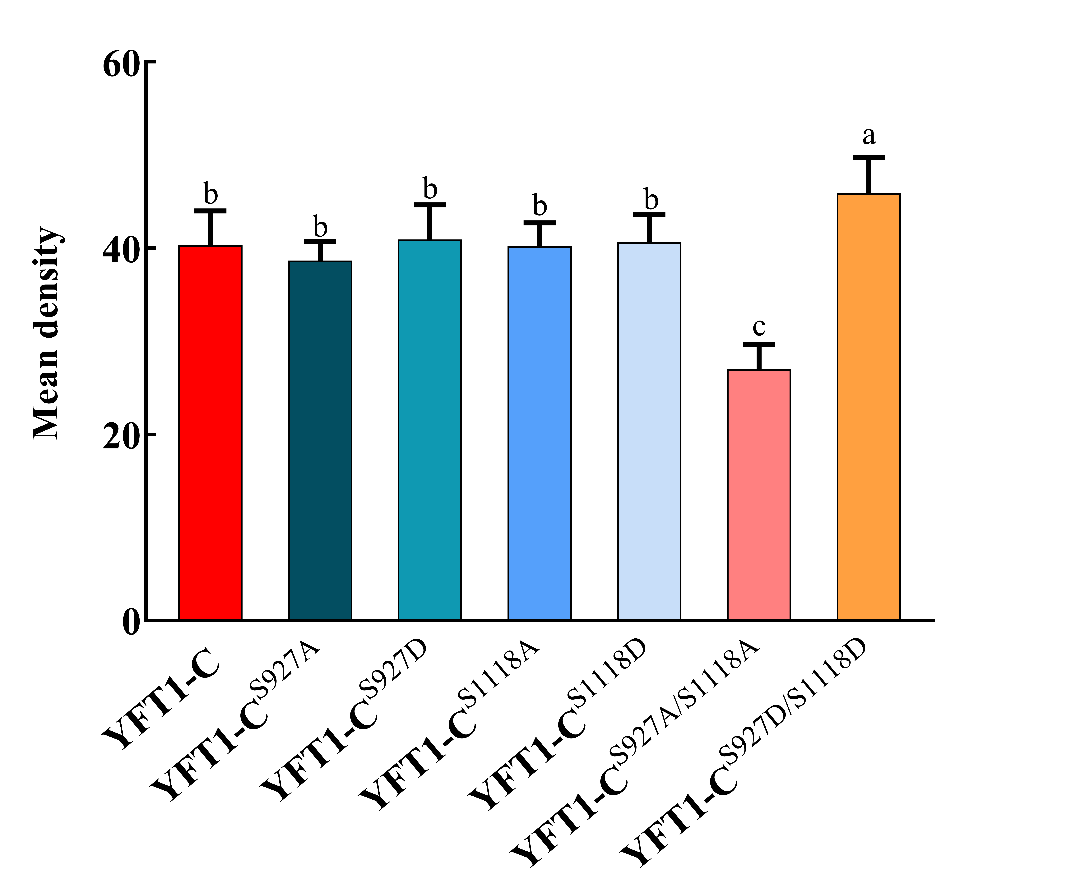


GNTDTVGAAVANEKKYS**S**MPDISGLSMSAR

TETAESVAGSANSKKYY**S**LPDISGRYVPRQ

**:* *. **.*** *:***** : :**

924

907

936

910

927

939

*Arabidopsis thaliana*

*Solanum lycopersicum*

**c**


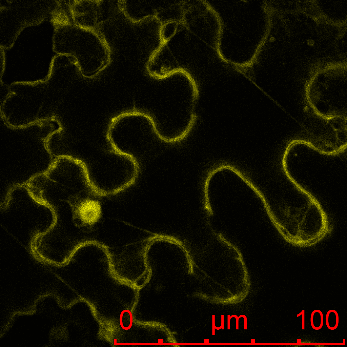

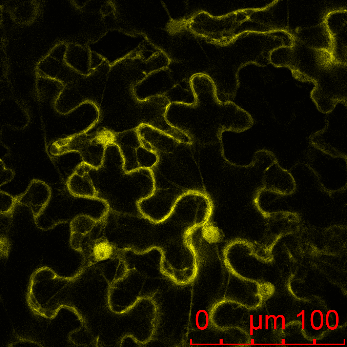

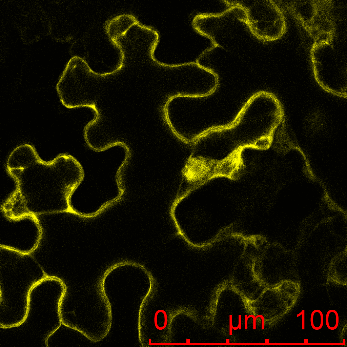

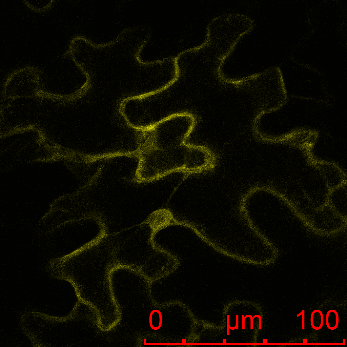


**UL**, nYFP-SlTFT1/YFT1-C2-1-1-cYFP

**UR**, nYFP-SlTFT1/YFT1-C2-1-2-cYFP

**BL**, nYFP-SlTFT1/YFT1-C2-3-1-cYFP

**BR**, nYFP-SlTFT1/YFT1-C2-3-2-cYFP

**d**

**UL**

**UR**

**BL**

**BR**

**Supplemental Figure S2 Interaction of SlTFT1 with YFT1-C derivatives**

**(a) Interaction of SlTFT1 with YFT1-C and its mutated derivatives in transiently transformed *N. benthamiana* leaf.**

The *YFT1-C* and its mutated derivatives(*YFT1-Cmds*) including *YFT1-C^S927A^*，*YFT1-C^S927D^*, *YFT1-C^S1118A^*, *YFT1-C^S1118D^*, *YFT1-C^S927A/S1118A^*，and *YFT1-C^S927D/S1118D^* were created and fused with the encoding sequence of the cYFP to construct a series of binary expression vectors of *35S::YFT1-C-cYFP* and *35S::YFT1-Cmds-cYFP* in plasmid pXY104 skeleton, and then co-transformed into tobacco leaves with *35S::nYFP-SlTFT1* based on the plasmid pXY106. In here, S927A/D and S1118A/D indicate the S at 927 or 1118 site in YFT1-C were substituted by A or D, respectively. The YFP fluorescence signals were detected using a confocal laser scanning microscope. Scale bars, 100 μm.

**(b) Fluorescence density of interaction of SlTFT1 with YFT1-C or YFT1-C mds.** The fluorescence signal density was quantified using Image J software**.** Error bars indicate sd (n=10). Lowercase letter indicate statistical significance at P<0.05 level determined by Duncan test(n=10).

**(c) The alignment of the YFT1-C protein containing canonical motif of 14-3-3 binding to client proteins between *Arabidopsis* and *Solanum lycopersicum****.*

The yellow highlight regions indicate canonical motif, of which, the Ser(S) residue is critical position.

**(d) Interaction of SlTFT1 with YFT1-C2-1-1/2 and YFT1-C2-3-1/2 by BiFC**

UL (upper left), nYFP-SlTFT1/YFT1-C2-1-1-cYFP; UR (upper right), nYFP-SlTFT1/YFT1-C2-1-2-cYFP; BL (bottom left), nYFP-SlTFT1/YFT1-C2-3-1-cYFP; BR (bottom right), nYFP-SlTFT1/YFT1-C2-3-2-cYFP. Scale bars,100 μm.


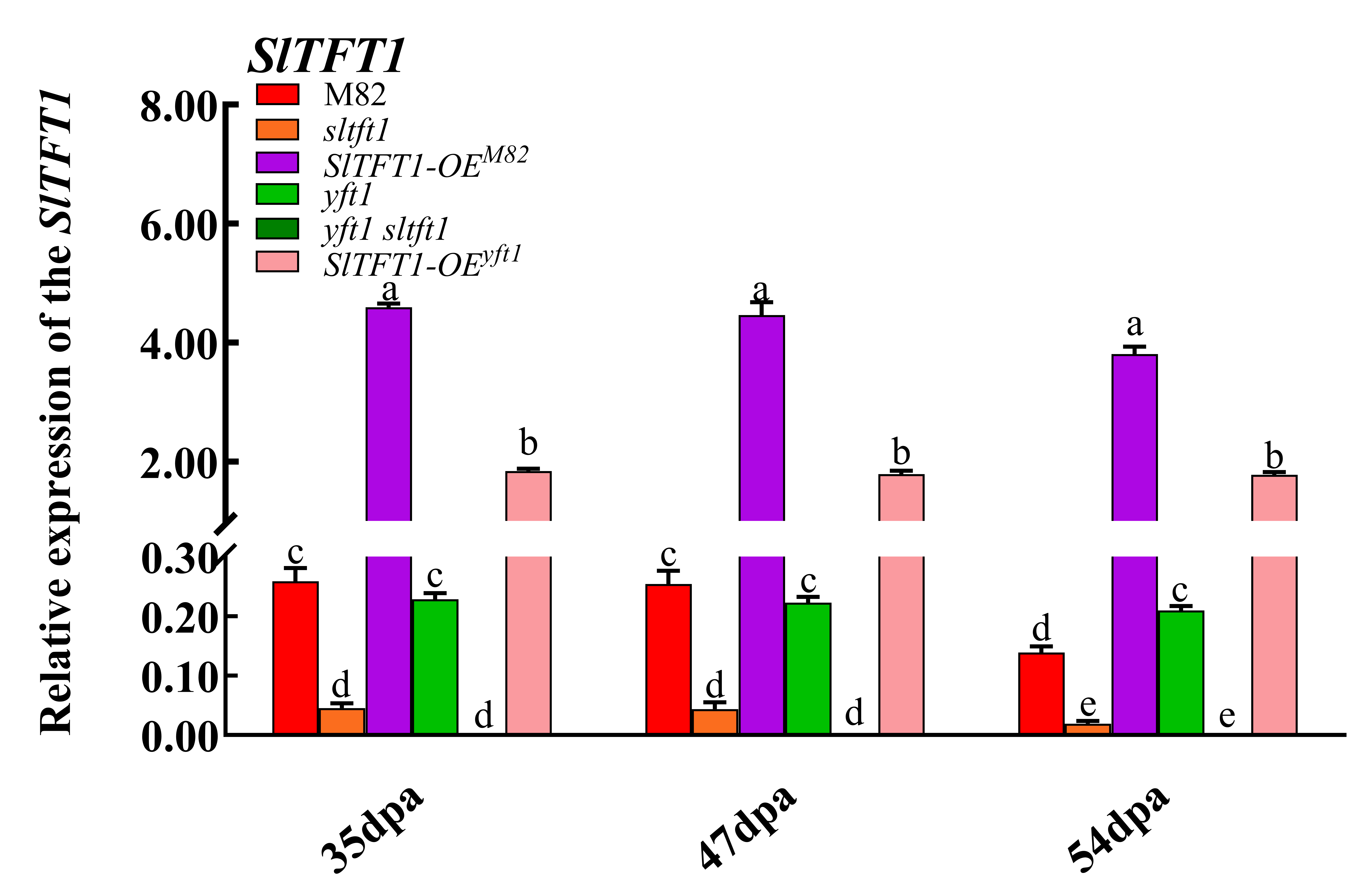

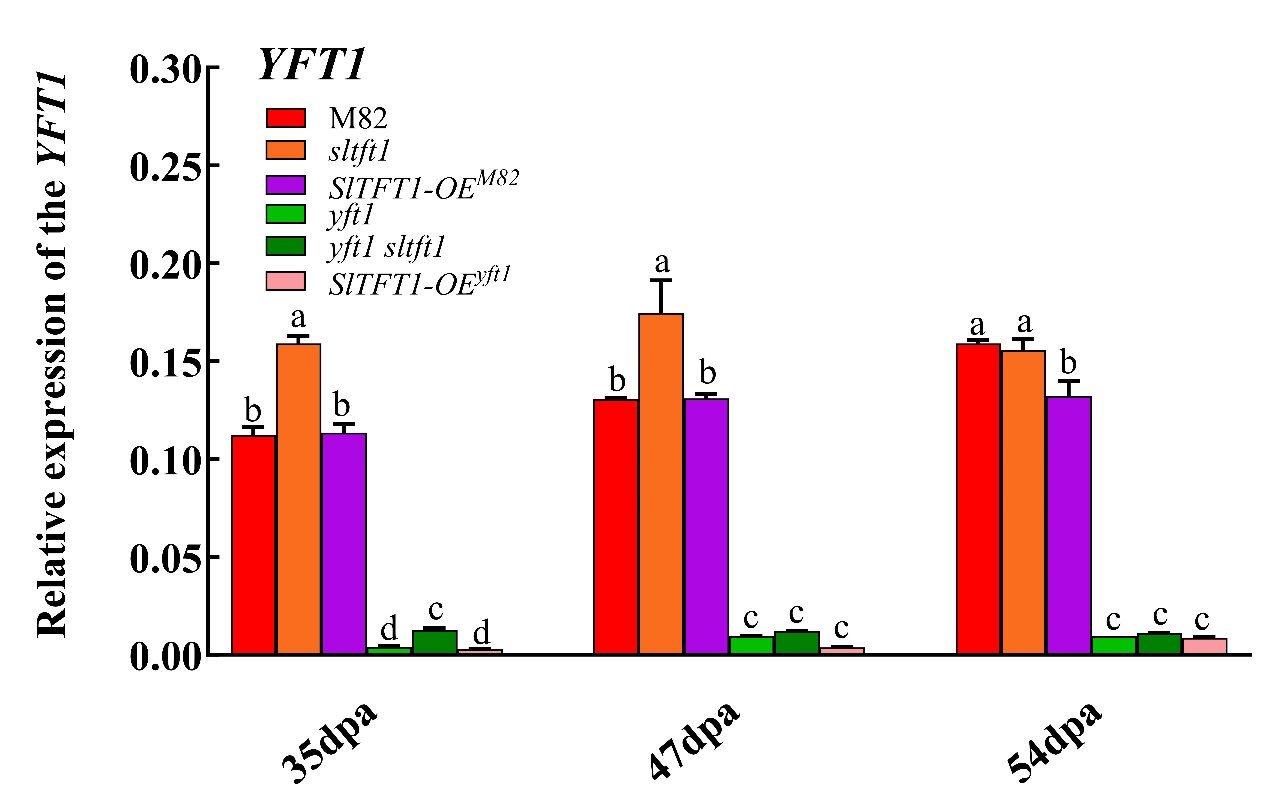


**Supplemental Figure S3 The transcriptional expressions of *SlTFT1* and *YFT1* in different genotype tomatoes.**

M82, a cultivated tomato variety, which was used as wild type in present study; *yft1*, a yellow-fruited tomato mutant was created from M82 by fast-neutron irradiation M82 (original accession *n3122*). *sltft1* and *yft1 sltft1*, indicate that *SlTFT1* knock-out tomato line created from M82 and *yft1* backgrounds using CRISPR-cas9, respectively; *SlTFT1*-OE^M82^ and *SlTFT1*-OE*^yft1^,* indicate the overexpressed *SlTFT1* in M82 and *yft1* backgrounds through *A.tumefaciens* mediated transgene using 2×35S::*SlTFT1-CDS*. Error bars, standard deviation (sd, n=3). Lowercase letters indicate statistical significance at P<0.05 level determined by Duncan test.


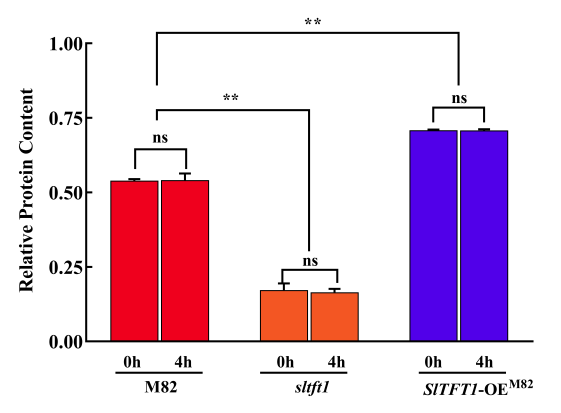

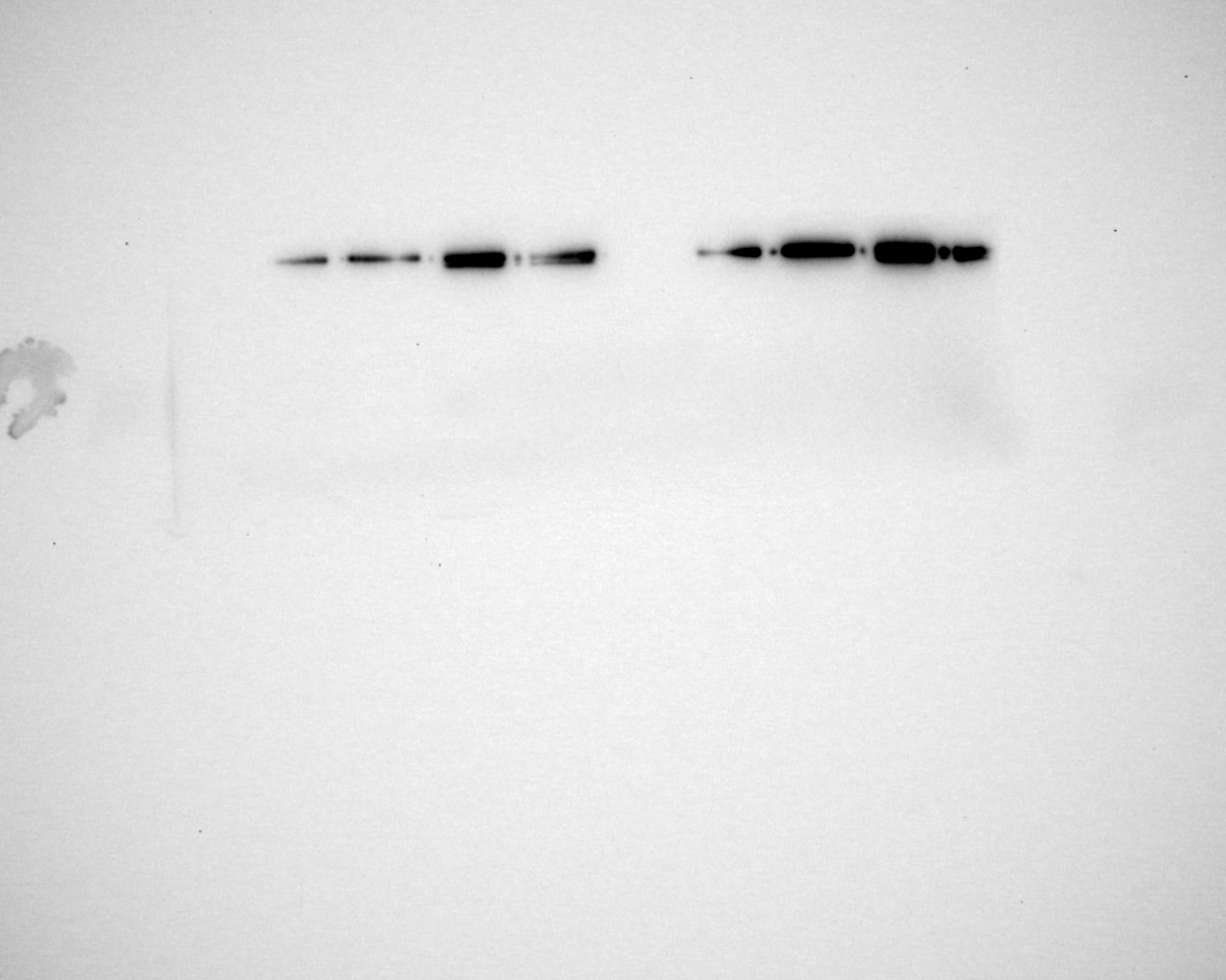

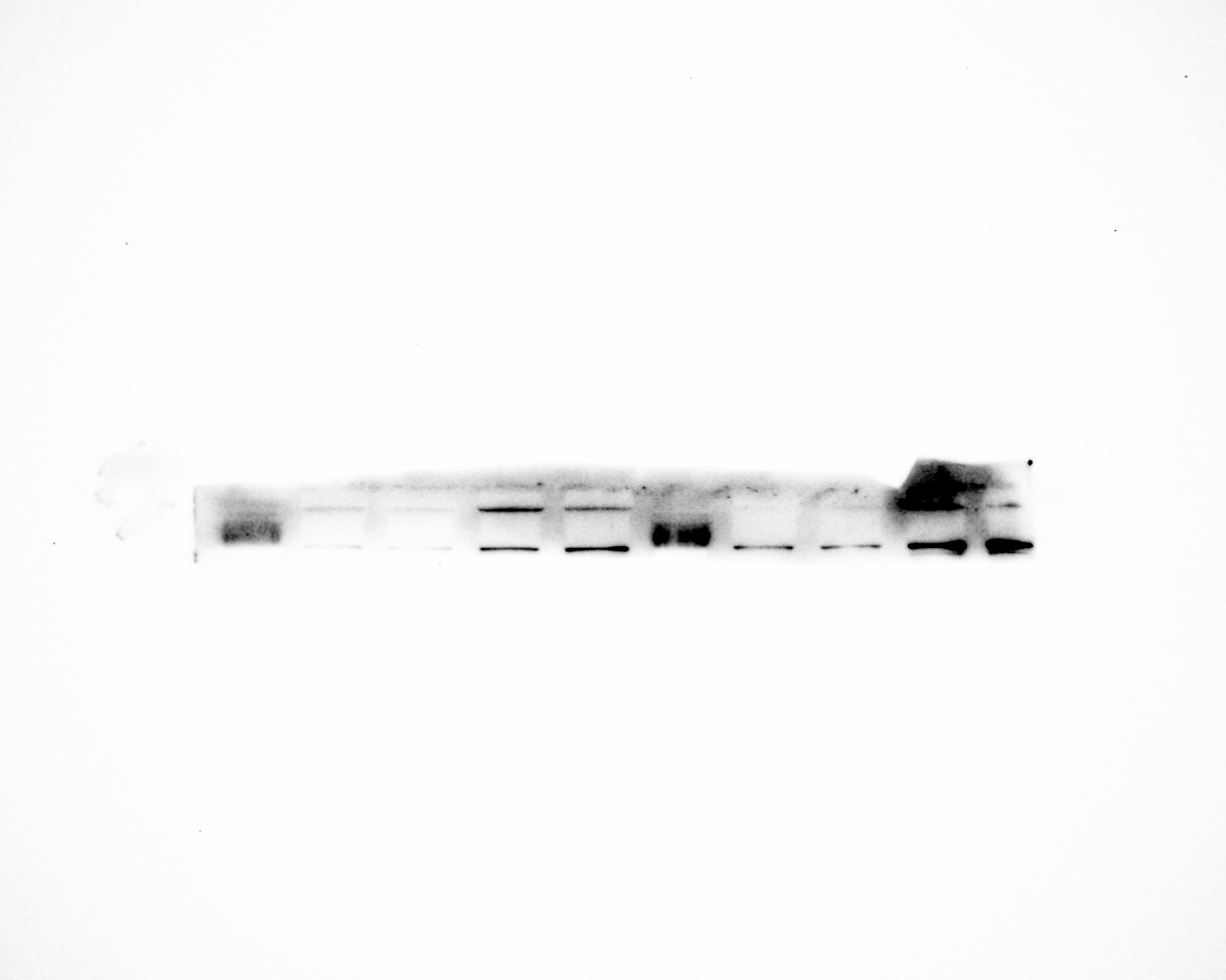

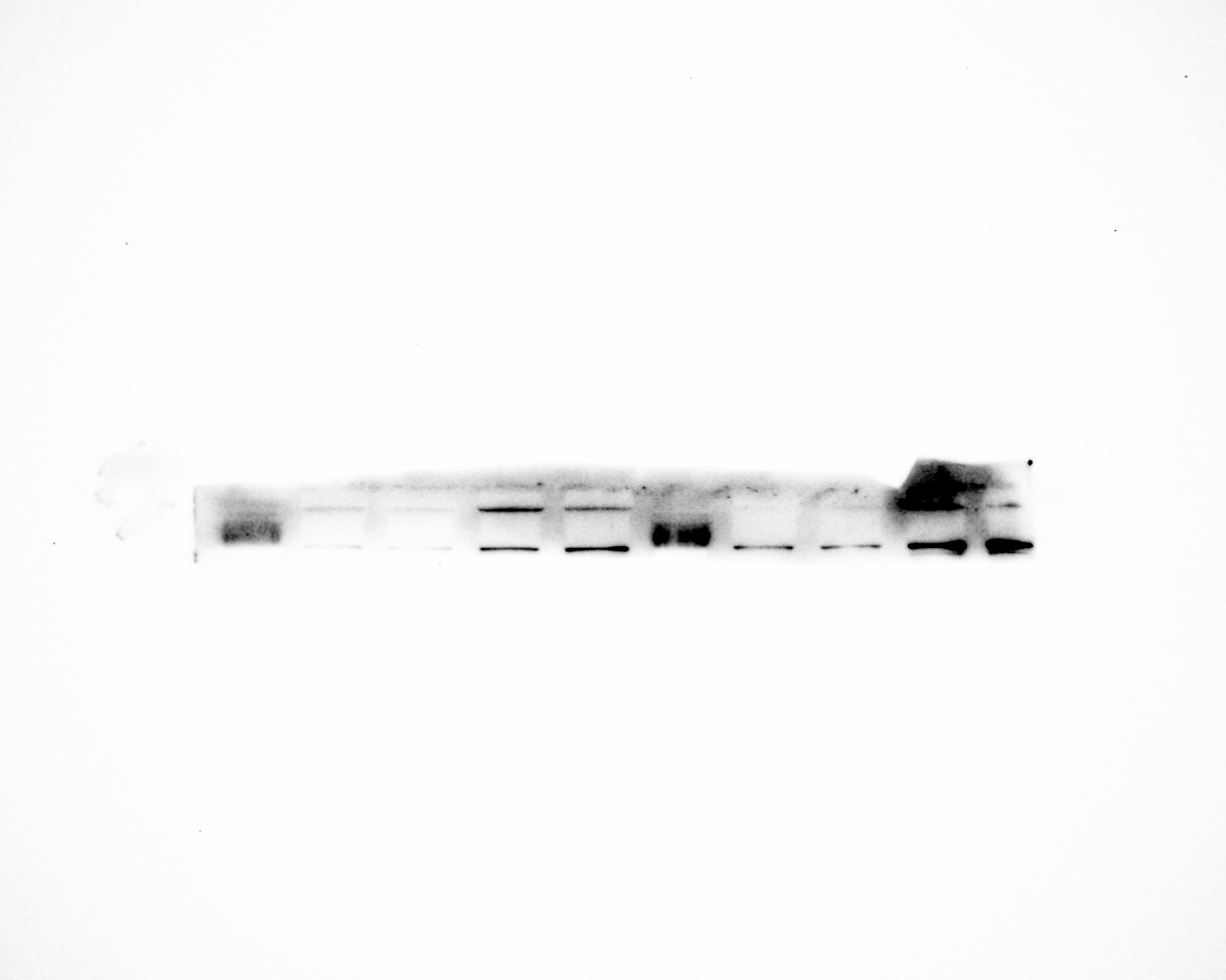

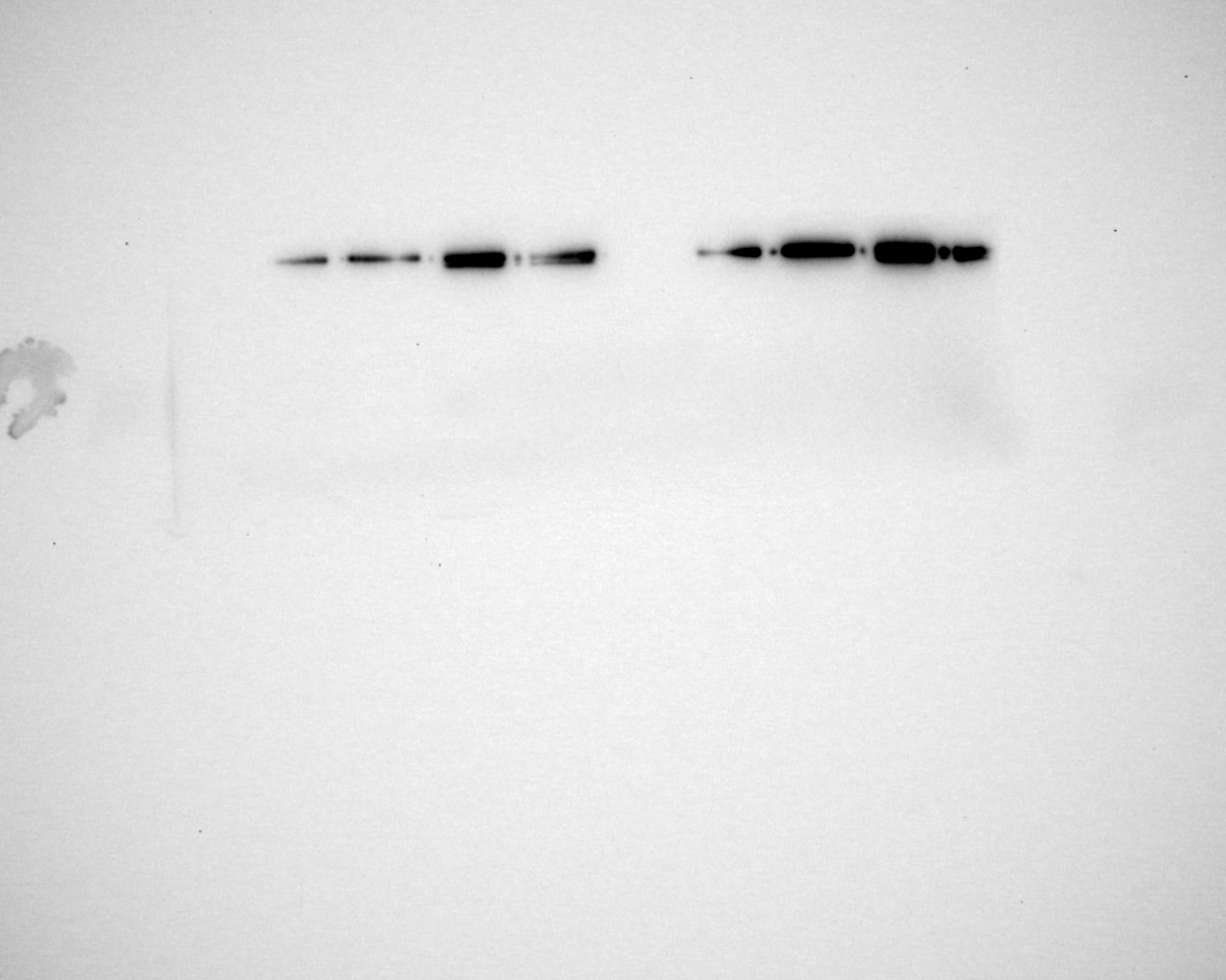

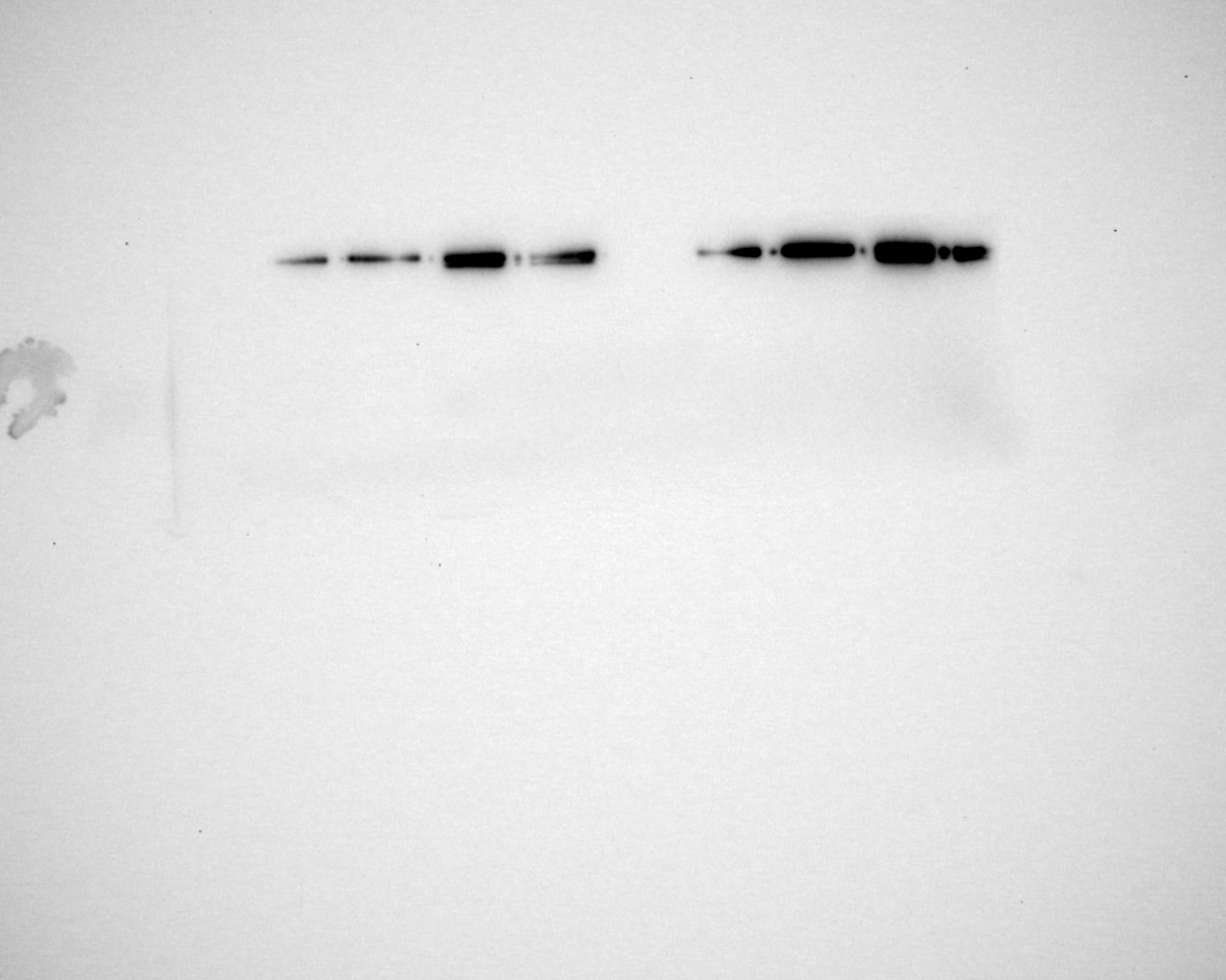

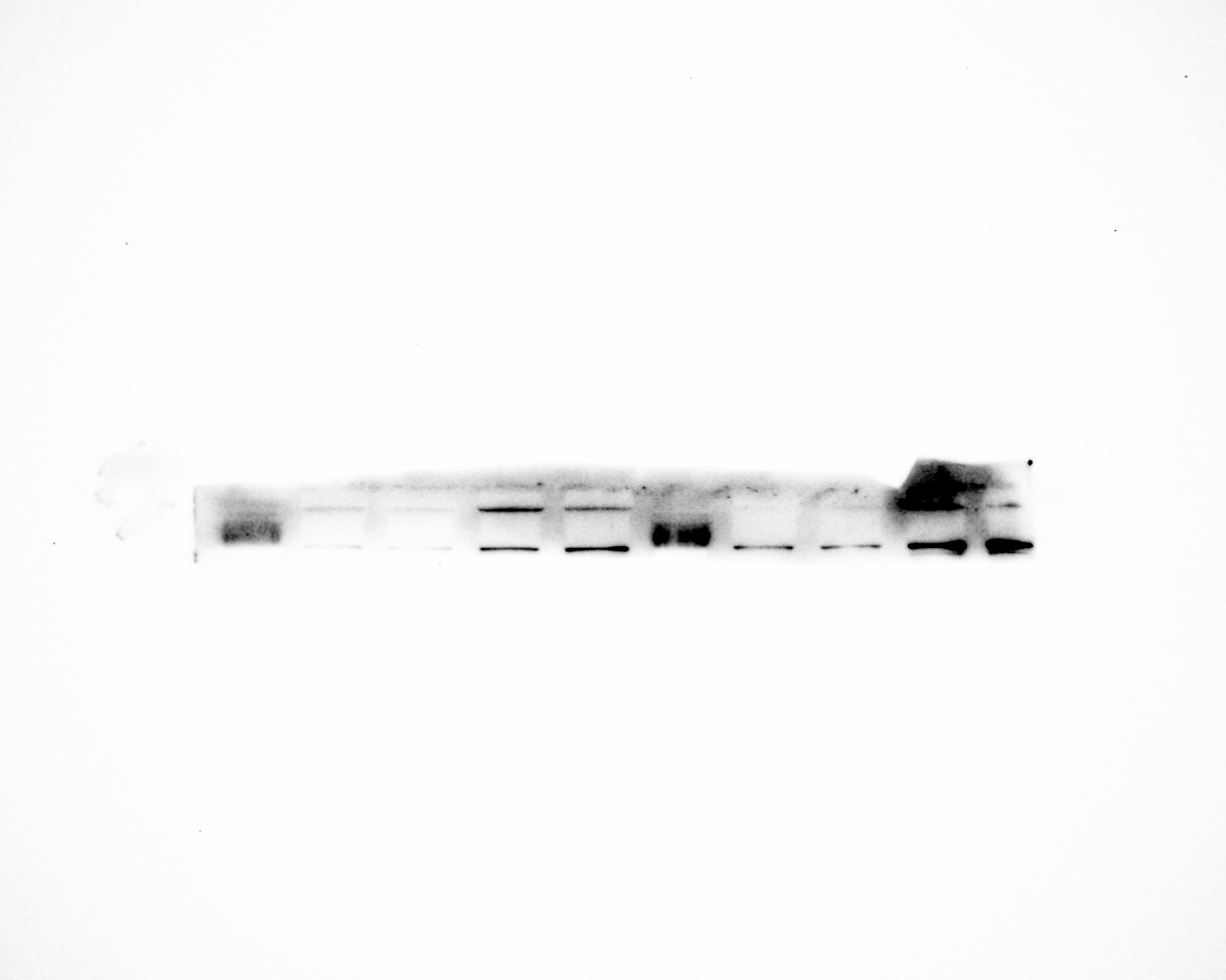


**0h**

**4h**

**0h**

**4h**

**0h**

**4h**

**α-EIN2**

**α-Actin**

**H_2_O**

**a**

**140**

**45**

**0.54**

**0.54**

**0.17**

**0.16**

**0.71**

**0.71**

**M82**

***sltft1***

***SlTFT1-OE^M82^***

**kDa**


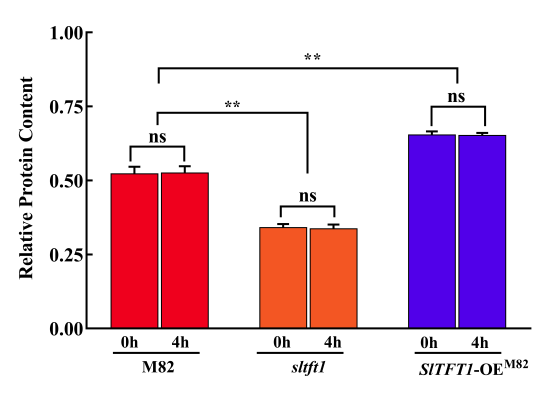

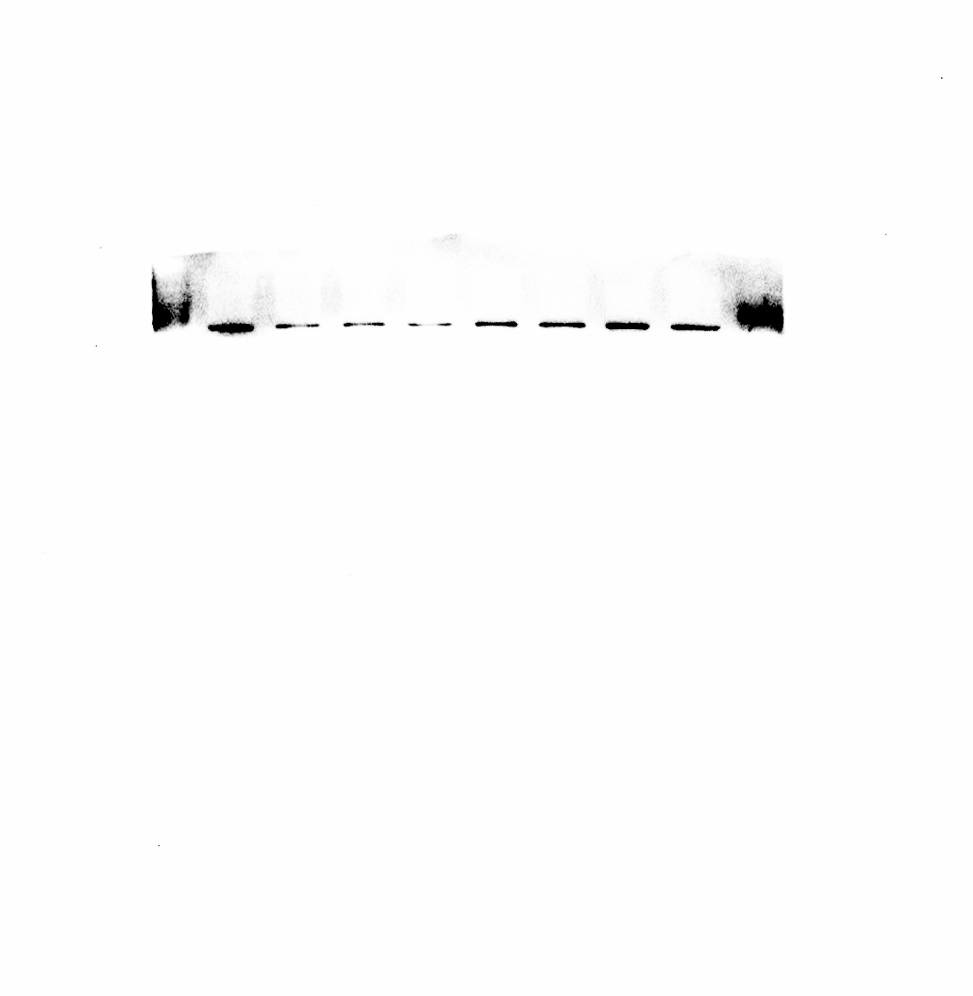

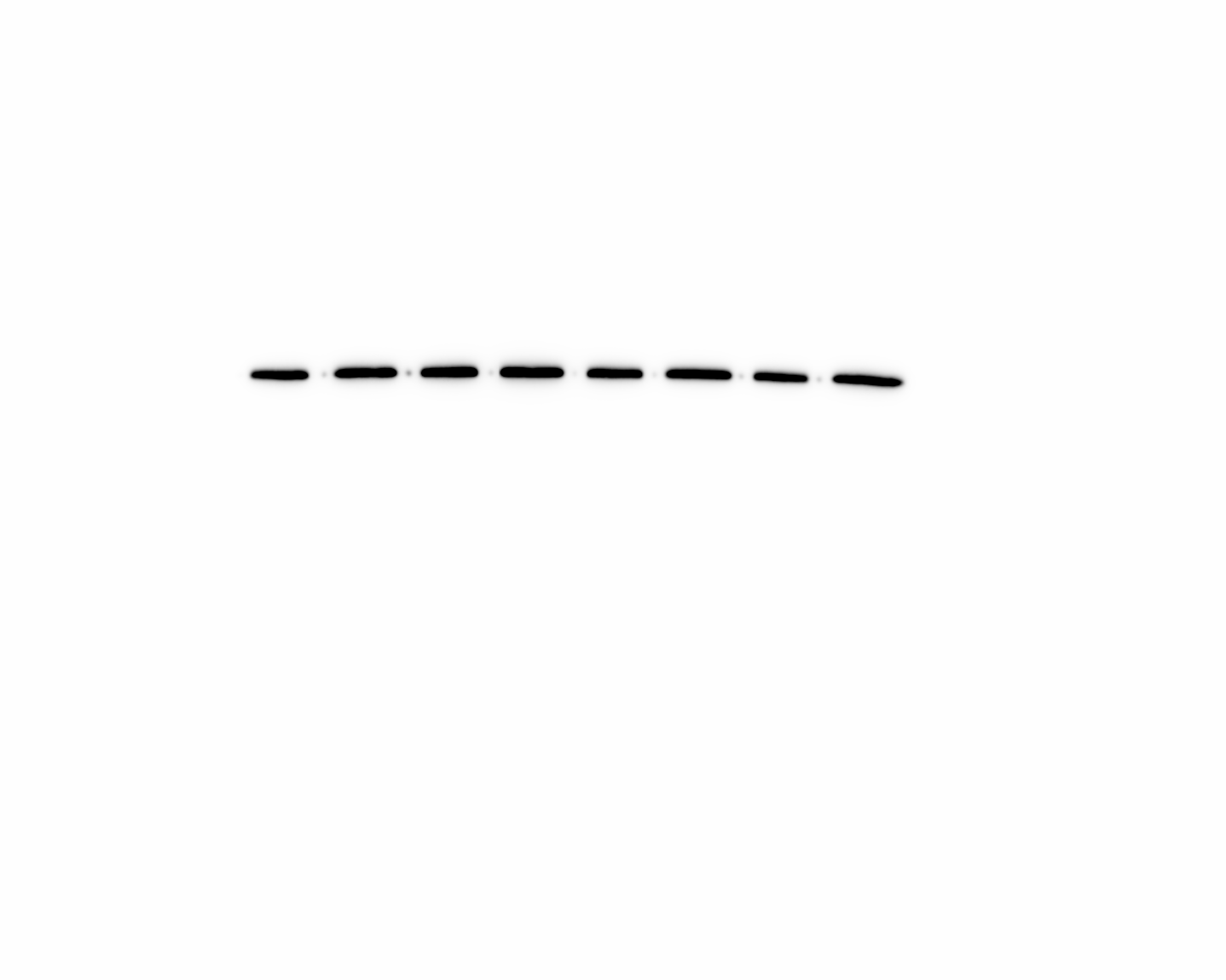

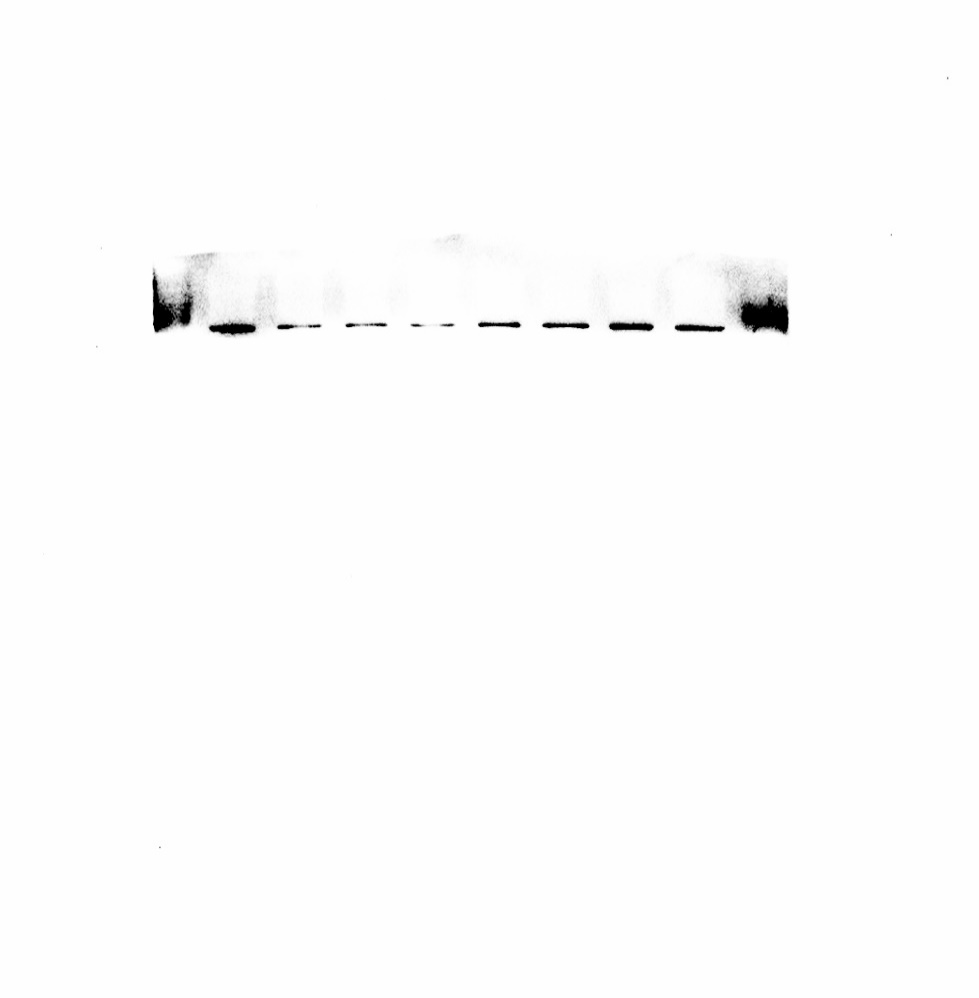

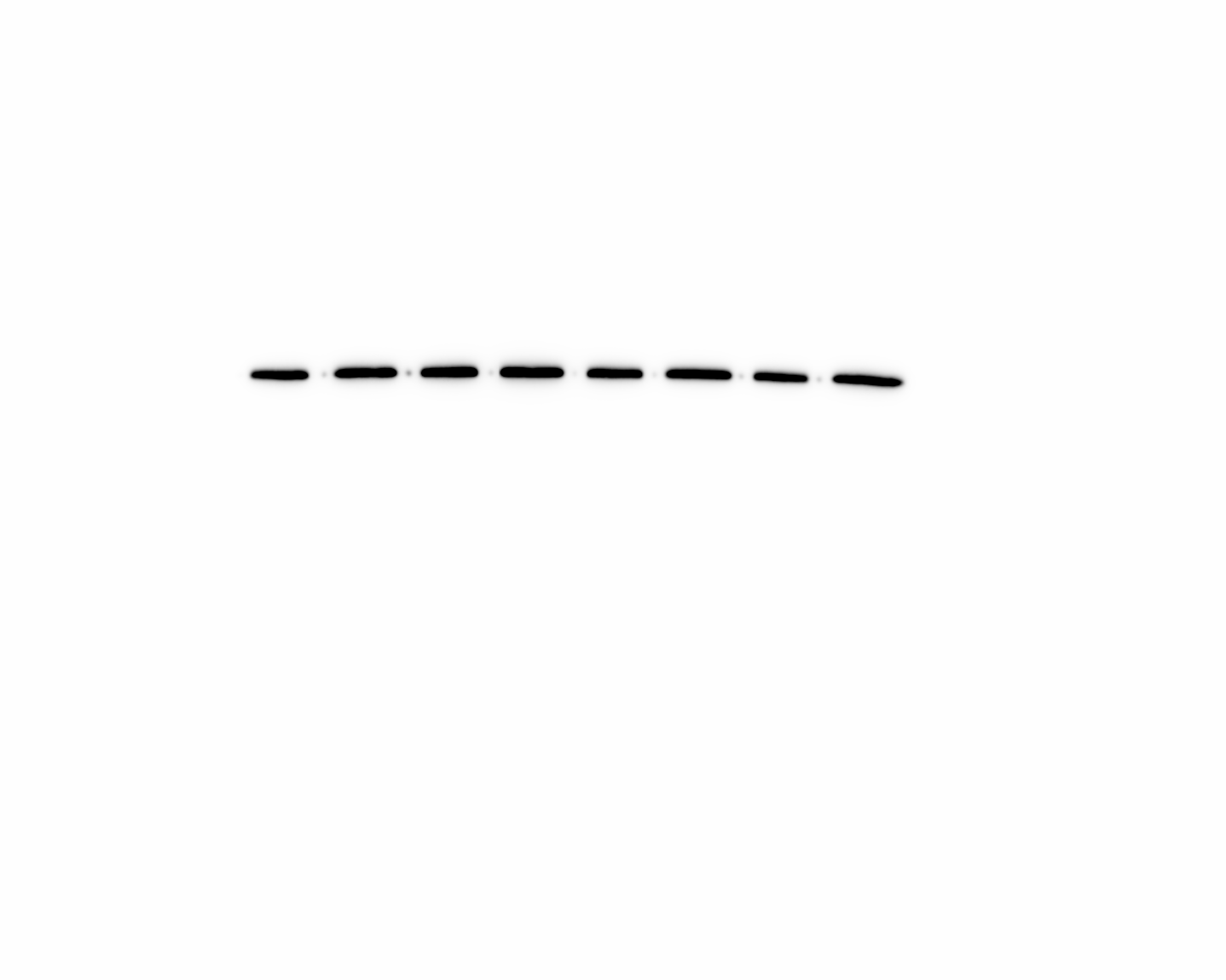

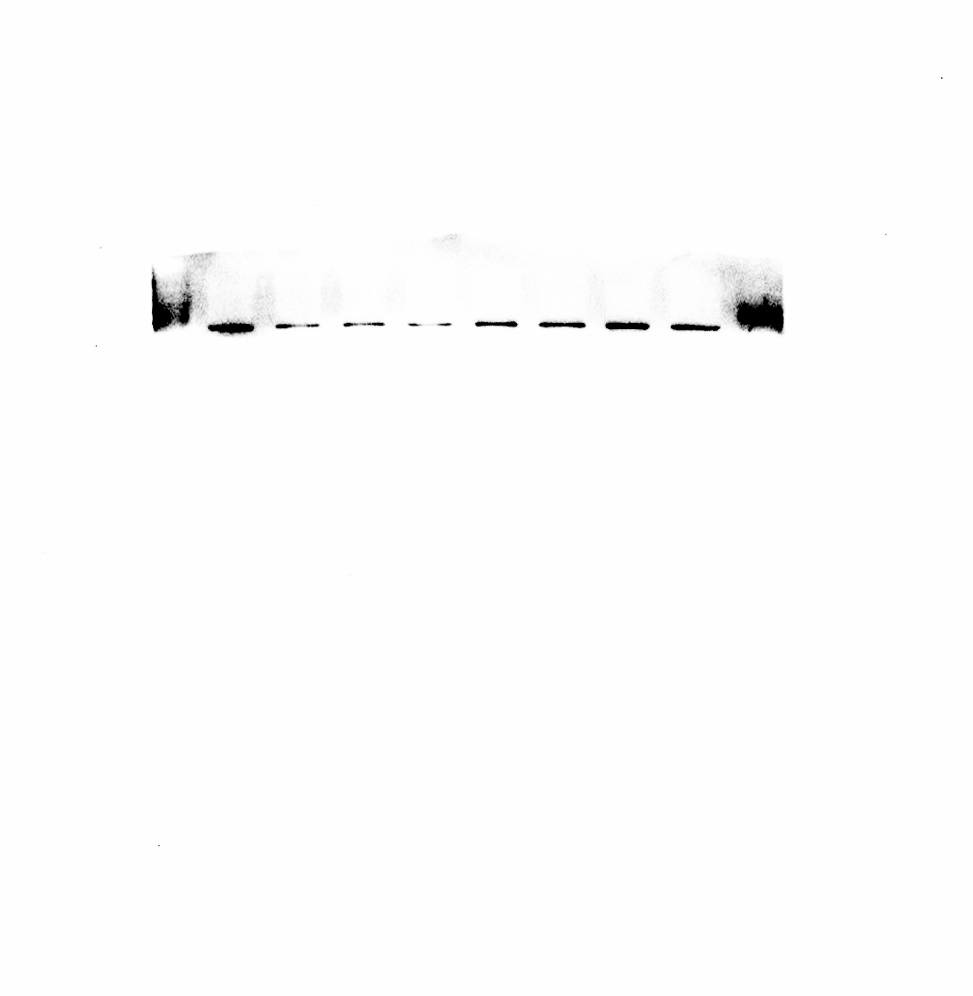

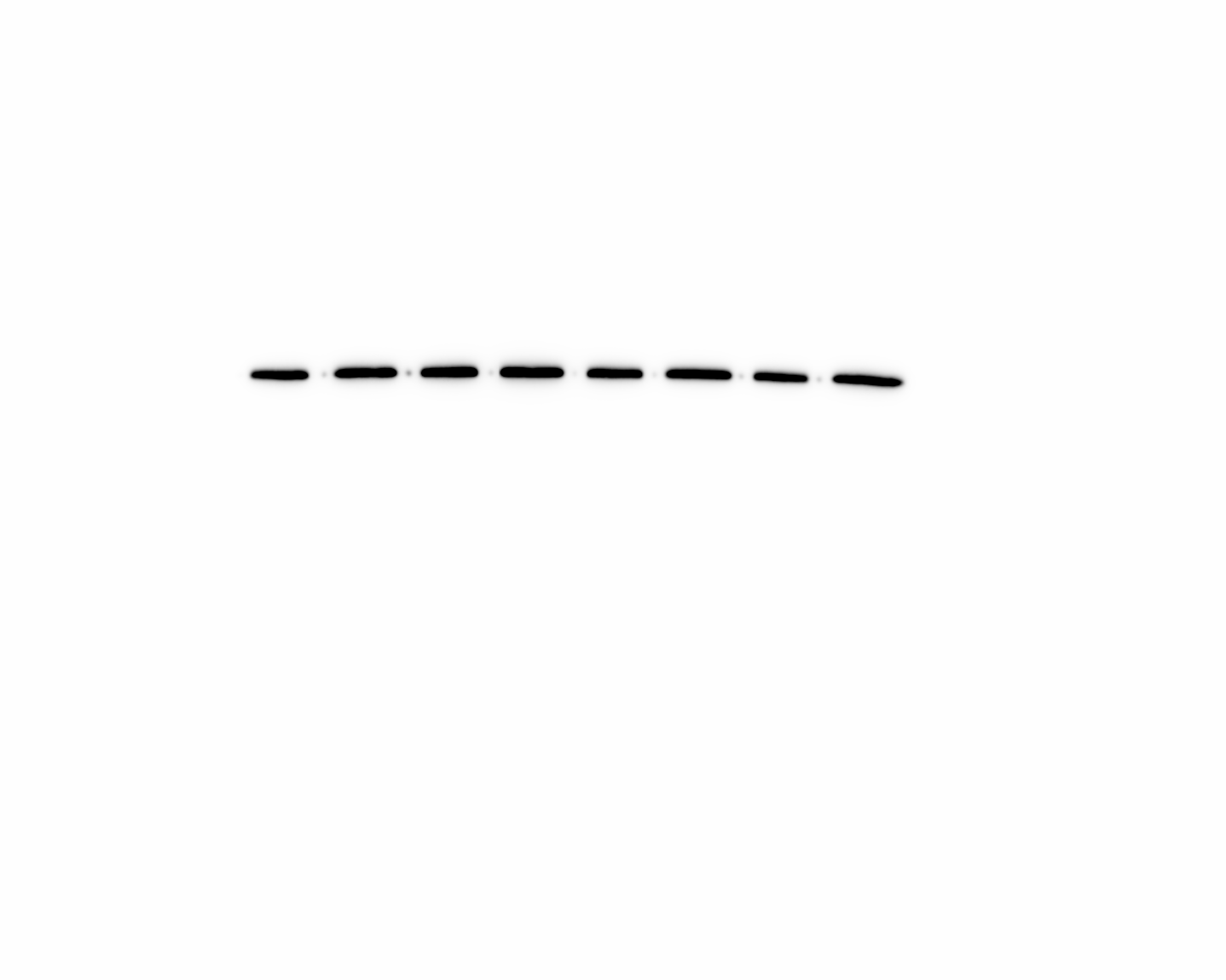


**DMSO**

**b**

**0h**

**4h**

**0h**

**4h**

**0h**

**4h**

**α-EIN2**

**α-Actin**

**140**

**45**

**0.53**

**0.53**

**0.34**

**0.34**

**0.66**

**0.65**

**M82**

***sltft1***

***SlTFT1-OE^M82^***

**kDa**

**Supplemental Figure S4 Dynamic changes of the YFT1 protein accumulation in different genotype tomato seedlings treated by deionized water (a) and DMSO (b) as controls**.

The blots were probed with anti-EIN2(α-YFT1) and anti-Actin(α-Actin) antibodies. Relative intensity of each protein was normalized to the loading control (Actin). α-YFT1, a rabbit anti-EIN2/YFT1 polyclonal antibody; α-Actin, a mouse anti-β-actin (plant) monoclonal antibody.

**a**


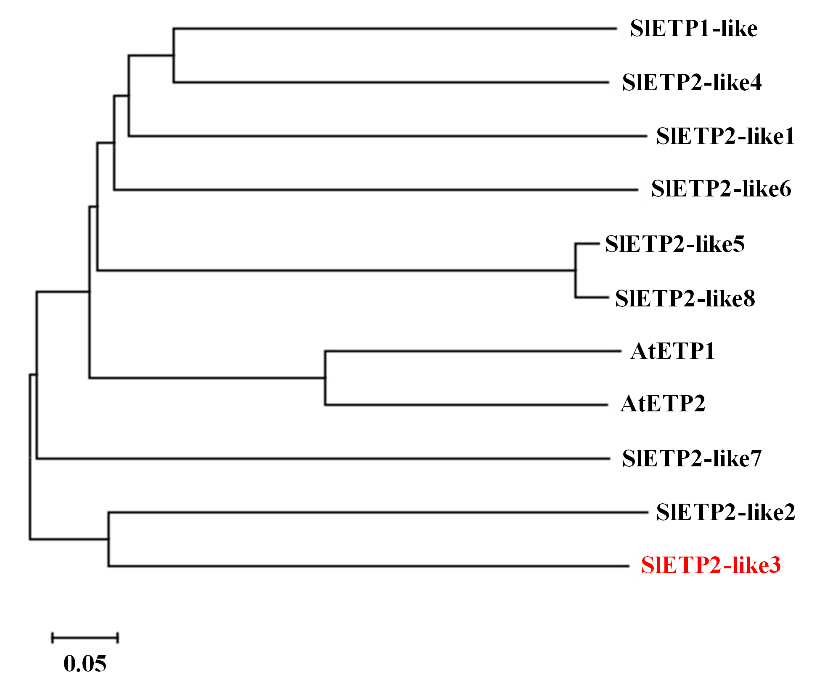

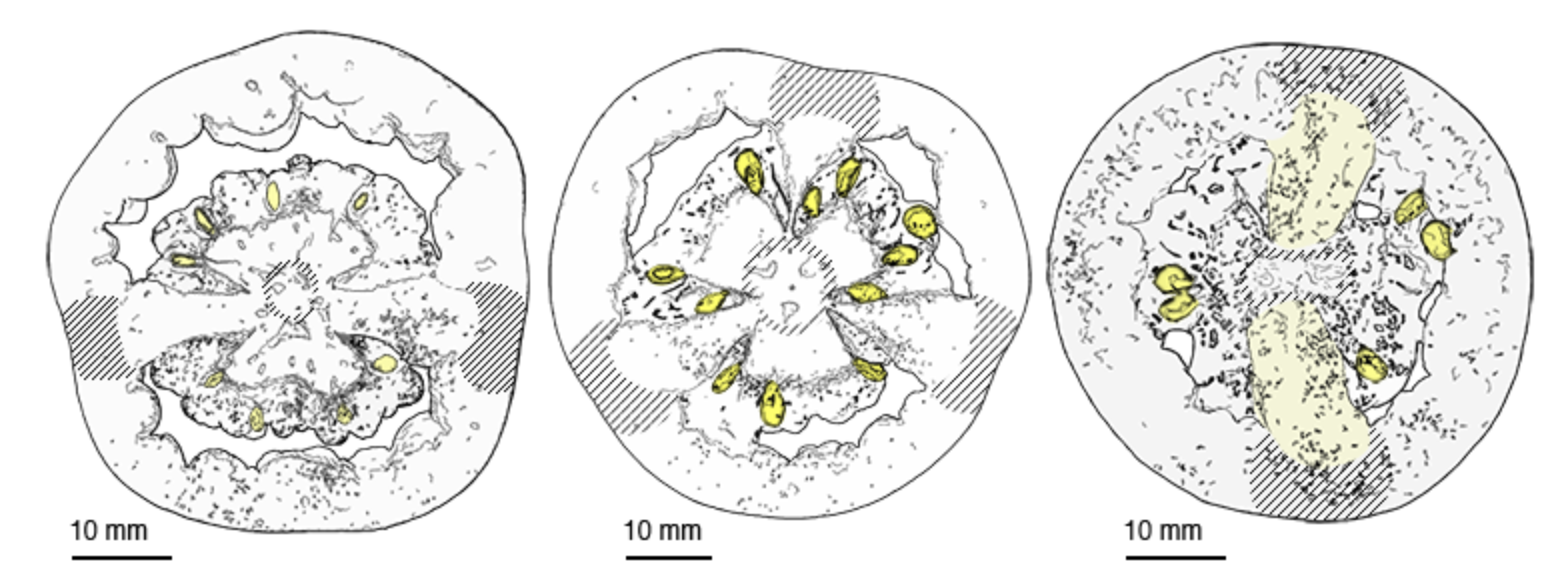

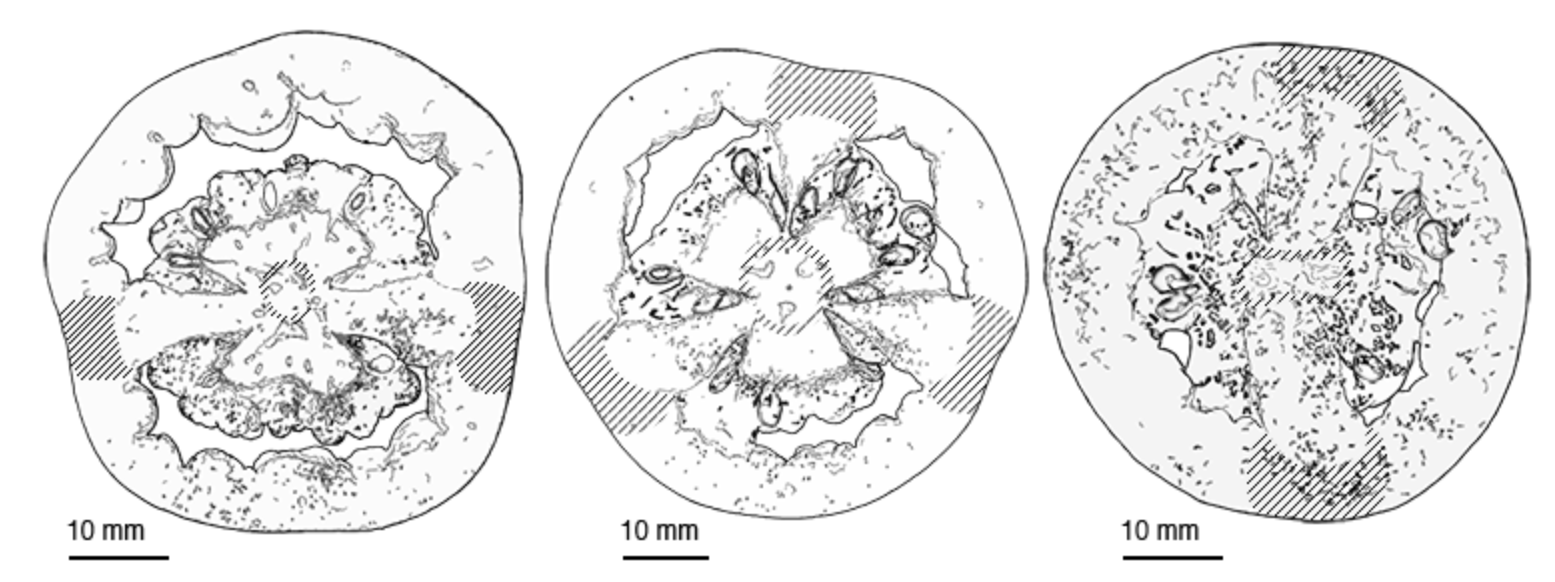

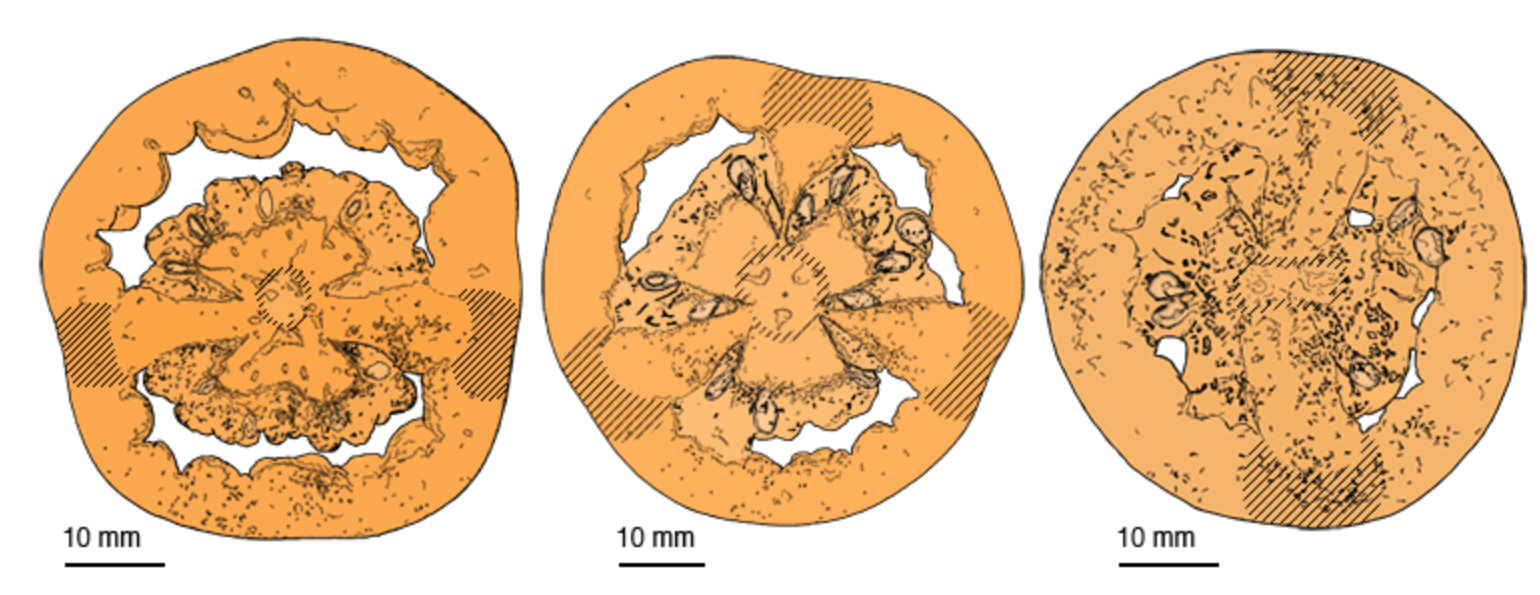

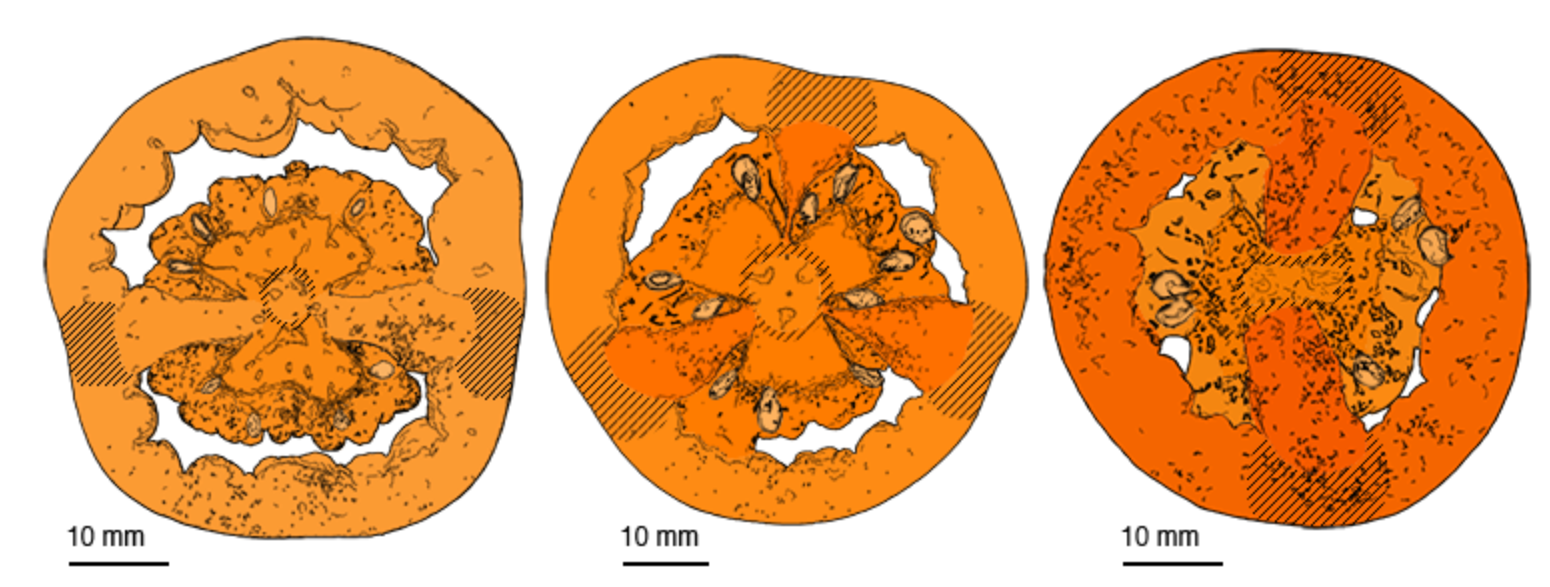

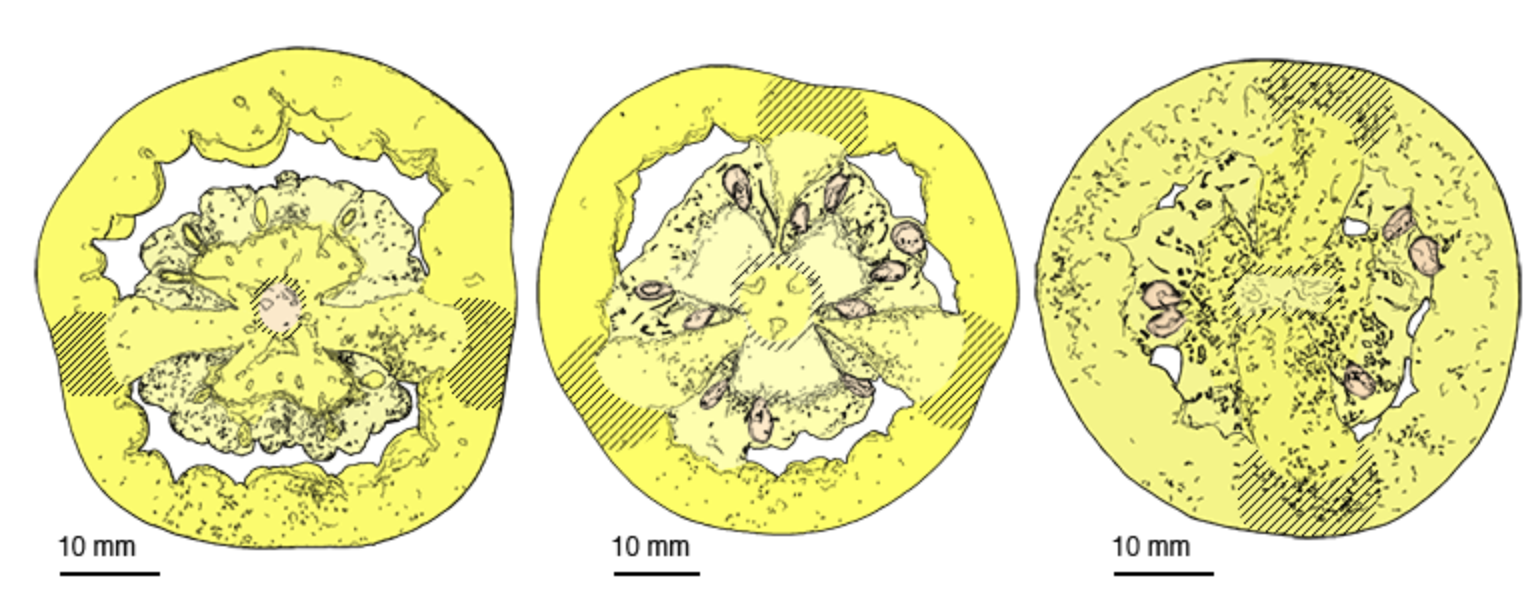

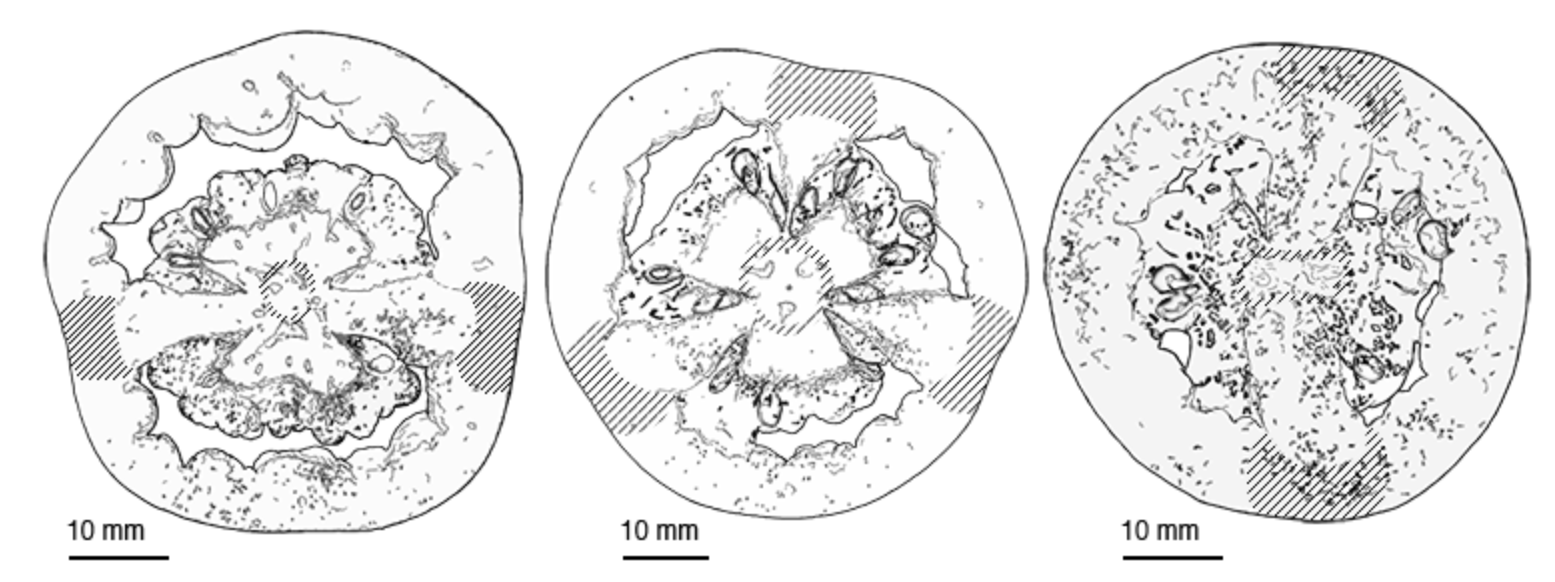

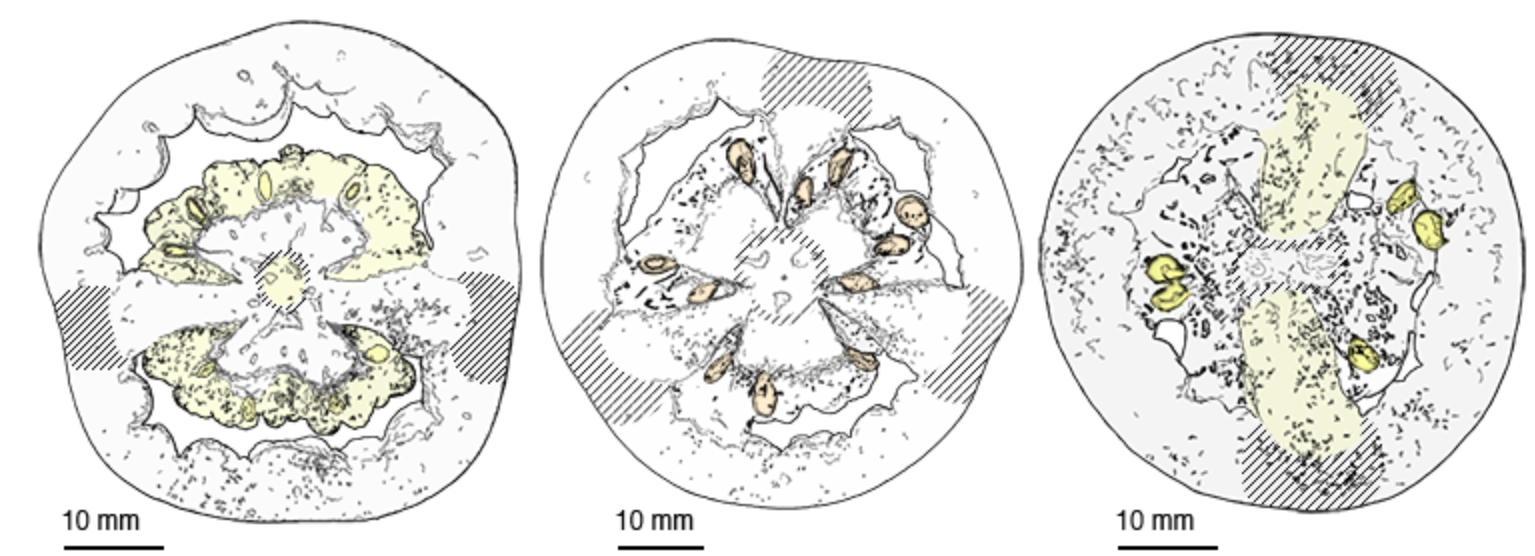

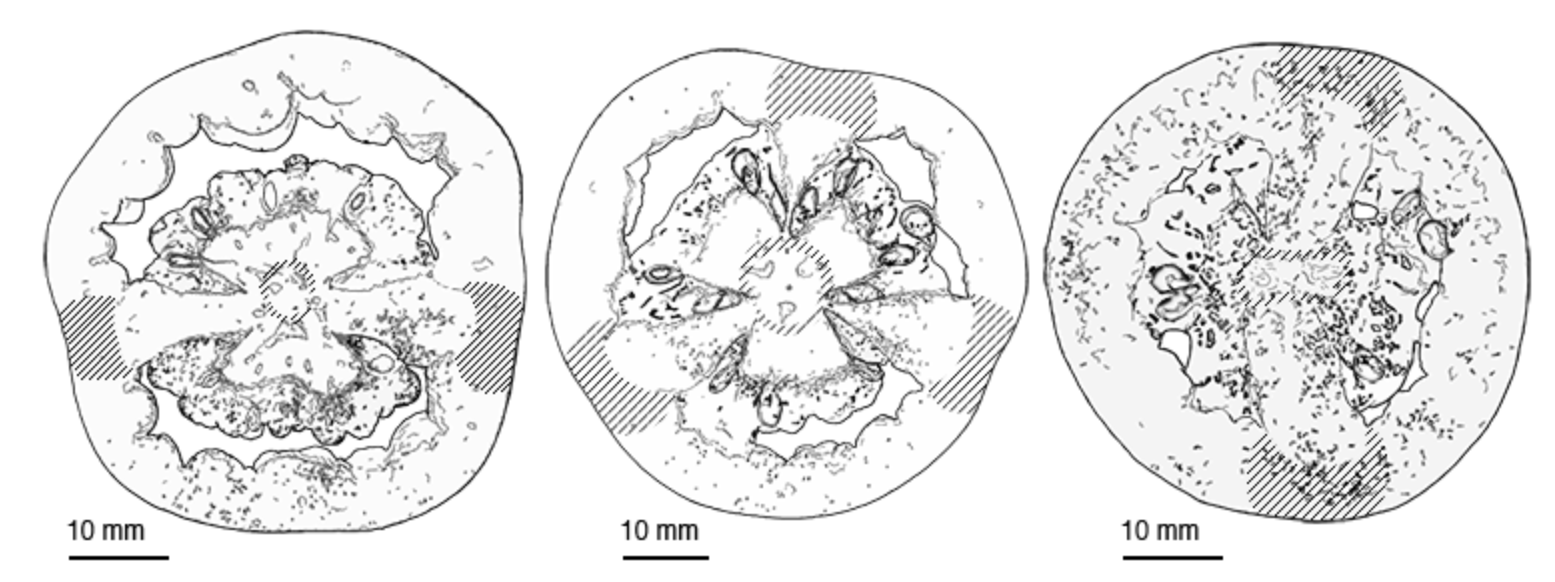


**MG**

**BR**

**RR**

***SlETP1-like***

***SlETP2-like1***

***SlETP2-like2***

***SlETP2-like3***

***SlETP2-like4***

***SlETP2-like5***

***SlETP2-like6***

***SlETP2-like7***

***SlETP2-like8***


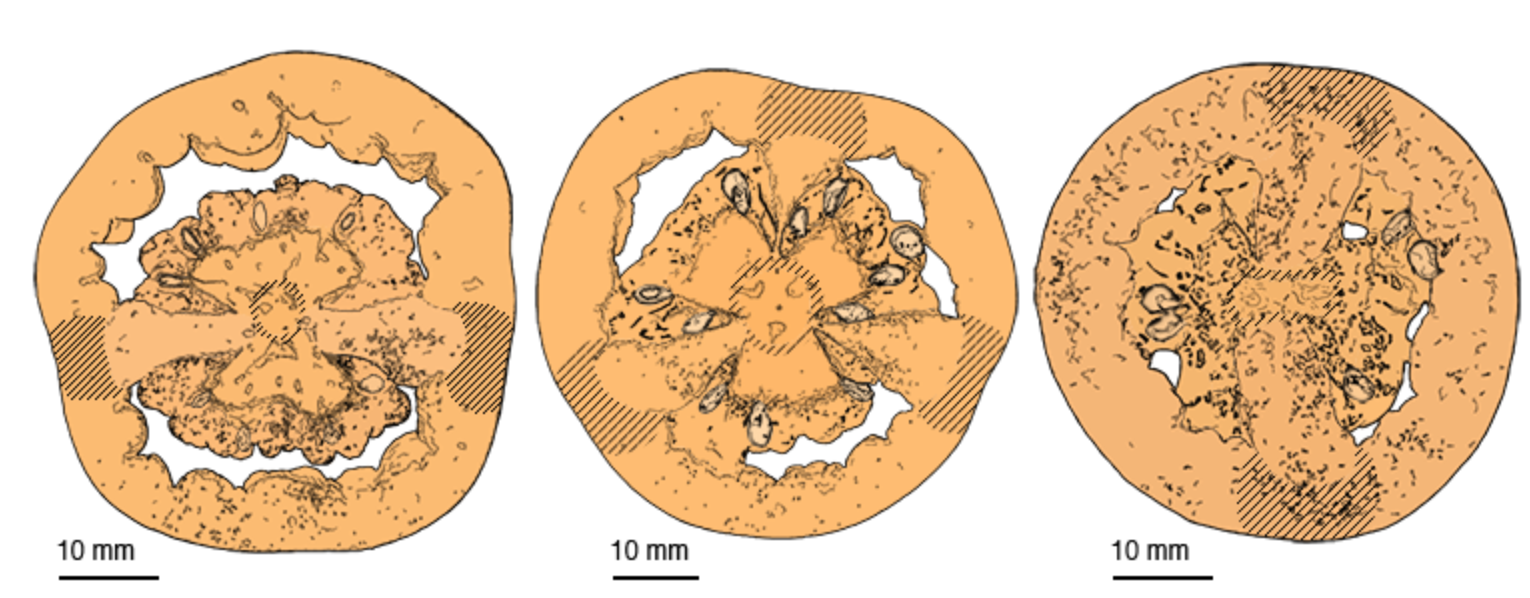


**b**

**Supplemental Figure S5 Phylogenetic analysis of SlETPs-like proteins and their transcriptional expression images and interaction of these proteins with YFT1-C**.

**BD-SlETP2-like3**

**BD-SlETP1-like**

**BD-SlETP2-like2**

**-4SD**

**c**

**AD-YFT1-C**


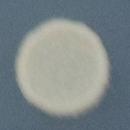

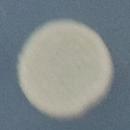

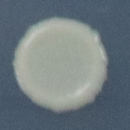

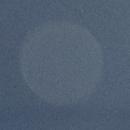

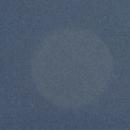

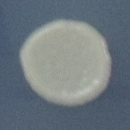

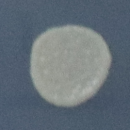

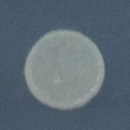

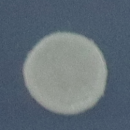

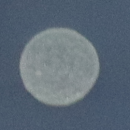

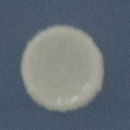

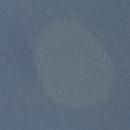


**-2SD**

**-3SD**

**BD**

**(a)** Amino acid sequences of the AtETP1/2([NP_001078185.1](https://www.ncbi.nlm.nih.gov/protein/NP_001078185.1?report=genbank&log$=prottop&blast_rank=1&RID=MRUSVVNT016)/[NP_188572.1](https://www.ncbi.nlm.nih.gov/protein/NP_188572.1?report=genbank&log$=prottop&blast_rank=1&RID=MRUX5NHU016), *AT3G18980*/ *AT3G19410*) derived from Arabidopsis were submitted to web site of <https://blast.ncbi.nlm.nih.gov/Blast.cgi>, to search homologues in *Solanum lycopersicm* based on the blast function, and captured total nine SlETP1/2-likes. In which, SlETP1-like, [XP_004247952.1](https://www.ncbi.nlm.nih.gov/protein/XP_004247952.1?report=genbank&log$=prottop&blast_rank=1&RID=MRUGZ95R016) (*Solyc09g091690.1.1*); SlETP2-like1, [XP_004249778.4](https://www.ncbi.nlm.nih.gov/protein/XP_004249778.4?report=genbank&log$=prottop&blast_rank=1&RID=MDR43DWJ013) (*Solyc10g082010.1.1*); SlETP2-like2, [XP_004239218.2](https://www.ncbi.nlm.nih.gov/protein/XP_004239218.2?report=genbank&log$=prottop&blast_rank=2&RID=MDR43DWJ013)(*Solyc05g015520.3.1*); SlETP2-like3, [NP_001335104.1](https://www.ncbi.nlm.nih.gov/protein/NP_001335104.1?report=genbank&log$=prottop&blast_rank=3&RID=MDR43DWJ013)(*Solyc02g089310.1.1*); SlETP2-like4, [XP_004247554.1](https://www.ncbi.nlm.nih.gov/protein/XP_004247554.1?report=genbank&log$=prottop&blast_rank=4&RID=MDR43DWJ013)(*Solyc09g072930.1.1*); SlETP2-like5, [XP_025883623.1](https://www.ncbi.nlm.nih.gov/protein/XP_025883623.1?report=genbank&log$=prottop&blast_rank=5&RID=MDR43DWJ013) (*Solyc10g009450.1.1*); SlETP2-like6, [XP_069150445.1](https://www.ncbi.nlm.nih.gov/protein/XP_069150445.1?report=genbank&log$=prottop&blast_rank=6&RID=MDR43DWJ013)(*Solyc09g091680.1.1*); SlETP2-like7, [XP_004247228.1](https://www.ncbi.nlm.nih.gov/protein/XP_004247228.1?report=genbank&log$=prottop&blast_rank=7&RID=MDR43DWJ013)(*Solyc09g066210.4.1*); SlETP2-like8, [XP_025883690.1](https://www.ncbi.nlm.nih.gov/protein/XP_025883690.1?report=genbank&log$=prottop&blast_rank=8&RID=MDR43DWJ013) (*Solyc10g009460.1.1*).

All amino acid sequences were submitted to MEGA 11 software to draw a phylogenetic tree by neighbor-joining, and the bootstrap values represent the percentage of 1000 replicates. The length of branches is genetic distance. The amino acid ID number in red highlight was chosen in this study.

**(b)Tissue-based** **expression images of *SlETPs* genes**

The IDs of the *SlETPs* were submitted to the Tomato Expression Atlas platform (<https://tea.solgenomics.net/>) to obtain their expression data. RPM, reads per million mapped reads. Scale bars, 10mm

**(c) Identified the interaction of YFT1-C with SlETP2-like3**.

Of all nine *SlETPs*, the especially expressed genes in tomato fruit include *SlETP1-like* (*Solyc09g091690.1.1*), *SlETP2-like2*(*Solyc05g015520.3.1*), and *SlETP2-like3*(*Solyc02g089310.1.1*), and in which, only SlETP2-like3 was identified to interact with YFT1-C by Y2H assay on the solid media of -2SD, -3SD, and -4SD.

**a**

**b**


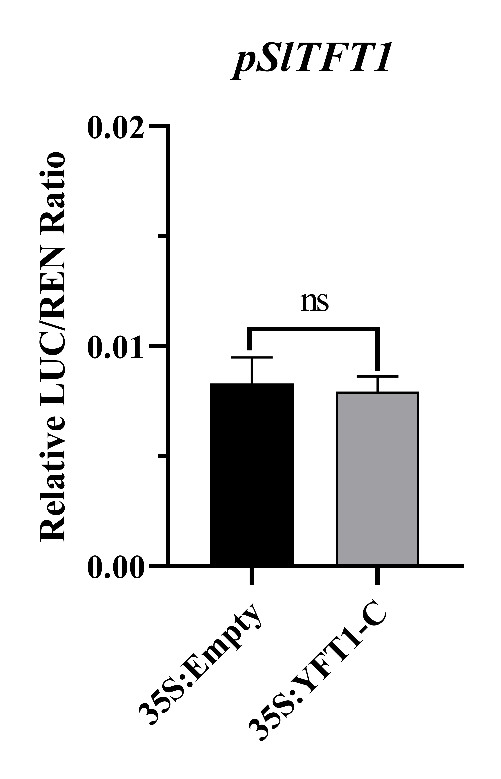

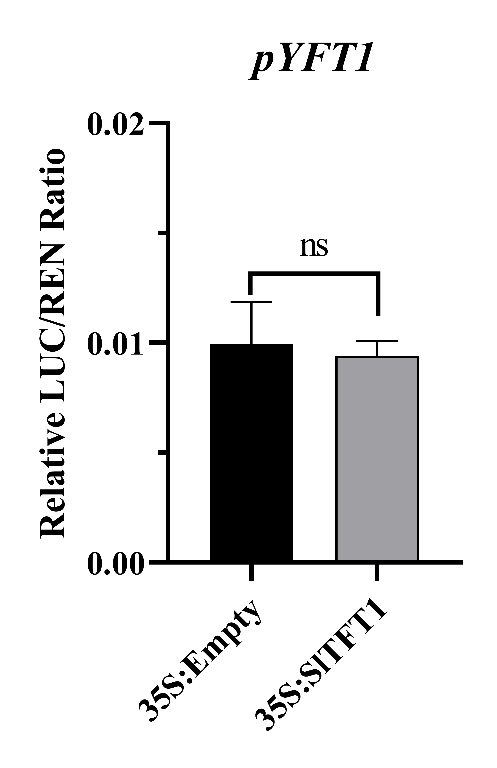


**g**

**c**

**d**

**e**

**f**

**Supplemental Figure S6** **Detection of interaction between *SlTFT1* and *YFT1* by DLR and EMSA.**

**(a) and (b) Dual-luciferase reporter(DLR) assay**

The luciferase activities were quantified in *35S*::*SlTFT1-GFP*/*35S*::*REN*-*pYFT1::LUC*(a) and *35S*:*:YFT1-C-GFP* /*35S*::*REN*-*pSlTFT1*::*LUC*(b)

**(c-g) Electrophoretic mobility shift assay (EMSA).**

**(c)** positive control, the interaction between OsERF34 and the DNA fragment 5′-cgcacgtaccgacacagccg-3′which located at the promoter of *Rice Morphology Determinant* (*RMD*) was used as a positive control in present experiment according to Zhang *et al.* (2022). **(d)** Hot probe-*pT*-1, a DNA fragment (-1248 bp to -1206 bp) located in *pSlTFT1*; **(e)**Hot probe-*pT*-2, a DNA fragment (-428 bp to -386 bp) located in *pSlTFT1*; **(f)** Hot probe-*pY*-1, a DNA fragment (-1504 bp to -1462 bp) located in *pYFT1*; **(g)**Hot probe-*pY*-2, a DNA fragment (-604 bp to -562 bp) located in *pYFT1*. These hot probes were labeled by FAM at the 5’-end and the interaction between SlTFT1 and YFT1 was checked by the CY2 channel of a ChemiDoc MP imaging system (BioRad, Hercules, CA, USA). Moreover, a 50-fold excess of nonlabelled probes were used for competition.
