# Supplementary material for: The 14‐3‐3 Protein SlTFT1 Accelerates Tomato Fruit Ripening by Binding and Stabilising YFT1 in the Ethylene Signalling Pathway
Source: Plant Biotechnol J. 2025 Jul 22;23(11):4872–92. doi: 10.1111/pbi.70274 (PMC12576456; doi:10.1111/pbi.70274)
Supplement: Supplementary file 2 — Table S1. Primers used in this study. [file PBI-23-4872-s001.docx]

**Supplementary Table S1 Primers used in this study**

| **Primer ID** | **Primer sequences（5’to 3’）** | **Application** |
| --- | --- | --- |
| TFT-CRI-F | atatatggtctcgtttgacaagctcgtaatcgggtcgttttagagctagaaatagc | CRISPR/Cas 9 |
| TFT-CRI-R | attattggtctcgaaac cgctgctcgaagtgaaccgcaaactacactgttagattc | CRISPR/Cas 9 |
| TFT-TEST-F | ccaaaaacgtcgaccctcgt | CRISPR/Cas 9 |
| TFT-TEST-R | acaccttggattcaccagca | CRISPR/Cas 9 |
| OE-TFF-F | ctctaagcttggatccatggccttgcctgaaaatttaacca | Overexpression |
| OE-TFF-R | gaacgaaagctctaga tcaagcctcgtccatctgctcct | Overexpression |
| OE-TEST-F | atggccttgcctgaaaatttaacca | Overexpression |
| OE-TEST-R | acagttttcccaatgccataatac | Overexpression |
| AD-YFT1-C-F | ggaggccagtgaattctctgatggtccggggtcatacaa | Y2H |
| AD-YFT1-C-R | cgagctcgatggatccttacaagacgaaagggggtgatgagg | Y2H |
| BD-TFT1-F | catggaggccgaattcatggccttgcctgaaaatttaacca | Y2H |
| BD-TFT1-R | gcaggtcgacggatcctcaagcctcgtccatctgctcct | Y2H |
| BD-TFT2-F | catggaggccgaattcatggctcgtgaggagaatgtgt | Y2H |
| BD-TFT2-R | gcaggtcgacggatcctcactgttgttcattgtcgttt | Y2H |
| BD-TFT3-F | catggaggccgaattcatggcggtggcaccgacggcgc | Y2H |
| BD-TFT3-R | gcaggtcgacggatcctcaatttttttcttcaggtttgg | Y2H |
| BD-TFT4-F | catggaggccgaattcatggctgactcttcgcgtgaag | Y2H |
| BD-TFT4-R | gcaggtcgacggatcctcactgctgcccctcgcctgac | Y2H |
| BD-TFT5-F | catggaggccgaattcatggcgtctccacgtgaagaga | Y2H |
| BD-TFT5R | gcaggtcgacggatcccatcaaaagctgataatgagtag | Y2H |
| BD-TFT6-F | catggaggccgaattcatggcgtcgccacgcgaggaaaac | Y2H |
| BD-TFT6-R | gcaggtcgacggatcctcattcattatcatctggtttag | Y2H |
| BD-TFT7-F | catggaggccgaattcatggagaaggaaagagaaaaac | Y2H |
| BD-TFT7-R | gcaggtcgacggatcc ctagttctctccctggcgctca | Y2H |
| BD-TFT8-F | catggaggccgaattcatggcttcatccaaagaacgtg | Y2H |
| BD-TFT8-R | gcaggtcgacggatcctcactccgcatcctcgcctgca | Y2H |
| BD-TFT9-F | catggaggccgaattcatggcttcttccaaagaacgtg | Y2H |
| BD-TFT9-R | gcaggtcgacggatcctcactctgcatcttcacctccac | Y2H |
| BD-TFT10-F | catggaggccgaattcatggcggctctaatccctgaaaatc | Y2H |
| BD-TFT10-R | gcaggtcgacggatcctcaagattcatccaactgatcctg | Y2H |
| BD-TFT12-F | catggaggccgaattcatggcttctcaaaaggaaagag | Y2H |
| BD-TFT12-R | gcaggtcgacggatccctattgtttatcttttgatgcc | Y2H |
| BD-SlETP2-F | catggaggccgaattcatggttgaaatagcggaatc | Y2H |
| BD-SlETP2-R | gcaggtcgacggatccttagtatattgaagcatcaaatc | Y2H |
| BD-Sol05g015520-F | ggacgccggcggatcc atggaagtgttggccatgct | Y2H |
| BD-Sol05g015520-R | cgactctagaggatcc tcaagccacagctgttagacagg | Y2H |
| BD-Sol09g091690-F | ggacgccggcggatccatggaaactcaaaacaaattagctgaagcaaaaattcct | Y2H |
| BD-Sol09g091690-R | cgactctagaggatcc ttacgtatcaccaacattaggcg | Y2H |
| pXY104-YFT1-C-cYFP-F | cggtacccggggatccatg ctgaaatctgcaagttccagg | BiFC |
| pXY104-YFT1-C-cYFP-R | cgactctagaggatcccaagacgaaagggggtgat | BiFC |
| pXY106-nYFP-TFT1-F | ggacgccggcggatccatggccttgcctgaaaattta | BiFC |
| pXY106-nYFP-TFT1-R | cgactctagaggatcctcaagcctcgtccatctgc | BiFC |
| pXY104-YFT1-C1-cYFP-F | cggtacccggggatccatgtctgatggtccggggtcatacaa | BiFC |
| pXY104-YFT1-C1-cYFP-R | cgactctagaggatcccaatggttctgcatagtttgacgt | BiFC |
| pXY104-YFT1-C2-cYFP-F | cggtacccggggatccatggctcgtgtttcggggcaaaaa | BiFC |
| pXY104-YFT1-C2-cYFP-R | cgactctagaggatccgagaaatttctctcttgcagc | BiFC |
| pXY104-YFT1-C3-cYFP-F | cggtacccggggatccatgtatgaagctgaaactagggagata | BiFC |
| pXY104-YFT1-C3-cYFP-R | cgactctagaggatcccaagacgaaagggggtgat | BiFC |
| pXY104-YFT1-C2-1-cYFP-F | cggtacccggggatccatggctcgtgtttcggggcaaaaa | BiFC |
| pXY104-YFT1-C2-1-cYFP-R | cgactctagaggatccagaatcttggcgaggaacata | BiFC |
| pXY104-YFT1-C2-2-cYFP-F | cggtacccggggatccatgatagtgtcagatgcgagagct | BiFC |
| pXY104-YFT1-C2-2-cYFP-R | cgactctagaggatccaccaaattgctcaaaaggctg | BiFC |
| pXY104-YFT1-C2-3-cYFP-F | cggtacccggggatccatggtagctggtaagccagatgtt | BiFC |
| pXY104-YFT1-C2-3-cYFP-R | cgactctagaggatccgagaaatttctctcttgcagc | BiFC |
| pXY104-YFT1-CS287A-cYFP-F1 | cggtacccggggatcctctgatggtccggggtcata | BiFC |
| pXY104-YFT1-CS287A-cYFP-R1 | atcaggcaaggcgtagtacttcttagagttggctga | BiFC |
| pXY104-YFT1-CS287A-cYFP-F2 | tacgccttgcctgatatctcagggcgctatgttcctc | BiFC |
| pXY104-YFT1-CS287A-cYFP-R2 | cgactctagaggatccttacaagacgaaagggggtga | BiFC |
| pXY104-YFT1-CS287D-cYFP-F1 | cggtacccggggatcctctgatggtccggggtcata | BiFC |
| pXY104-YFT1-CS287D-cYFP-R1 | atcaggcaagtcgtagtacttcttagagttggctga | BiFC |
| pXY104-YFT1-CS287D-cYFP-F2 | tacgacttgcctgatatctcagggcgctatgttcctc | BiFC |
| pXY104-YFT1-CS287D-cYFP-R2 | cgactctagaggatccttacaagacgaaagggggtga | BiFC |
| pXY104-YFT1-CS478A-cYFP-F1 | cggtacccggggatcctctgatggtccggggtcata | BiFC |
| pXY104-YFT1-CS478A-cYFP-R1 | ttttggggcggcaccgggtttcctgttggaagagaag | BiFC |
| pXY104-YFT1-CS478A-cYFP-F2 | ggtgccgccccaaaacctgaagagatggattacaccaa | BiFC |
| pXY104-YFT1-CS478A-cYFP-R2 | cgactctagaggatccttacaagacgaaagggggtga | BiFC |
| pXY104-YFT1-CS478D-cYFP-F1 | cggtacccggggatcctctgatggtccggggtcata | BiFC |
| pXY104-YFT1-CS478D-cYFP-R1 | ttttggggcgtcaccgggtttcctgttggaagagaag | BiFC |
| pXY104-YFT1-CS478D-cYFP-F2 | ggtgacgccccaaaacctgaagagatggattacaccaa | BiFC |
| pXY104-YFT1-CS478D-cYFP-R2 | cgactctagaggatccttacaagacgaaagggggtga | BiFC |
| pXY104-YFT1-C2-1-1-cYFP-F | cggtacccggggatccatggctcgtgtttcggggcaaaaa | BiFC |
| pXY104-YFT1-C2-1-1-cYFP-R | cgactctagaggatccgttagtgggctgcatcgaatt | BiFC |
| pXY104-YFT1-C2-1-2-cYFP-F | cggtacccggggatccatgactacttccgtcgaccatagct | BiFC |
| pXY104-YFT1-C2-1-2-cYFP-R | cgactctagaggatccagaatcttggcgaggaacata | BiFC |
| pXY104-YFT1-C2-3-1-cYFP-F | cggtacccggggatccatggtagctggtaagccagatgtt | BiFC |
| pXY104-YFT1-C2-3-1-cYFP-R | cgactctagaggatccctgaagcagcttagcttcca | BiFC |
| pXY104-YFT1-C2-3-2-cYFP-F | cggtacccggggatccatgtctttcagaagttgtattgtga | BiFC |
| pXY104-YFT1-C2-3-2-cYFP-R | cgactctagaggatccgagaaatttctctcttgcagc | BiFC |
| pXY104-YFT1-C2-1-2-7A-cYFP-F | cggtacccggggatccatggctgctgccgtcgaccatagc | BiFC |
| pXY104-YFT1-C2-1-2-7A-cYFP-R | ttagcgttggctgcacc | BiFC |
| pXY104-YFT1-C2-1-2-10A-cYFP-F | ggtgcagccaacgctaa | BiFC |
| pXY104-YFT1-C2-1-2-10AcYFP-R | cgactctagaggatccagcatcttggcgaggaacagcgcgc | BiFC |
| YFT1-C-cLUC-F | gcgtcccggggcggtaccatgtctgatggtccggggtcatacaa | Split-Luc |
| YFT1-C-cLUC-R | tccatttgttggatccttacaagacgaaagggggtgatgagg | Split-Luc |
| TFT1-nLUC-F | cggtacccgggatccatggccttgcctgaaaatttaacc | Split-Luc |
| TFT1-nLUC-R | gtacgagatctggtcgacagcctcgtccatctgctcctg | Split-Luc |
| pZX34-TFT1-YFP-F | ggactcttgaccatggccttgcctgaaaatttaacca | Subcelluar localization |
| pZX34-TFT1-YFP-R | tgctcaccatactagtagcctcgtccatctgctcct | Subcelluar localization |
| pZX34-YFT1-C-YFP-F | ggactcttgaccatgggctctgatggtccggggtcatacaa | Subcelluar localization |
| pZX34-YFT1-C-YFP-R | tgctcaccatactagtcaagacgaaagggggtgatgagg | Subcelluar localization |
| TFT1-6HA-F | gctacgcgtctcgagatggccttgcctgaaaatttaacca | CoIP |
| TFT1-6HA-R | gggctgcaggaattcagcctcgtccatctgctcctgcata | CoIP |
| YFT1-C-GFP-F | ggactcttgaccatgggctctgatggtccggggtcatacaa | CoIP |
| YFT1-C-GFP-R | tgctcaccatactagtcaagacgaaagggggtgatgagg | CoIP |
| ETP-F | tctctcaagcttggatccatggttgaaatagcggaatc | CoIP |
| ETP-T2A-R | caggcaaggccatgggtcctgggttctc | CoIP |
| TFT1-T2A-F | gagaacccaggacccatggccttgcctgaaaattt | CoIP |
| T2A-CEND-F | gaagagaacccaggaccctctgatggtccggggtca | CoIP |
| CEND-R | tactagttctggatcccaagacgaaagggggtgatgaggc | CoIP |
| T2A-GFP | tactagttctggatccgggtcctgggttctcttccac | CoIP |
| ACTIN-F | ttgctgaccgtatgagcaag | qRT-PCR |
| ACTIN-R | ggacaatggatggaccagac | qRT-PCR |
| TFT1-F | tgtttgtgctggaattctgaag | qRT-PCR |
| TFT1-R | gcatgctttttctgatgcattc | qRT-PCR |
| TFT2-F | gaatttaagaccggagctgaac | qRT-PCR |
| TFT2-R | aagattcttctcccagtgtgtc | qRT-PCR |
| TFT3-F | gagaggtatgaagagatggtgg | qRT-PCR |
| TFT3-R | gatcacattcttatacgcgacg | qRT-PCR |
| TFT4-F | atcttctttcagtggcctacaa | qRT-PCR |
| TFT4-R | aggtacctatggtagtcacctt | qRT-PCR |
| TFT5-F | gcatcgtggcgtataatttcat | qRT-PCR |
| TFT5-R | atttcttaatggaggcaacgtg | qRT-PCR |
| TFT6-F | aagctgctgagaatactctctc | qRT-PCR |
| TFT6-R | gcacgatcaggagaattcaaaa | qRT-PCR |
| TFT7-F | catttgatgaagctattgccga | qRT-PCR |
| TFT7-R | ccataagaagtacgtacctccc | qRT-PCR |
| TFT8-F | caaagaacgtgaaagcttggta | qRT-PCR |
| TFT8-R | actgtcagttcaacatccagat | qRT-PCR |
| TFT9-F | cagagtgaatgggcctaatagt | qRT-PCR |
| TFT9-R | tgacacgacagacccataatag | qRT-PCR |
| TFT10-F | tgtgtttgactccacgtgtata | qRT-PCR |
| TFT10-R | tgagaaaaagagagagaagggac | qRT-PCR |
| TFT12-F | gtgactattttcgttacctcgc | qRT-PCR |
| TFT12-R | cagagaagttcaaagcaagacc | qRT-PCR |
| PSY1-F | tggcccaaacgcatcatata | qRT-PCR |
| PSY1-R | caccatcgagcatgtcaaatg | qRT-PCR |
| LCYE-F | actggatttagtggtaatcggctgt | qRT-PCR |
| LCYE-R | agttgtttgtgaaaggaagatcagg | qRT-PCR |
| CYCB-F | tgttattgaggaagagaaatgtgtgat | qRT-PCR |
| CYCB-R | tcccaccaatagccataacatttt | qRT-PCR |
| ACO1-F | tggtgaccaacttgaggtga | qRT-PCR |
| ACO1-R | caattggatcactttccattgcc | qRT-PCR |
| ACS2-F | gtggtgccactggagctaat | qRT-PCR |
| ACS2-R | gtccaaagtggtgcccaatg | qRT-PCR |
| ACS4-F | atgggtctcgcggaaaatca | qRT-PCR |
| ACS4-R | aagcatcaccaggatcagcc | qRT-PCR |
| YFT1-F | actgcggagaaggttgtg | qRT-PCR |
| YFT1-R | atggctcgtcggagaatg | qRT-PCR |
| SlEBF1-F | attgccatcactgacatagc | qRT-PCR |
| SlEBF1-R | agttatagcaagcgacctc | qRT-PCR |
| SlEBF2-F | atgtgatggataccttaccag | qRT-PCR |
| SlEBF2-R | ccgacattagtaataccacga | qRT-PCR |
| SlEBF3-F | ccaaggcaggactcgtcaag | qRT-PCR |
| SlEBF3-R | ccaaagtttcaccgtgtagcc | qRT-PCR |
| NR-F | tgctgttcgtgtaccgcttt | qRT-PCR |
| NR-R | tcatcgggagaaccagaacc | qRT-PCR |
| SlETR4-F | tggaggagtgagtgtggatgc | qRT-PCR |
| SlETR4-R | atggctgtcgttcttgggc | qRT-PCR |
| SlEIL1-F | caagggataatggacggaaata | qRT-PCR |
| SlEIL1-R | tgaaatggaagttgtcgttgg | qRT-PCR |
| SlEIL2-F | tgaagatgatggaagtctgtaagg | qRT-PCR |
| SlEIL2-R | ccactccctgagattatccga | qRT-PCR |
| SlEIL3-F | ccttcggtggaaacataaatga | qRT-PCR |
| SlEIL3-R | caacctatcaagccttcatatag | qRT-PCR |
| SlEIL4-F | gcctccattcaacttacagtcc | qRT-PCR |
| SlEIL4-R | ggaatatcttgcttcggtgtgg | qRT-PCR |
| SlGLUTR-F | gcaagatggggaaacttgtg | qRT-PCR |
| SlGLUTR-R | catttcattgaggggcttgt | qRT-PCR |
| SlGUN5-F | cgaaggagttatgcaaataccattagc | qRT-PCR |
| SlGUN5-R | ctcactaagctgcttgagtccctt | qRT-PCR |
| RIN-F | aacatcatggcattgtggtg | qRT-PCR |
| RIN-R | gtgttgatggtgctgcattt | qRT-PCR |
| NOR-F | agagaacgatgcatggaggtttgt | qRT-PCR |
| NOR-R | actggctcaggaaattggcaatgg | qRT-PCR |
| FUL1-F | gttttgccacaacaactggactc | qRT-PCR |
| FUL1-R | cttgctgctgtgaagaactacc | qRT-PCR |
| 0000-SlTFT1-F | taagcttgatatcgaattcatggccttgcctgaaaatttaacc | DLR |
| 0000-SlTFT1-R | atctagaactagtggatcctcagtacgagatctggtcgac | DLR |
| 0000-YFT1-C-F | taagcttgatatcgaattcatgtctgatggtccggggtcatacaa | DLR |
| 0000-YFT1-C-R | atctagaactagtggatccttacaagacgaaagggggtgatgagg | DLR |
| 0000-SlEIL1-F | taagcttgatatcgaattcatgatgatgtttgaggaaat | DLR |
| 0000-SlEIL1-R | atctagaactagtggatccctagtaccaaataggagcat | DLR |
| 0800-RIN-F | cggtatcgataagcttaggtaagtggacactaccaaa | DLR |
| 0800-RIN-R | attcgatatcaagctttggttccctagtcaagaaga | DLR |
| 0800-NOR-F | cggtatcgataagcttggtcatgtgaaggatatgtg | DLR |
| 0800-NOR-R | attcgatatcaagctttatggtataaggcaacactg | DLR |
| 0800-FUL1-F | cggtatcgataagcttacgtcaaggggataattcct | DLR |
| 0800-FUL1-R | attcgatatcaagcttcagtagacaatagccgtgcgc | DLR |
| 0800-SlTFT1-F | cggtatcgataagcttaacatcatgcatctgtaaactcc | DLR |
| 0800-SlTFT1-R | attcgatatcaagcttagctagcttcgccaagtaca | DLR |
| 0800-YFT1-F | cggtatcgataagctttgctctccaagaaagtatttttcaa | DLR |
| 0800-YFT1-R | attcgatatcaagcttctgcagctccaactgtagac | DLR |
| pSlTFT1-probe1 | gttagaatatcagctataaatgtagtcgaattcaataatttt | EMSA |
| pSlTFT1-probe2 | taattcaacaaaataaatgatgtattttttttttatatattc | EMSA |
| pYFT1-probe1 | ccatctctctaggatatgtattatgtacaggttcctctcttt | EMSA |
| pYFT1-probe2 | cttttaactggaattaacatgtatatagttttagtttggata | EMSA |
